# Supplementary material for: Novel 3,9-Disubstituted Acridines with Strong Inhibition Activity against Topoisomerase I: Synthesis, Biological Evaluation and Molecular Docking Study
Source: Molecules. 2023 Jan 30;28(3):1308. doi: 10.3390/molecules28031308 (PMC9921529; doi:10.3390/molecules28031308)
Supplement: Supplementary file 1 [file molecules-28-01308-s001.zip › molecules-2183718-supplementaryV3.pdf]

Novel 3,9-Disubstituted Acridines with Strong Inhibition Activity against Topoisomerase I: Synthesis, Biological Evaluation and Molecular Docking Study

Kristína Krochtová[1], Annamária Halečková[2], Ladislav Janovec[2,*], Michaela Blizniaková[1], Katarína Kušnírová[2], Mária Kožurková[1,*]

[1]Department of Biochemistry, Faculty of Science, P.J. Šafárik University in Košice, Moyzesova 11, 040 01 Košice, Slovakia

[2]Department of Organic Chemistry, Faculty of Science, P.J. Šafárik University in Košice, Moyzesova 11, 040 01, Košice, Slovakia

*Correspondence: maria.kozurkova@upjs.sk; ladislav.janovec@upjs.sk

Supplementary Material

SYNTHESIS

*Synthesis of N-(3-chloro-4-methylphenyl)acetamide (**10**) [1]*

A solution of acetyl chloride (0.83 g, 10.59 mmol) in dry dichloromethane (7 mL) was slowly added dropwise to a flask with a solution of 3-chloro-4-methylaniline (**9**) (1 g, 7.06 mmol) and triethylamine (1.43 g, 14.12 mmol) in dry dichloromethane (10 mL) over 30 minutes. The progress of the reaction was monitored using TLC chromatography where a mixture of toluene/acetone (5:2 – v/v) was used as an eluent. After the completion of the reaction, methanol (1 ml) was added to the reaction mixture to react off the excess of acetyl chloride. The resultant mixture was concentrated in vacuo, and the residue was dissolved in methanol and poured slowly into water (50 mL) to precipitate off. The pale yellow product of *N*-(3-chloro-4-methylphenyl)acetamide (**10**) was filtered off and crystallized from ethanol. The reaction yield was 96%, R_f=0.75 (Ethyl acetate); m.p. 105–106°C; ^{1}H NMR (400 MHz, DMSO–d_6): δ=10.01 (s, 1H; NH), 7.78 (d, *J* = 2.0 Hz, 1H; H2), 7.33 (dd, *J* = 8.4, 2.0 Hz, 1H; H6), 7.23 (d, *J* = 8.4 Hz, 1H; H5), 2.25 (s, 3H; CH_3), 2.03 ppm (s, 3H; $COCH_3$); ^{13}C NMR (100 MHz, DMSO–d_6): δ 168.3 (CO), 138.4 (C1), 132.8 (C3), 131.0 (C5), 129.4 (C4), 118.8 (C2), 117.5 (C6), 23.9 ($\underline{CH_3}CO$), 18.8 ppm (CH_3).

*Synthesis of 4-acetamido-2-chlorobenzoic acid (**11**) [1]*

N-(3-chloro-4-methylphenyl)acetamide (**10**) (1 g, 5.45 mmol) was added in small portions to a solution of $KMnO_4$ (2.58 g, 16.34 mmol) in 100 mL of water. The reaction mixture was vigorously stirred and refluxed over 2 hours. The reaction was monitored using TLC and a mixture of toluene/acetone (3:2) was used as an eluent. After the completion of the reaction, the hot reaction mixture was filtered off to remove MnO_2. Product 11 was then precipitated out of its solution through the addition of 10 % HCl. After being suctioned off and dried out, product **11** was crystalized from ethanol. The reaction yield was 66%; R_f=0.53 (Ethyl acetate); m.p. 208–210°C; ^{1}H NMR (400 MHz, DMSO–d_6): δ=10.35 (s, 1H; NH), 7.88 (d, *J* = 2.0 Hz, 1H; H3), 7.83 (d, *J* = 8.4 Hz, 1H; H6), 7.53 (dd, *J* = 8.4, 2,0 Hz, 1H; H5), 2.09 (s, 3H; CH_3); ^{13}C NMR (100 MHz, DMSO–d_6): δ=169.0 (CO), 165.8 (COOH), 142.8 (C4), 132.9 (C2), 132.3 (C3), 124.3 (C1), 119.9 (C6), 116.7 (C5), 24.1 ppm (CH_3).

*Synthesis of 4-acetamido-2-anilinobenzoic acid (**12**)*

Copper oxide (0.09 g, 0.60 mmol), activated copper (0.03 g, 0.50 mmol) and anhydrous potassium carbonate (0.33 g, 2.40 mmol) were added to a flask containing a mixture of 4-(acetylamino)-2-chlorobenzoic acid (**10**) (1 g, 4.70 mmol) and aniline (1 g, 10.70 mmol) in 13 mL of 2-ethoxyethanol. The reaction was carried out for 4 hours under reflux and vigorous stirring in an oil bath at 120–130°C. After the completion of the reaction, the reaction mixture was diluted with 30 mL of water to give a heterogeneous mixture which was basified to pH > 7 using a concentrated sodium hydroxide solution. After suction filtration through a Buchner funnel, the insoluble residue was washed with 10 ml of water and the filtrate was placed in an ice bath. After cooling, the solution was acidified to pH < 7 with concentrated hydrochloric acid to precipitate product **12**. After suction filtration, the grey product was washed with water and allowed to dry freely. The reaction yield was 70%; m.p. 225–227°C; ^{1}H NMR (600 MHz, DMSO–d_6): δ=10.07 (s, 1H; NHCO), 9.74 (s, 1H; NH), 7.82 (d, *J* = 8.7 Hz, 1H; H6), 7.65 (d, *J* = 2.0 Hz, 1H; H3), 7.36 (dd, *J* = 8.4, 7.3 Hz, 2H; H3´, H5´), 7.28–7.22 (m, 2H; H2´, H6´), 7.11–7.04(m, 1H; H4´), 7.02 (dd, *J* = 8.8, 2,0 Hz, 1H; H5), 2.02 ppm (s, 3H; CH_3); ^{13}C NMR (150 MHz, DMSO–d_6): δ=169.6 (COOH), 168.8 (CO), 147.8 (C2), 144.4 (C4), 140.4 (C1´), 132.8 (C6), 129.4 (C3´,C5´), 123.0 (C4´), 121.4 (C2´,C6´), 108.4 (C5), 107.1 (C1), 102.4 (C3), 24.1 ppm (CH_3); HRMS (ESI): *m/z* calcd for $C_{15}H_{14}N_2O_3+H^+$: 271.10772 $[M+H]^+$; found: 271.10768.

*Synthesis of 3-aminoacridin-9(10H)-one (**13**)*

A viscous mixture of 4-(acetylamino)-2-(phenylamino)benzoic acid (**12**) (1 g, 3.7 mmol) and polyphosphoric acid (10 g) was heated at 100°C of an oil bath. After 1.5 hours, the reaction mixture was cooled to room temperature, diluted with 10 mL of water and heated for 1.5 hour. The course of the hydrolysis was monitored using TLC (eluent – ethyl acetate). After cooling to room temperature, the reaction mixture was poured into 30 mL of water and the resultant solution was alkalized to pH 8 – 9 by the addition of concentrated sodium hydroxide. The product was then filtered off, washed with water to pH 7 and allowed to dry in the air. Product weight 0.78 g; yield 90%; Dark grey crystalline solid, R_f=0.41 (Ethyl acetate); m.p. 245–247°C; ^{1}H

NMR (600 MHz, DMSO–d_6): δ=11.11 (s, 1H; N10H), 8.12 (dd, *J* = 8.0, 1.6 Hz, 1H; H8), 7.90 (d, *J* = 8.7 Hz, 1H; H1), 7.58 (ddd, *J* = 8.4, 6.9, 1.6 Hz, 1H; H6), 7.40–7.37 (m, 1H; H5), 7.12 (ddd, *J* = 8.0, 6.9, 1.1 Hz, 1H; H7), 6.53 (dd, *J* = 8.7, 2.0 Hz, 1H; H2), 6.43 (d, *J* = 2.0 Hz, 1H; H4), 6.11 ppm (s, 2H; NH_2); ^{13}C NMR (150 MHz, DMSO–d_6): δ=175.1 (CO), 153.7 (C3), 143.3 (C4a), 140.9 (C10a), 132.2 (C6), 127.6 (C1), 125.8 (C8), 120.6 (C8a), 120.1 (C7), 116.5 (C5), 111.9 (C9a), 111.3 (C2), 95.6 (C4); HRMS (ESI): *m/z* calcd for $C_{13}H_{10}N_2O+H^+$: 211.08659 [*M*+H]$^+$; found 211.08647.

*Synthesis of 3-chloro-N-(9-oxo-9,10-dihydroacridin-3-yl)propanamide (**14**)*

A mixture of dry 3-aminoacridin-9(10*H*)-one (**13**) (0.3 g, 1.4 mmol) and 3-chloropropionylchloride (2 mL) was heated at 130 °C in an oil bath for two hours under nitrogen. The reaction was monitored using TLC chromatography (eluent - ethyl acetate). After the completion of the reaction, the reaction mixture was cooled to room temperature and diluted with 5 mL of ethyl acetate to precipitate product **14**. After filtration and drying, the product was suspended in 20 mL of water and neutralized by the subsequent addition of $NaHCO_3$. After filtration, the product was left to dry in the air. The product was crystallized from ethanol. Product weight 0.36 g; yield 85%; dark yellow crystalline solid; R_f = 0.52 (Ethyl acetate); m.p. 290–292°C; ^{1}H NMR (600 MHz, DMSO–d_6): δ = 11.95 (s, 1H; NH10), 10.78 (s, 1H; CONH), 8.21 (d, *J* = 1.6 Hz, 1H; H8), 8.18 (d, *J* = 1.5 Hz, 1H; H4), 8.14 (d, *J* = 8.8 Hz, 1H; H1), 7.70–7.65 (m, 1H; H6), 7.57 (d, *J* = 8.4 Hz, 1H; H5), 7.33 (dd, *J* = 8.8, 1.5 Hz, 1H; H,), 7.23–7.19 (m, 1H; H7), 3,92 (t, *J* = 6.2 Hz, 2H; CH_2Cl), 2.95 ppm (t, *J* = 6.2 Hz, 2H; $COCH_2$); ^{13}C NMR (150 MHz, DMSO–d_6): δ=175.9 (CO), 160.0 (NH–CO), 143.3 (C3), 142.0 (C10a), 141.2 (C4a), 133.1 (C6), 127.0 (C1), 125.9 (C8), 120.9 (C7), 120.6 (C8a), 117.3 (C5), 116.5 (C9a), 113.6 (C2), 105.0 (C4), 40.8 (CH_2Cl), 39.4 ppm ($COCH_2$); HRMS (ESI): *m/z* calcd for $C_{16}H_{13}ClN_2O+H^+$: 301.07383 [*M*+H]$^+$; found 301.07468.

*Synthesis of N-(9-oxo-9,10-dihydroacridin-3-yl)-3-(pyrrolidin-1-yl)propanamide hydrogen chloride (**15**)*

A mixture of 3-[(3-chloropropanoyl)amino]acridin-9(10*H*)-one (**14**) (0.38 g, 1.3 mmol) and pyrrolidine (0.38 mL, 2.6 mmol) in ethanol was heated at 55°C for one hour under nitrogen. The reaction was monitored using TLC chromatography (eluent - ethyl acetate). After the completion of the reaction, the reaction mixture was cooled to room temperature and the solvent was evaporated *in vacuo*. The crude product was dissolved in 5 mL of ethanol and then 36% hydrochloric acid (0.01 mL) was added. The slow addition of acetone to the resultant mixture led to the precipitation of product **15** in the form of its hydrochloride. The product was then filtered off and washed with acetone and ethyl acetate. Product weight 0.39 g; yield 83%; yellow crystalline solid; R_f = 0.45 (Methanol/26% NH_4OH 30:1); m.p. 165–167°C; ^{1}H NMR (600 MHz, DMSO–d_6): δ = 11.99 (s, 1H; NH10), 11.12 (s, 1H; NH+), 10.97 (s, 1H; CONH), 8.18 (dd, *J* = 8.0, 1,6 Hz, 1H; H8), 8.16 (d, *J* = 1.5 Hz, 1H; H4), 8.13 (d, *J* = 8.8 Hz, 1H; H1), 7.69–7.66 (m, 1H; H6), 7.58 (d, *J* = 8.4 Hz, 1H; H5), 7.36–7.34 (m, 1H; H2), 7.20–7.19 (m, 1H; H7), 3.53–3.47 (m, 2H; $N_{py}\underline{CH_{2B}}CH_2$), 3.46–3.42 (m, 2H; $COCH_2\underline{CH_2}$), 3.10–3.00 (m, 4H; $CO\underline{CH_2}CH_2$, $N_{py}\underline{CH_{2B}}CH_2$), 2.05–1.94 (m, 2H; $N_{py}CH_2\underline{CH_{2A}}$), 1.92–1.83 ppm (m, 2H; $N_{py}CH_2\underline{CH_{2A}}$); ^{13}C NMR (150 MHz, DMSO–d_6): δ=175.9 (CO), 168.7 (NH–CO), 143.3 (C3), 142.0 (C10a), 141.2 (C4a), 133.1 (C6), 127.0 (C1), 125.9 (C8), 120.9 (C7), 120.6 (C8a), 117.3 (C5), 116.5 (C9a), 113.6 (C2), 105.0 (C4), 52.9 ($N_{py}\underline{CH_{2AB}}CH_2$), 49.5 ($COCH_2\underline{CH_2}$), 32.5 ($CO\underline{CH_2}CH_2$), 22.8 ppm ($N_{py}CH_2\underline{CH_{2AB}}$); HRMS (ESI): *m/z* calcd for $C_{20}H_{22}ClN_3O_2-Cl^+$: 336.17065 [*M*-Cl]$^+$; found 336.17151.

*Synthesis of N-(9-chloroacridin-3-yl)-3-(pyrrolidin-1-yl)propanamide (**16**)*

A mixture of 3-{[3-(pyrrolidin-1-yl)propanoyl]amino}acridin-9(10H)-one (**15**) (0.3 g, 0.9 mmol) and phosphoryl chloride (3 mL) was allowed to heat slowly to 110°C under nitrogen. After two hours, 3 mL of phosphoryl chloride was added to the reaction mixture and the reaction aperture was flushed by nitrogen. The reaction was allowed to proceed for an additional two hours and monitoring using TLC chromatography (MeOH/26% ammonia, 30:1). The reaction mixture was then allowed to cool to room temperature and diluted with diethyl ether. The crude product was filtered off, washed well with diethyl ether and then dissolved in water. The aqueous solution of product **16** was neutralized with $NaHCO_3$ and then alkalized with small addition of concentrated sodium hydroxide solution. The resultant aqueous heterogeneous mixture of product **16** was heated to 55°C and extracted with ethyl acetate to isolate crude product **16** which was then purified by crystallization from cyclohexane. Product weight 0.054 g; yield 51%; yellow crystalline solid; R_f = 0.48

(Methanol/26% NH_4OH 30:1); m.p. 140–142°C; ^{1}H NMR (600 MHz, DMSO–d_6): δ = 10.64 (s, 1H; CONH), 8.64 (d, J = 2.1 Hz, 1H; H4), 8.34–8.30 (m, 2H; H1,H8), 8.14–8.11 (m, 1H; H7), 7.89–7.85 (m, 1H; H6), 7.77 (dd, J = 9.3, 2.1 Hz, 1H; H2), 7.70–7.68 (m, 1H; H5), 2.78 (t, J = 7.1 Hz, 2H; $COCH_2\underline{CH_2}$), 2.61 (t, J = 7.1 Hz, 2H; $CO\underline{CH_2}CH_2$), 2.52–2.49 (m, 4H; $N_{py}\underline{CH_2}CH_2$), 1.71–1.67 ppm (m, 4H; $N_{py}CH_2\underline{CH_2}$); ^{13}C NMR (150 MHz, DMSO–d_6): δ=171.2 (CO), 149.5 (C4a), 148.8 (C10a), 141.0 (C3), 139.3 (C9), 131.0 (C6), 129.2 (C7), 126.8 (C5), 124.9 (C1), 124.1 (C8), 122.8 (C2), 122.6 (C8a), 120.3 (C9a), 114.2 (C4), 53.4 ($N_{py}\underline{CH_2}CH_2$), 51.4 ($COCH_2\underline{CH_2}$), 36.3 ($CO\underline{CH_2}CH_2$), 23.2 ppm ($N_{py}CH_2\underline{CH_2}$). HRMS (ESI): m/z calcd for $C_{20}H_{20}ClN_3O_2+H^+$: 354.13677 [M+H]$^+$; found 354.13674.

3-{[3-(Benzylamino)propanoyl]amino}-9-(benzylamino)acridine ***(17k)***

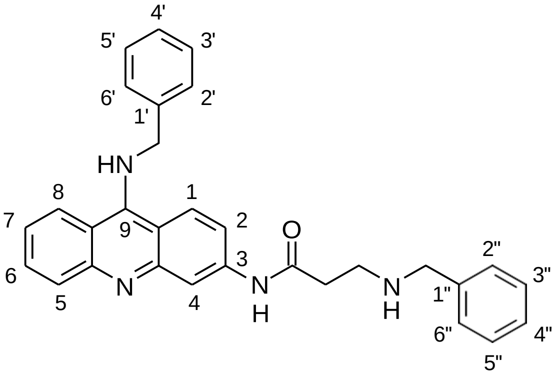

Yellow semisolid oil; weight 0.015 g; R_f = 0.26 (Et_2NH/Ethylacetate 1:3); ^{1}H NMR (600 MHz, Metanol–d_4): δ = 8.25 (d, J = 2.2 Hz, 1H; H4), 8.18–8.16 (m, 1H; H8), 8.09 (d, J = 9.4 Hz, 1H; H1), 7.80 (d, J = 7.5 Hz, 1H; H5), 7.62–7.59 (m, 1H; H6), 7.39–7.35 (m, 2H; H2´, H6´), 7.35–7.27 (m, 7H; H2, H3´, H5´, H2´´, H3´´, H5´´, H6´´), 7.26–7.21 (m, 3H; H7, H4´, H4´´), 4.98 (s, 2H; $NCH_2Ph´$), 3.77 (s, 2H; $NCH_2Ph´´$), 2.93 (t, J = 6.7 Hz, 2H; $COCH_2\underline{CH_2}$), 2.64 ppm (t, J = 6.7 Hz, 2H; $CO\underline{CH_2}CH_2$); ^{13}C NMR (150 MHz, Metanol–d_4): δ = 173.4 (CO), 154.4 (C9), 150.1 (C4a), 149.2 (C10a), 142.2 (C3), 140.7 (C1´), 140.6 (C1´´), 132.0 (C6), {129.8, 129.5, 128.5, 128.2 (C3´,C4´,C5´,C3´´,C4´´,C5´´)}, 129.5 (C2´´,C6´´), 128.0 (C2´,C6´), 127.0 (C5), 126.2 (C1), 124.9 (C8), 123.4 (C7), 118.0(C2), 116.9 (C8a), 114.4 (C4), 113.6 (C9a), 54.3 ($NCH_2Ph´´$), 54.0 ($NCH_2Ph´$), 45.7 ($COCH_2\underline{CH_2}$), 37.3 ppm ($CO\underline{CH_2}CH_2$). HRMS (ESI): m/z calcd for $C_{28}H_{31}N_5O+H^+$: 461.2336 [M+H]$^+$; found 461.2319.

CHEMINFORMATICS

Normal mode analysis

The 3D Viewer (ver. 11.01) and ChemSketch (ver. 11.02) components of the ACD Labs software package were used to build the models of acridine derivatives **17a**–**17j** [2]. The geometry of these derivatives was optimized at the semiempiric level of theory using the *PM7* method within the MOPAC2016 software package [3]. The optimization of acridine derivatives **17a**–**17j** was controlled using the commands: *LET*; *DDMIN* = 0; *GNORM* = 0.1; *AUX*; *EPS* = 78; *EF*; *XYZ*. The post-processing normal modes analysis was controlled using the keywords: *FORCE*; *THERMO*; *PRECISE*; *EPS* = 78. The results of the computational studies were visualized with the options provided in the Gabedit software package [4].

Calculation of ΔS° value

Changes in the standard entropy, ΔS°, of acridine derivatives **17a**–**17j** was calculated using the value of the standard entropy, S° (eq. S1), at a temperature of 300 and 340 degrees of Kelvin. The values of the standard entropy are listed in the Mopac output file.

$$\Delta S° = S°_{340K} - S°_{300K} \quad \text{(eq. S1)}$$

Calculation of LogP value

Log$P$ descriptor calculations were performed using the web-based platform Online Chemical Database with Modelling Environment (http://www.ochem.eu.) in order to obtain the log$P$ value of acridine derivatives **17a**–**17j** [5–9].

DNA BINDING EXPERIMENTS

A stock solution of *ct*DNA was prepared by dissolving an appropriate amount of *ct*DNA fibers in a Tris-HCl buffer (pH 7.4). The concentration of the prepared *ct*DNA was determined according to the Lambert-Beer law using an extinction coefficient for the base pair ε_{260nm} = 12 824 $M^{-1}\cdot cm^{-1}$. Stock solutions of all derivatives at a concentration of 30 mM were prepared in DMSO by dissolving powder forms and the resulting solutions were stored in dark conditions at -20°C. Amsacrine (AMS) and ethidium bromide (EtBr) solutions were also prepared from powder forms in DMSO (AMS 30 mM and EtBr 20 mM stock solution) except in the case of Hoechst 33258 which was used as a commercial solution in water (1mg/mL). Working solutions were prepared by dilution with DMSO with the exception of Hoechst which was diluted with deionized water. Concentrations of the working solutions of EtBr and Hoechst 33258 (Hoechst) were determined according to the appropriate extinction coefficients (EtBr: $\varepsilon_{480\ nm}$ = 5 680 $M^{-1}.cm^{-1}$; Hoechst: $\varepsilon_{345,5\ nm}$ = 46 000 $M^{-1}.cm^{-1}$). The stability of the derivatives was examined in 10 mM Tris-HCl buffer (pH 7.4) by UV-Vis spectroscopy at a time range of 0, 1 and 27 hours. Selected derivatives were divided into two testing groups, the first of which consisted of derivatives **17e**–**17i** and the second of derivatives **17a**–**17d**.

The absorption spectra of the free acridine derivatives and their complexes with *ct*DNA were measured using a Specord S300 UV-Vis spectrophotometer in a 10 mM Tris-HCl buffer (pH 7.4) at room temperature in a 100-QS quartz cuvette (1 cm path length). The concentration of *ct*DNA in the solution was gradually increased until saturation state was reached. The final concentrations of added DNA were: **17a,d**: 33 µM, **17b**: 31.5 µM, **17c**: 17.3 µM, **17e,f**: 31.4 µM, **17g**: 32.8 µM, **17h**: 27.4 µM, **17i**: 24.6 µM, **AMS**: 217.7 µM, **EtBr**: 133.4 µM, **Hoechst**: 8.6 µM. The concentration of samples were 16 µM for the studied compounds, 6.4 µM for Hoechst, 32 µM for AMS and 46.8 µM for EtBr. Absorption spectra were scanned at a wavelength range of 230–600 nm. The measured UV-Vis data for derivatives **17a**–**17i** were processed graphically using GraphPad Prism 6 software.

Binding constants K_B were calculated using the Benesi-Hildebrand equation (eq. S2) by plotting the obtained absorption titration values into a 1/C[*ct*DNA] vs. $A_0/(A-A_0)$ graph in Microsoft Office Excel. The displayed results are the averages of at least three independent measurements (with the exception of derivative **17d**).

$$\frac{A_0}{A_0-A} = \frac{\varepsilon_L}{\varepsilon_{ctDNA+L}-\varepsilon_L} + \frac{\varepsilon_L}{\varepsilon_{ctDNA+L}-\varepsilon_L} \times \frac{1}{K_B \times C_{[ctDNA]}} \quad \text{(eq. S2)}$$

where A_0 is the absorption of the derivative alone, A is the absorption of the derivative after the addition of *ct*DNA, ε_L is the extinction coefficient of the derivative, $\varepsilon_{ctDNA+L}$ is the extinction coefficient of the derivative-*ct*DNA complex, K_B is the binding constant and $C_{[ctDNA]}$ is the concentration of added *ct*DNA [10].

T_m measurements was carried out on a Jasco J-810 spectropolarimeter in a 1 mm quartz cuvette 100-QS in a BPES buffer (6 mM Na_2HPO_4, 2 mM NaH_2PO_4, 1 mM Na_2EDTA, 35 mM NaCl, pH 7.1). The concentration of *ct*DNA was 145 µM, the concentration of the ligands was 20 µM with the exception of Hoechst in which the concentration was 7 µM. Solutions of the complexes of *ct*DNA and derivatives were incubated 30 min before measurements at room temperature. Measurements were carried out in a temperature range of 50–100 °C at 260 nm with a heating rate of 1°C/min. Absorption thermal denaturation curves were processed using GraphPad Prism 6 and the results were used to determine the melting temperatures.

The CD spectra measurements were conducted using a J-810 Jasco spectropolarimeter in a quartz cuvette (1 cm path length). CD spectra of *ct*DNA (28 µM for derivatives **17e-17i**, 29 µM for derivatives **17a-17d**) in the absence or in the presence of derivatives **17a–17i** (0–16 µM) and reference molecules EtBr and AMS (0–16 µM) and Hoechst (0–1.3 µM) were recorded in a 10 mM Tris-HCl buffer (pH 7.4) in a wavelength range of 230–600 nm with a scan rate of 200 nm/min at room temperature. The results are presented as the mean of at least three independent measurements and the obtained data was processed using GraphPad Prism 6.

*h*TOPOISOMERASE I AND IIα EXPERIMENTS

Nuclease activity

The nuclease activity of selected molecules was studied on isolated plasmid pUC19 according to the adjusted standard protocol. Mixtures of pUC19 in TE buffer and the studied derivatives in DMSO were prepared at three concentrations (10 µM, 50 µM and 100 µM) and incubated at 37°C for 18 h in a 10 mM Tris-HCl buffer (pH 7.4). After the incubation, a solution of bromophenol blue and xylene violet was added and samples were purged with a chloroform:isoamyl alcohol solution (24:1) and subjected on a 1.5 % v/v agarose gel. Electrophoresis was run for 3 hours at 40 V in 1 × TAE (40 mM Tris, 20 mM acetic acid glacial, 1 mM EDTA), stained with EtBr and destained in deionized water. The electrophoretic record was visualized using UV light, photographed using a SYNGEN system and processed using GeneSnap software.

Relaxation assay for human Topoisomerase I

The impact of the studied derivatives on the relaxation ability of *h*Topo I (Inspiralis) was studied with plasmid pBR322 (Inspiralis). A solution consisting of plasmid (0.5 µg per sample) in a TE buffer (10 mM Tris-HCl (pH 7.5), 1 mM EDTA), diluted with *h*Topo I (0.8 U per sample for **17e–17i** and 1.0 U per sample for **17a–17d**) in a dilution buffer (10 mM Tris-HCl (pH 7.5), 1 mM DTT, 1 mM EDTA, 50% v/v glycerol, 50 µg/mL albumin, supplied as 1 × stock) and the studied derivatives was prepared in three concentrations (1µM, 2.5 µM and 5 µM (**17e–17i**) and 1µM, 5 µM and 10 µM (**17a–17d**), respectively). The solution was incubated at 37°C for 30 min in 1 × concentrated assay buffer (20 mM Tris-HCl (pH 7.5), 200 mM NaCl, 0.25 mM EDTA, 5% glycerol, 50 µg/mL albumin supplied as 10 × stock) with deionized water. After incubation, the reaction was stopped with STEB (40% v/v sucrose, 100 mM Tris-HCl (pH 8.0), 0.5 g/dm^3 bromophenol blue, 1 mM EDTA (**17e-17i**) or 10 mM EDTA (**17a–17d**)). The samples were purified with chloroform:isoamyl alcohol (24:1) and the upper layer of the samples was subsequently subjected on 1% v/v agarose gel. Electrophoresis was run for 15 min at 20 V to allow the subjected samples to penetrate the gel and then for 5 h at 30 V (**17e–17i**) resp. 4 h at 35 V (**17a–17d**) in 1xTAE buffer (40 mM Tris, 20 mM acetic acid glacial, 1 mM EDTA). Agarose gel was stained with EtBr solution and destained with deionized water. The electrophoretic results were visualized using UV light, photographed using a SYNGEN system and processed using GeneSnap software.

Unwinding assay for wheat germ *Topoisomerase I*

Wheat germ Topo I (wgTopo I) unwinding assays were carried out according to the Inspiralis protocol with supercoiled and relaxed pBR322 plasmid (0.3 µg per sample) in a TE buffer (10 mM Tris-HCl (pH 7.5), 1 mM EDTA) and an appropriate amount of diluted wgTopo I (0.8 U per sample) in a dilution buffer (50 mM Tris-HCl (pH 7.9), 500 mM NaCl, 1 mM DTT, 1 mM EDTA, 50% v/v glycerol, supplied as 1 × stock). Mixtures of plasmid, wgTopo I and studied derivatives **17d** and **17e** were prepared at 8 concentrations (0.1 µM, 0.5 µM, 1 µM, 2.5 µM, 5 µM, 10 µM, 50 µM and 100 µM) and incubated in a concentrated assay buffer (50 mM Tris-HCl (pH 7.9), 50 mM NaCl, 1 mM EDTA, 1 mM DTT, 20% v/v glycerol, supplied as 2 × stock) at 37°C for 30 min. After incubation, the reaction was stopped with STEB (40% v/v sucrose, 100 mM Tris-HCl (pH 8.0), 1 mM EDTA, 0.5 g/dm^3 bromophenol blue). The samples were purified with chloroform:isoamyl alcohol (24:1) and the upper layer of samples were subjected on 1% v/v agarose gel. Electrophoresis was run for 15 min at 20 V to allow the subjected samples to penetrate the gel and then for 2.5 hours at 57 V in a 1 × TAE buffer (40 mM Tris, 20 mM acetic acid glacial, 1 mM EDTA). Agarose gel was stained with EtBr solution and destained in distilled water. The electrophoretic results were visualized using UV light, photographed using a SYNGEN system and processed using GeneSnap software.

Decatenation assay for human Topoisomerase IIa

*h*Topo IIα decatenation assays were carried out according to the Inspiralis protocol using kinetoplast DNA (*k*DNA, 200 ng per sample) in a TE buffer (10 mM Tris-HCl (pH 8.0), 1 mM EDTA) and an appropriate amount of diluted *h*Topo IIα enzyme (1.2 U per sample) in a dilution buffer (50 mM Tris-HCl (pH 7.5), 100 mM NaCl, 1 mM DTT, 0.5 mM EDTA, 50% v/v glycerol, 50 µg/mL albumin, supplied as 1 × stock). Mixtures of *k*DNA, *h*Topo IIα and the studied derivatives were prepared at three concentrations (10 µM, 50 µM and 100 µM)

and incubated in a concentrated assay buffer (50 mM Tris-HCl (pH 7.5), 125 mM NaCl, 10 mM $MgCl_2$, 5 mM DTT, 100 μg/mL albumin, supplied as 10 × stock) with ATP (final concentration 1 mM) at 37°C for 30 min. After incubation, the reaction was stopped with STEB (40% v/v sucrose, 100 mM Tris-HCl (pH 8.0), 1 mM EDTA, 0.5 g/dm^3 bromophenol blue). The samples were purified with chloroform:isoamyl alcohol (24:1) and the upper layer of the samples were subjected on 1% v/v agarose gel. Electrophoresis was run for 15 min at 20 V to allow the subjected samples to penetrate the gel and then for 4 hours at 35 V in a 1 × TAE buffer (40 mM Tris, 20 mM acetic acid glacial, 10 mM EDTA). Agarose gel was stained with EtBr solution and destained in distilled water. The electrophoretic results were visualized using UV light, photographed using a SYNGEN system and processed using GeneSnap software.

SCREENING OF ANTICANCER ACTIVITY-NCI-60 PANELS

The anticancer activity of acridines **17a**–**17j** was tested against NCI-60 panels consisting of sixty human cancer cell lines. Derivatives **17a**–**17j** were initially used at a single dose of 10 μM to identify the individual derivatives with the highest level of growth inhibition. Acridine derivatives **17a**, **17b** and **17e**–**17h** were found to have exhibited significant growth inhibition in the One-Dose Screen, and these derivatives were further evaluated against NCI-60 panels at five concentration levels to determine the growth inhibition concentration, GI_{50}. The GI_{50} values for doxorubicin, fluorouracil and cisplatin were downloaded from the NCI data repository (https://dtp.cancer.gov/databases_tools/data_search.htm). The screening protocol is described on the web page: https://dtp.cancer.gov/discovery_development/nci-60/methodology.htm

MOLECULAR DOCKING

Ligand preparation

Molecular models of acridine derivatives **17a**–**17j** with the pyrrolidine moiety in a form of pyrrolidinium salt were simulated in digitized form using the building options in ACD/ChemSketch software [2]. The models were built as 3D structures and saved as mopac input files using the ACD/3D Viewer [2]. MOPAC2016 was used to optimize the ligand geometry [3].

Receptor preparation

Chimera software was used to extract the coordinates of all non-standard residues, water and ions from the cleavage complex of the x-ray [11,12]. The crystal structure of the *h*Topo I (pdb ID: 1T8I), of the human topoisomerase IIα (pdb ID: 5GWK) and of the *h*Topo IIβ (pdb ID: 4G0U) were chosen as receptors [13–15].

Charge assignment

MGL TOOLS 1.5.6 was used to assign Gasteiger partial atomic charges for ligands, for nucleotides and for proteins [16,17].

Docking run

Docking simulations were carried out using Autodock ver. 4.2 software, while MGL TOOLS 1.5.6 was used to prepare the input files [18,19]. United atom representations were used for all components of the used complexes.

The grid for the energy of the complex *h*Topo I – DNA (pdb id: 1T8I) was set at xyz-coordinates 22.029, 2.654, 28.295. The grid for the energy of the complex *h*Topo IIα – DNA (pdb id: 5GWK) was set at xyz-coordinates 30.876, -23.214, -58.150. The grid for the energy of the complex *h*Topo IIβ – DNA (pdb id: 4G0U) was set at xyz-coordinates 32.327, 91.980, 47.080. The grid for the energy of all complexes had xyz-dimensions of 80 × points; 80× points; 80× points and a spacing of 0.375 Å.

Docking runs were performed using a Larmarckian genetic algorithm. Docking began with a population of random ligand conformations in a random orientation and at a random translation. Each docking experiment was derived from 200 different runs which were set to terminate after a maximum of 5 × 10^6 energy evaluations or

27×10^3 generations, yielding 200 docked conformations. The population size was set to 300. For all other parameters, default values were used.

A total of 200 individual docking simulations were run for each of the studied compounds, and the obtained results were analyzed using AutoDockTools-1.5.6 software. The analysis was based on a visual inspection of the resulting docking poses for compounds **17a**–**17j** within their complex with the enzyme. An additional clustering analysis of the ligands' docking poses was also performed using the "RECLUSTER" (3.0 rms tolerance) option within the "ANALYZE" option of the AutoDockTools-1.5.6 software. The five clusters with the lowest binding energy were identified and examined (Tables S5 – S7, Figures S11 – S19). From this set, the docking pose with the lowest binding energy from the highest populated cluster was assumed to represent the putative binding mode of the ligand in the ternary DNA cleavage enzyme complex. This rescoring protocol was applied to all docking studies.

Figure preparation

All figures showing ligand docking poses presented here and in the supplementary material were prepared using Chimera software [11,12].

Table S1. Selected absorption titration data of 3,9-disubstituted acridine derivatives **17a**–**17i** and reference molecules **EtBr**, **AMS** and **Hoechst**

Compound	Hypochromicity (%)	λ_{max} (nm)		$\Delta\lambda_{max}$ (nm)	K_B x 10^4 (dm^3.mol^{-1})	T_m (°C)	Δs (°C)
		Free	Bound				
**17a**	23.30 ± 0.07	354	357	3	4.32 ± 1.97	87	10
**17b**	30.00 ± 0.62	348	352	4	4.65 ± 1.02	91	14
**17c**	28.15 ± 4.64	356	361	5	9.03 ± 1.94	-	-
**17d**	25.49 ± 1.03	354	357	3	3.41 ± 0.38	88	11
**17e**	36.50 ± 1.99	346	352	6	2.81 ± 1.08	91	14
**17f**	37.05 ± 1.34	346	353	7	5.53 ± 2.05	90	13
**17g**	34.02 ± 0.73	347	353	6	4.28 ± 1.76	91	14
**17h**	32.86 ± 1.89	346	351	5	4.59 ± 0.56	-	-
**17i**	34.46 ± 4.36	345	350	5	6.28 ± 3.50	-	-
**EtBr**	57.03 ± 0.25	477	520	43	0.39 ± 0.05	84	7
**AMS**	39.49 ± 0.69	436	443	7	0.66 ± 0.19	78	1
**Hoechst**	45.54 ± 0.23	339	347	8	24.93 ± 1.70	83	6

Table S2. Physicochemical properties of 3,9-disubstituted acridine derivatives **17a**–**17j**.

Compound	Log$P$ ^{a}	Charge ^{b}	$\Delta S°_{300K}$ ^{c}	$\Delta S°_{340K}$ ^{d}	$\Delta S°$ ^{e}	K_B ^{f}
Number		δ	cal/K.mol	cal/K.mol	cal/K.mol	M^{-1}
**17a**	5.07	0.270	189.1	203.4	14.3	4.32×10^4
**17b**	4.97	0.313	215.4	231.8	16.4	4.65×10^4
**17c**	6.08	0.254	199.2	214.0	14.9	9.03×10^4
**17d**	5.18	0.264	193.9	208.6	14.7	3.41×10^4
**17e**	4.72	0.323	202.4	217.3	14.9	2.81×10^4

**17f**	5.11	0.330	206.3	222.0	15.7	5.53×10^{4}
**17g**	5.09	0.327	203.4	218.6	15.3	4.28×10^{4}
**17h**	5.09	0.344	207.9	223.5	15.6	4.59×10^{4}
**17i**	5.40	0.350	217.7	233.9	16.2	6.28×10^{4}
**17j**	5.86	0.349	219.5	236.4	16.9	8.80×10^{4} [g]

[a] Octanol – water partition coefficient as predicted by the Online Chemical Database (ochem.eu).
[b] Value of the sum of partial charges of atoms within a substituent at position 9 of the acridine scaffold.
[c] Standard entropy for acridine derivatives **17a**–**17j** at a temperature of 300K.
[d] Standard entropy for acridine derivatives **17a**–**17j** at a temperature of 340K.
[e] Gap between the standard entropy for acridine derivatives **17a**–**17j** $\Delta S° = S°_{340K} - S°_{300K}$.
[f] Value of the binding constant K_B for the complex of *ct*DNA and acridine derivatives **17a**–**17j**.
[g] Value of K_B calculated by an extrapolation of the linear correlation between K_B and Log*P* for derivatives **17e**, **17h**, **17i**.

Table S3. Partial atomic charges, in accordance with Figure S8, on atoms within the substituent at location 9 of the acridine scaffold of derivatives **17a**–**17j** obtained by PM7 method.

	**PAH**	**PNN**	**PAB**	**PAF**	**BAH**	**BAM**	**BAF**	**PEA**	**PPA**	**PBA**
**Atom**	**17a**	**17b**	**17c**	**17d**	**17e**	**17f**	**17g**	**17h**	**17i**	**17j**
**N**	-0.445	-0.397	-0.458	-0.440	-0.413	-0.410	-0.417	-0.407	-0.404	-0.408
**NH**	0.360	0.356	0.361	0.361	0.341	0.343	0.345	0.348	0.348	0.351
**H1**					0.186	0.184	0.185	0.168	0.173	0.170
**H2**					0.182	0.180	0.182	0.175	0.171	0.170
**C1**					-0.101	-0.094	-0.093	-0.080	-0.092	-0.094
**H3**								0.179	0.159	0.159
**H4**								0.173	0.164	0.162
**C2**								-0.320	-0.285	-0.295
**H5**									0.163	0.149
**H6**									0.167	0.152
**C3**									-0.296	-0.259
**H7**										0.162
**H8**										0.159
**C4**										-0.305
**C5**	0.148	0.030	0.176	0.119	-0.059	-0.090	-0.093	0.004	0.012	0.018
**C6**	-0.195	-0.102	-0.193	-0.146	-0.143	-0.121	-0.089	-0.184	-0.193	-0.197
**C7**	-0.121	-0.279	-0.114	-0.191	-0.163	-0.213	-0.235	-0.152	-0.150	-0.148
**C8**	-0.168	0.218	-0.055	0.177	-0.146	0.061	0.203	-0.173	-0.179	-0.181
**C9**	-0.128	-0.266	-0.121	-0.198	-0.163	-0.214	-0.235	-0.150	-0.150	-0.147
**C10**	-0.193	-0.102	-0.186	-0.141	-0.154	-0.131	-0.099	-0.182	-0.195	-0.199
**H9**	0.179	0.182	0.189	0.189	0.170	0.171	0.181	0.165	0.168	0.165
**H10**	0.170	0.178	0.189	0.194	0.164	0.170	0.191	0.162	0.163	0.163
**X11**	0.167	-0.403	-0.081	-0.188	0.164	-0.476	-0.193	0.163	0.164	0.164
**H12**	0.167	0.181	0.186	0.192	0.167	0.167	0.189	0.164	0.162	0.162
**H13**	0.182	0.180	0.192	0.191	0.172	0.170	0.179	0.169	0.164	0.163
**H14**		0.151				0.174				
**H15**		0.161				0.174				

H16		0.166				0.167				
**H17**		0.151								
**H18**		0.162								
**H19**		0.162								
**C11**		-0.276								
**C12**		-0.272								
**Suma**	**0.123**	**0.181**	**0.085**	**0.119**	**0.204**	**0.212**	**0.201**	**0.222**	**0.234**	**0.236**

Table S4. The geometry of the optimized structure of compounds **17a**–**17j** in a mopac cartesian coordinate style obtained by PM7 method.

PAH/17a	PNN/17b
C 0.04133200 +1 0.03587747 +1 0.03879739 +1	C 0.01919225 +1 0.00703386 +1 0.06949226 +1
C 1.46559116 +1 0.01677668 +1 -0.04895630 +1	C 1.44228425 +1 -0.00494266 +1 -0.02731497 +1
C -0.60534363 +1 1.28949195 +1 0.19858334 +1	C -0.63532230 +1 1.25427409 +1 0.22366067 +1
C 2.19442104 +1 1.23605174 +1 0.05322755 +1	C 2.16380707 +1 1.21703693 +1 0.08597783 +1
C 0.09933483 +1 2.45855768 +1 0.27910285 +1	C 0.06252610 +1 2.42888245 +1 0.30895143 +1
C 1.52370233 +1 2.43415639 +1 0.20862880 +1	C 1.48579739 +1 2.41116336 +1 0.24923001 +1
C -0.65543726 +1 -1.21710671 +1 -0.00147260 +1	C -0.67195423 +1 -1.25433051 +1 0.03990914 +1
C 0.08071431 +1 -2.38104185 +1 -0.39125354 +1	C 0.06498827 +1 -2.40418516 +1 -0.40204593 +1
N 2.17595127 +1 -1.12706665 +1 -0.21698804 +1	N 2.15746572 +1 -1.14259622 +1 -0.21771352 +1
C 1.50270978 +1 -2.28828566 +1 -0.41862486 +1	C 1.48512120 +1 -2.30253133 +1 -0.44376281 +1
C -0.51395105 +1 -3.60446288 +1 -0.80254769 +1	C -0.52879748 +1 -3.61594913 +1 -0.83892757 +1
C 0.24920487 +1 -4.70150301 +1 -1.09801050 +1	C 0.23610216 +1 -4.70215397 +1 -1.17575510 +1
C 2.28288677 +1 -3.46041577 +1 -0.68646744 +1	C 2.26679219 +1 -3.46104314 +1 -0.75369030 +1
C 1.66448384 +1 -4.63557311 +1 -1.00595456 +1	C 1.64976875 +1 -4.63111787 +1 -1.10000276 +1
N -1.97030082 +1 -1.23443464 +1 0.34005165 +1	N -1.95068002 +1 -1.28857738 +1 0.47149350 +1
N 2.29877831 +1 3.60495452 +1 0.32874511 +1	N 2.25828603 +1 3.58255113 +1 0.38362821 +1
C 1.83193773 +1 4.89504180 +1 0.13201718 +1	C 1.78771147 +1 4.87633951 +1 0.22646419 +1
O 0.68618744 +1 5.13342840 +1 -0.19892894 +1	O 0.63704025 +1 5.12403423 +1 -0.07984839 +1
C 2.85237230 +1 5.98411567 +1 0.35355879 +1	C 2.81236300 +1 5.95975583 +1 0.45841184 +1
C 2.20726129 +1 7.34216355 +1 0.08815684 +1	C 2.15718768 +1 7.32510319 +1 0.26513112 +1
N 3.14049703 +1 8.45487989 +1 0.48957052 +1	N 3.10054341 +1 8.42385493 +1 0.68136393 +1
C 3.58170131 +1 10.81920721 +1 0.15834409 +1	C 3.52538056 +1 10.80156068 +1 0.43661295 +1
C 2.45433225 +1 9.80825815 +1 0.39034755 +1	C 2.40844763 +1 9.77767608 +1 0.66264779 +1
C 4.78252981 +1 10.02146316 +1 -0.38166461 +1	C 4.70947489 +1 10.03160946 +1 -0.17590203 +1
C 4.35354521 +1 8.55036169 +1 -0.42101563 +1	C 4.28185560 +1 8.56229951 +1 -0.26471769 +1
H -1.70065623 +1 1.33148288 +1 0.25272596 +1	H -1.73145310 +1 1.29007773 +1 0.26730030 +1
H 3.28230304 +1 1.19375146 +1 -0.00098300 +1	H 3.25163875 +1 1.18149457 +1 0.03390338 +1
H -0.42190572 +1 3.40693957 +1 0.41005713 +1	H -0.46596029 +1 3.37327380 +1 0.43406998 +1
H -1.59991897 +1 -3.66030745 +1 -0.91444566 +1	H -1.61605410 +1 -3.67598911 +1 -0.93640296 +1
H -0.21532023 +1 -5.63544547 +1 -1.41238300 +1	H -0.22973606 +1 -5.62846732 +1 -1.50898373 +1
H 3.36796585 +1 -3.37667894 +1 -0.63840100 +1	H 3.35166067 +1 -3.37431844 +1 -0.71724449 +1
H 2.24643372 +1 -5.53797509 +1 -1.21170910 +1	H 2.23508589 +1 -5.52299701 +1 -1.33857632 +1
H -2.48380782 +1 -0.34542329 +1 0.42789670 +1	H -2.45480243 +1 -0.40957360 +1 0.65306661 +1
H 3.29984876 +1 3.47280262 +1 0.54017401 +1	H 3.26194225 +1 3.44839432 +1 0.58250174 +1
H 3.74181785 +1 5.82243555 +1 -0.30191899 +1	H 3.68074938 +1 5.82525089 +1 -0.23069669 +1
H 3.25347116 +1 5.92479069 +1 1.39688601 +1	H 3.24632489 +1 5.86274268 +1 1.48547573 +1
H 1.25604185 +1 7.44643273 +1 0.67290377 +1	H 1.22387319 +1 7.40408173 +1 0.88176446 +1
H 1.92111060 +1 7.44936297 +1 -0.98673860 +1	H 1.83865113 +1 7.47309607 +1 -0.79563108 +1
H 3.84665800 +1 11.34913752 +1 1.09327075 +1	H 3.81997438 +1 11.29257075 +1 1.38382349 +1
H 3.26776811 +1 11.60858823 +1 -0.55064468 +1	H 3.18654380 +1 11.61909813 +1 -0.22751652 +1
H 1.88024275 +1 10.00613995 +1 1.32149245 +1	H 1.86526320 +1 9.93393777 +1 1.61995616 +1
H 1.72615557 +1 9.80964440 +1 -0.45069470 +1	H 1.65265341 +1 9.81206904 +1 -0.15287562 +1
H 5.67329712 +1 10.16493096 +1 0.25908397 +1	H 5.62032498 +1 10.15019660 +1 0.44124340 +1
H 5.08291437 +1 10.37252120 +1 -1.38717046 +1	H 4.97657411 +1 10.42665385 +1 -1.17443335 +1
H 4.08568204 +1 8.23280256 +1 -1.45331329 +1	H 3.97951185 +1 8.28906508 +1 -1.30016119 +1
H 5.14912164 +1 7.86297042 +1 -0.06305911 +1	H 5.09024092 +1 7.86247500 +1 0.03576676 +1
H 3.46319941 +1 8.30418898 +1 1.48291286 +1	H 3.45713787 +1 8.23305949 +1 1.65599160 +1
C -2.70340533 +1 -2.39469243 +1 0.72977055 +1	C -2.69457526 +1 -2.47346472 +1 0.75438786 +1
C -4.21408913 +1 -4.57581158 +1 1.54065862 +1	C -4.23029344 +1 -4.73156934 +1 1.37936038 +1
C -2.94895273 +1 -4.36471473 +1 2.08849171 +1	C -2.97874320 +1 -4.51558165 +1 1.99886416 +1
C -4.72532596 +1 -3.68938522 +1 0.59421063 +1	C -4.68737826 +1 -3.78628313 +1 0.43661459 +1
C -2.18591715 +1 -3.27165283 +1 1.69123285 +1	C -2.22329361 +1 -3.39553603 +1 1.69710177 +1
C -3.97608416 +1 -2.59107955 +1 0.18198296 +1	C -3.93018861 +1 -2.66660699 +1 0.12907785 +1
H -4.80585186 +1 -5.43613449 +1 1.85501255 +1	N -5.05406730 +1 -5.78865564 +1 1.79916651 +1
H -2.55468784 +1 -5.05810386 +1 2.83417558 +1	H -2.59312510 +1 -5.22913347 +1 2.72739355 +1
H -5.71777636 +1 -3.85597720 +1 0.17122852 +1	H -5.64376976 +1 -3.92397265 +1 -0.06683837 +1
H -1.19724405 +1 -3.10224303 +1 2.11922966 +1	H -1.25871175 +1 -3.23547748 +1 2.18657548 +1
H -4.37347635 +1 -1.89565234 +1 -0.55891232 +1	H -4.29609936 +1 -1.94140311 +1 -0.60344918 +1
	C -4.36744025 +1 -6.97367028 +1 2.34963496 +1
	C -6.15995512 +1 -6.16269471 +1 0.89764369 +1
	H -3.63053658 +1 -7.41157463 +1 1.65570356 +1
	H -5.10306193 +1 -7.75746292 +1 2.60245152 +1

	H -3.85105773 +1 -6.70833476 +1 3.28984416 +1
	H -5.82510668 +1 -6.43822804 +1 -0.11653297 +1
	H -6.87862491 +1 -5.32753248 +1 0.81634755 +1
	H -6.71672734 +1 -7.02109827 +1 1.31417620 +1
**PAB/17c**	**PAF/17d**
C 0.04547801 +1 -0.00884289 +1 0.03274554 +1	C 0.03576253 +1 0.02660935 +1 0.01260856 +1
C 1.47220793 +1 -0.01451513 +1 0.08807716 +1	C 1.46077076 +1 0.00784173 +1 -0.06469423 +1
C -0.61327006 +1 1.24157638 +1 -0.11835184 +1	C -0.61219149 +1 1.28144130 +1 0.15962383 +1
C 2.18807277 +1 1.21212329 +1 -0.01456704 +1	C 2.18838589 +1 1.22785529 +1 0.03529930 +1
C 0.07988048 +1 2.41564940 +1 -0.20991663 +1	C 0.09151567 +1 2.45093785 +1 0.23911893 +1
C 1.50641839 +1 2.40594503 +1 -0.15789345 +1	C 1.51671081 +1 2.42687574 +1 0.18044805 +1
C -0.63786877 +1 -1.26405836 +1 0.08979930 +1	C -0.65958106 +1 -1.22640424 +1 -0.02387451 +1
C 0.11854865 +1 -2.43182273 +1 0.41217321 +1	C 0.07932316 +1 -2.39360735 +1 -0.39705066 +1
N 2.19607426 +1 -1.15428024 +1 0.21888812 +1	N 2.17247261 +1 -1.13699987 +1 -0.21910081 +1
C 1.54044385 +1 -2.32696410 +1 0.40242995 +1	C 1.50135268 +1 -2.30015359 +1 -0.41539035 +1
C -0.45350545 +1 -3.67591142 +1 0.79995578 +1	C -0.51155906 +1 -3.62135164 +1 -0.80190235 +1
C 0.32747049 +1 -4.77370027 +1 1.03435898 +1	C 0.25442983 +1 -4.72058433 +1 -1.08039762 +1
C 2.33897379 +1 -3.50025518 +1 0.60974213 +1	C 2.28442186 +1 -3.47426603 +1 -0.66672604 +1
C 1.74033042 +1 -4.69094224 +1 0.90471998 +1	C 1.66918492 +1 -4.65282434 +1 -0.97899285 +1
N -1.98214193 +1 -1.28384685 +1 -0.15318676 +1	N -1.97815225 +1 -1.24148638 +1 0.30654145 +1
N 2.27543808 +1 3.57900683 +1 -0.27669000 +1	N 2.29185521 +1 3.59695389 +1 0.30091851 +1
C 1.79292658 +1 4.87584952 +1 -0.18696848 +1	C 1.82551381 +1 4.88924336 +1 0.11638474 +1
O 0.62434478 +1 5.13047140 +1 0.03144853 +1	O 0.67720868 +1 5.13273515 +1 -0.20134895 +1
C 2.83134305 +1 5.95466323 +1 -0.37724669 +1	C 2.85177594 +1 5.97442578 +1 0.33091832 +1
C 2.16251736 +1 7.32217217 +1 -0.25953712 +1	C 2.18966045 +1 7.33759505 +1 0.14533036 +1
N 3.14525527 +1 8.41880298 +1 -0.57740922 +1	N 3.15165427 +1 8.44147849 +1 0.50039416 +1
C 3.54494073 +1 10.79692002 +1 -0.29799420 +1	C 3.55863842 +1 10.81723775 +1 0.21602258 +1
C 2.45586682 +1 9.77320594 +1 -0.63381686 +1	C 2.45653666 +1 9.79397810 +1 0.50740573 +1
C 4.65979100 +1 10.02791277 +1 0.43407193 +1	C 4.70617893 +1 10.04531055 +1 -0.46022769 +1
C 4.22244087 +1 8.55965158 +1 0.48555686 +1	C 4.27997247 +1 8.57360881 +1 -0.50938046 +1
H -1.70955073 +1 1.27384117 +1 -0.16016821 +1	H -1.70793019 +1 1.32325987 +1 0.20439655 +1
H 3.27711182 +1 1.17845222 +1 0.02650127 +1	H 3.27678815 +1 1.18526052 +1 -0.01138810 +1
H -0.45402544 +1 3.35848073 +1 -0.33294236 +1	H -0.43166355 +1 3.39973315 +1 0.35991246 +1
H -1.53501052 +1 -3.74402777 +1 0.94377855 +1	H -1.59616099 +1 -3.67795369 +1 -0.92487618 +1
H -0.11839876 +1 -5.72322704 +1 1.33016044 +1	H -0.20705857 +1 -5.65816197 +1 -1.38947951 +1
H 3.42168952 +1 -3.40273981 +1 0.53528563 +1	H 3.36922067 +1 -3.38883796 +1 -0.61188838 +1
H 2.33523341 +1 -5.59469627 +1 1.06216906 +1	H 2.25333399 +1 -5.55684159 +1 -1.17180307 +1
H -2.51119472 +1 -0.39995487 +1 -0.12667576 +1	H -2.49282292 +1 -0.35263331 +1 0.38767157 +1
H 3.29120972 +1 3.44674623 +1 -0.41072508 +1	H 3.29456503 +1 3.46340181 +1 0.50631785 +1
H 3.65215567 +1 5.83764606 +1 0.37080250 +1	H 3.70810993 +1 5.83892927 +1 -0.37337373 +1
H 3.33167657 +1 5.83485989 +1 -1.37083328 +1	H 3.30365066 +1 5.88146275 +1 1.35025706 +1
H 1.30005214 +1 7.39837904 +1 -0.97224440 +1	H 1.28515564 +1 7.42676685 +1 0.80193981 +1
H 1.73365449 +1 7.47469709 +1 0.76137384 +1	H 1.82336470 +1 7.46915163 +1 -0.90222306 +1
H 3.93489186 +1 11.28536417 +1 -1.21156002 +1	H 3.90615300 +1 11.31059971 +1 1.14395132 +1
H 3.14072632 +1 11.61644421 +1 0.32604911 +1	H 3.18274729 +1 11.63310057 +1 -0.43003784 +1
H 2.01275065 +1 9.92616140 +1 -1.64157267 +1	H 1.96319923 +1 9.95663989 +1 1.49033688 +1
H 1.62064531 +1 9.81061296 +1 0.10053286 +1	H 1.65948258 +1 9.82077346 +1 -0.26811428 +1
H 5.62832228 +1 10.14212580 +1 -0.08868462 +1	H 5.65256489 +1 10.17278025 +1 0.09894465 +1
H 4.82581218 +1 10.42688681 +1 1.45282282 +1	H 4.91025730 +1 10.43160612 +1 -1.47696401 +1
H 3.81353218 +1 8.29172446 +1 1.48479703 +1	H 3.92362364 +1 8.28749458 +1 -1.52393041 +1
H 5.05648765 +1 7.85697534 +1 0.27405521 +1	H 5.10617791 +1 7.87987678 +1 -0.24536358 +1
H 3.60061157 +1 8.22585399 +1 -1.50852619 +1	H 3.56057158 +1 8.26004288 +1 1.45569529 +1
C -2.70687702 +1 -2.41136729 +1 -0.62390115 +1	C -2.70715258 +1 -2.39928799 +1 0.70493491 +1
C -4.20339938 +1 -4.51787142 +1 -1.56964616 +1	C -4.19233218 +1 -4.55076081 +1 1.52231424 +1
C -2.93138032 +1 -4.31278159 +1 -2.09169249 +1	C -2.94408523 +1 -4.34634260 +1 2.10988978 +1
C -4.75101488 +1 -3.69110961 +1 -0.59635400 +1	C -4.72504061 +1 -3.71008234 +1 0.54666904 +1
C -2.17517741 +1 -3.24603274 +1 -1.61756809 +1	C -2.19629376 +1 -3.25091809 +1 1.69536777 +1
C -3.99710686 +1 -2.62362833 +1 -0.11810793 +1	C -3.96859774 +1 -2.61810228 +1 0.13509128 +1
Br -5.23167115 +1 -5.98187753 +1 -2.21438592 +1	F -4.90904848 +1 -5.59637837 +1 1.91338885 +1
H -2.51458973 +1 -4.96440626 +1 -2.86515272 +1	H -2.55820529 +1 -5.02082948 +1 2.87793360 +1
H -5.75494339 +1 -3.85978890 +1 -0.19689471 +1	H -5.70859769 +1 -3.89610727 +1 0.10969825 +1
H -1.17550880 +1 -3.06236080 +1 -2.01810448 +1	H -1.21538509 +1 -3.05629560 +1 2.13737686 +1
H -4.40707275 +1 -1.95963919 +1 0.64733869 +1	H -4.35483251 +1 -1.93745730 +1 -0.62908520 +1
**BAH/17e**	**BAM/17f**
C 0.00059970 +1 0.03671426 +1 0.04204697 +1	C -0.02901304 +1 0.04391677 +1 0.06656419 +1
C 1.42047511 +1 0.02448840 +1 -0.08154055 +1	C 1.39269272 +1 0.03323303 +1 -0.03289596 +1
C -0.65466953 +1 1.28334223 +1 0.18119575 +1	C -0.68775675 +1 1.28791574 +1 0.21054988 +1
C 2.14198149 +1 1.24678468 +1 0.00674052 +1	C 2.11201392 +1 1.25460598 +1 0.08334534 +1
C 0.04279649 +1 2.46087191 +1 0.24058497 +1	C 0.00799248 +1 2.46509614 +1 0.29510719 +1
C 1.46436979 +1 2.44298581 +1 0.16812101 +1	C 1.43039845 +1 2.44810878 +1 0.24537551 +1
C -0.68806438 +1 -1.22985033 +1 0.06039845 +1	C -0.71707454 +1 -1.22355086 +1 0.05736499 +1
C 0.03350190 +1 -2.37748459 +1 -0.41507760 +1	C 0.01270658 +1 -2.36307641 +1 -0.42611171 +1
N 2.13201796 +1 -1.11765248 +1 -0.27384432 +1	N 2.10812654 +1 -1.10593994 +1 -0.22894106 +1
C 1.45345071 +1 -2.27507529 +1 -0.48721795 +1	C 1.43347834 +1 -2.25988311 +1 -0.47260247 +1
C -0.57097308 +1 -3.57707972 +1 -0.86676428 +1	C -0.58487406 +1 -3.55370157 +1 -0.90881393 +1
C 0.18613817 +1 -4.66106783 +1 -1.22990515 +1	C 0.17787274 +1 -4.63189726 +1 -1.27786768 +1
C 2.22681496 +1 -3.43382505 +1 -0.81852909 +1	C 2.21179914 +1 -3.41329923 +1 -0.81021254 +1
C 1.60076129 +1 -4.59750933 +1 -1.16965194 +1	C 1.59115260 +1 -4.57034788 +1 -1.19209681 +1
N -1.93335796 +1 -1.26384398 +1 0.56484150 +1	N -1.96716112 +1 -1.26756508 +1 0.54634337 +1
C -2.67383319 +1 -2.47613929 +1 0.95085713 +1	C -2.71078318 +1 -2.48670358 +1 0.90738622 +1
N 2.23914114 +1 3.61430586 +1 0.28625166 +1	N 2.20030875 +1 3.62035725 +1 0.38966610 +1
C 1.76444826 +1 4.90941407 +1 0.15189550 +1	C 1.73104379 +1 4.91237079 +1 0.21517165 +1
O 0.60828464 +1 5.15971392 +1 -0.13007938 +1	O 0.58858639 +1 5.15499374 +1 -0.12419342 +1
C 2.79242707 +1 5.99139409 +1 0.37655632 +1	C 2.74552437 +1 5.99955447 +1 0.47255137 +1
C 2.12134934 +1 7.35768754 +1 0.25809953 +1	C 2.09740251 +1 7.36178656 +1 0.23754032 +1
N 3.06384947 +1 8.44631031 +1 0.70166143 +1	N 3.01789725 +1 8.46632781 +1 0.68802171 +1

C 3.46786532 +1 10.83505723 +1 0.54714863 +1	C 3.45625457 +1 10.84085036 +1 0.43164394 +1
C 2.36186071 +1 9.79433367 +1 0.74901706 +1	C 2.32863923 +1 9.81967874 +1 0.61325350 +1
C 4.64807344 +1 10.09915726 +1 -0.11307578 +1	C 4.67403077 +1 10.06140355 +1 -0.09726302 +1
C 4.22769674 +1 8.63218097 +1 -0.25770865 +1	C 4.24819494 +1 8.59222363 +1 -0.19515436 +1
H -1.75010259 +1 1.31687825 +1 0.23259693 +1	H -1.78394498 +1 1.31990672 +1 0.24561785 +1
H 3.22912346 +1 1.21200613 +1 -0.06056084 +1	H 3.20013331 +1 1.22144699 +1 0.03511098 +1
H -0.48750098 +1 3.40575735 +1 0.35559859 +1	H -0.52333615 +1 3.40890370 +1 0.41350208 +1
H -1.65871751 +1 -3.62727732 +1 -0.96880151 +1	H -1.67092831 +1 -3.60080031 +1 -1.02944831 +1
H -0.28661229 +1 -5.57905092 +1 -1.57596703 +1	H -0.28925189 +1 -5.54330360 +1 -1.64793620 +1
H 3.31256303 +1 -3.35067906 +1 -0.79691729 +1	H 3.29702651 +1 -3.33154952 +1 -0.76872303 +1
H 2.17959572 +1 -5.48863629 +1 -1.42644967 +1	H 2.17402043 +1 -5.45728738 +1 -1.45416940 +1
H -2.40221710 +1 -0.38653226 +1 0.81845260 +1	H -2.44214679 +1 -0.39501541 +1 0.80610444 +1
H -1.97162959 +1 -3.32182173 +1 1.16071868 +1	H -2.01211054 +1 -3.34074329 +1 1.09202550 +1
H -3.33224765 +1 -2.80255181 +1 0.10405431 +1	H -3.37533843 +1 -2.78747123 +1 0.05639841 +1
H 3.24596382 +1 3.48003036 +1 0.46652295 +1	H 3.19800823 +1 3.48799822 +1 0.61467134 +1
H 3.62953491 +1 5.88559449 +1 -0.35477967 +1	H 3.64033632 +1 5.85812965 +1 -0.17986570 +1
H 3.27211728 +1 5.86379875 +1 1.37950655 +1	H 3.13838382 +1 5.91501217 +1 1.51722874 +1
H 1.20173921 +1 7.40078739 +1 0.89876641 +1	H 1.13654443 +1 7.44372980 +1 0.81018277 +1
H 1.77756816 +1 7.54873018 +1 -0.78764526 +1	H 1.82822073 +1 7.50015361 +1 -0.83798918 +1
H 3.77324585 +1 11.29043602 +1 1.50861999 +1	H 3.69600653 +1 11.35064929 +1 1.38433260 +1
H 3.11302743 +1 11.67552820 +1 -0.07886303 +1	H 3.15533693 +1 11.64468323 +1 -0.26662022 +1
H 1.83291021 +1 9.90747667 +1 1.72022948 +1	H 1.73569674 +1 9.98930938 +1 1.53806991 +1
H 1.59278271 +1 9.85582782 +1 -0.05235084 +1	H 1.61680240 +1 9.84208981 +1 -0.24133122 +1
H 5.56647157 +1 10.19747547 +1 0.49640948 +1	H 5.54492609 +1 10.18511865 +1 0.57420323 +1
H 4.89929220 +1 10.53737532 +1 -1.09756205 +1	H 5.00392693 +1 10.44407607 +1 -1.08166742 +1
H 3.90891679 +1 8.40133004 +1 -1.29847910 +1	H 4.00038210 +1 8.30971489 +1 -1.24252242 +1
H 5.04504328 +1 7.92526719 +1 -0.00131706 +1	H 5.03743507 +1 7.89348280 +1 0.15468656 +1
H 3.43873859 +1 8.21963200 +1 1.66164780 +1	H 3.32188649 +1 8.28919078 +1 1.68289402 +1
C -3.49564845 +1 -2.15703269 +1 2.17246477 +1	C -3.51809827 +1 -2.19198255 +1 2.14289496 +1
C -5.00932125 +1 -1.55674506 +1 4.43153734 +1	C -5.00505535 +1 -1.63098682 +1 4.43908776 +1
C -3.81886538 +1 -2.27037116 +1 4.56000708 +1	C -3.77599684 +1 -2.29158966 +1 4.53952041 +1
C -5.44276150 +1 -1.14341191 +1 3.17266541 +1	C -5.48403162 +1 -1.25418399 +1 3.18173128 +1
C -3.05930066 +1 -2.57014984 +1 3.43135852 +1	C -3.03457405 +1 -2.56970863 +1 3.39664933 +1
C -4.68753153 +1 -1.44333773 +1 2.04145283 +1	C -4.74312073 +1 -1.53367145 +1 2.03709746 +1
H -5.60195274 +1 -1.32060037 +1 5.31620906 +1	C -5.78597407 +1 -1.33172383 +1 5.66951086 +1
H -3.48029397 +1 -2.59259846 +1 5.54552827 +1	H -3.39850492 +1 -2.58632635 +1 5.51884809 +1
H -6.37361836 +1 -0.58437632 +1 3.07302218 +1	H -6.44025795 +1 -0.73852955 +1 3.09516568 +1
H -2.12554195 +1 -3.12597778 +1 3.53614261 +1	H -2.07430233 +1 -3.08276195 +1 3.48348320 +1
H -5.02979993 +1 -1.12017818 +1 1.05676759 +1	H -5.12389846 +1 -1.23577748 +1 1.05801326 +1
	H -6.78400686 +1 -0.92182520 +1 5.45852173 +1
	H -5.26679295 +1 -0.59327718 +1 6.30265574 +1
	H -5.93259814 +1 -2.22977605 +1 6.29048880 +1
**BAF/17g**	**PEA/17h**
C -0.00549430 +1 0.03886313 +1 0.08358552 +1	C -0.01538618 +1 0.01919212 +1 0.02657229 +1
C 1.41520864 +1 0.02801946 +1 -0.03101305 +1	C 1.40630407 +1 0.01026566 +1 -0.07530161 +1
C -0.66015249 +1 1.28368615 +1 0.24177356 +1	C -0.67527195 +1 1.26282847 +1 0.16351736 +1
C 2.13661415 +1 1.24820605 +1 0.07985327 +1	C 2.12346131 +1 1.23328007 +1 0.03206631 +1
C 0.03763328 +1 2.45946746 +1 0.32578317 +1	C 0.01877740 +1 2.44200117 +1 0.24073931 +1
C 1.45962433 +1 2.44258967 +1 0.25971834 +1	C 1.44087358 +1 2.42730577 +1 0.19132498 +1
C -0.69500047 +1 -1.22705947 +1 0.07645427 +1	C -0.70106296 +1 -1.25002549 +1 0.02862592 +1
C 0.03057004 +1 -2.36923600 +1 -0.40519651 +1	C 0.03026570 +1 -2.39078490 +1 -0.45195708 +1
N 2.12775162 +1 -1.11176338 +1 -0.23391340 +1	N 2.12349387 +1 -1.12900121 +1 -0.26353549 +1
C 1.45101799 +1 -2.26648807 +1 -0.46528999 +1	C 1.45061522 +1 -2.28601314 +1 -0.49898304 +1
C -0.57059118 +1 -3.56346480 +1 -0.87577290 +1	C -0.56546824 +1 -3.58459826 +1 -0.92848675 +1
C 0.18900356 +1 -4.64332157 +1 -1.24540636 +1	C 0.19865136 +1 -4.66515451 +1 -1.28827568 +1
C 2.22680396 +1 -3.42172572 +1 -0.80386758 +1	C 2.23062427 +1 -3.44064624 +1 -0.82761879 +1
C 1.60330606 +1 -4.58101991 +1 -1.17307302 +1	C 1.61151045 +1 -4.60154974 +1 -1.20082746 +1
N -1.94662546 +1 -1.26665000 +1 0.56489314 +1	N -1.94403024 +1 -1.29714497 +1 0.53284340 +1
C -2.68859232 +1 -2.48209550 +1 0.93819953 +1	C -2.68045476 +1 -2.52108931 +1 0.87741435 +1
N 2.23717209 +1 3.60910069 +1 0.39812478 +1	C -3.54831205 +1 -2.24590200 +1 2.12135223 +1
C 1.76199283 +1 4.91008803 +1 0.33814489 +1	N 2.21397627 +1 3.59766513 +1 0.32779019 +1
O 0.59689541 +1 5.17606921 +1 0.11435381 +1	C 1.74084028 +1 4.89567210 +1 0.21810103 +1
C 2.80332465 +1 5.97902894 +1 0.56456591 +1	O 0.58364785 +1 5.15409961 +1 -0.05168446 +1
C 2.11870569 +1 7.34217398 +1 0.63099493 +1	C 2.77441010 +1 5.96989338 +1 0.45488073 +1
N 3.10075938 +1 8.40281601 +1 1.05618708 +1	C 2.12179575 +1 7.34245584 +1 0.31054861 +1
C 3.46996385 +1 10.80193176 +1 1.06725654 +1	N 3.07390331 +1 8.42432339 +1 0.75037089 +1
C 2.40185148 +1 9.72999038 +1 1.30578824 +1	C 3.51063704 +1 10.80562808 +1 0.57302866 +1
C 4.56370020 +1 10.14741802 +1 0.20388039 +1	C 2.38920942 +1 9.78175693 +1 0.77645105 +1
C 4.14156203 +1 8.68989549 +1 -0.01339516 +1	C 4.68684905 +1 10.04710416 +1 -0.06843485 +1
H -1.75602099 +1 1.31745735 +1 0.28865837 +1	C 4.24841959 +1 8.58428992 +1 -0.20074574 +1
H 3.22413990 +1 1.21332808 +1 0.01650649 +1	H -1.77163367 +1 1.29363234 +1 0.19904122 +1
H -0.49407448 +1 3.40195725 +1 0.45420546 +1	H 3.21162207 +1 1.20104253 +1 -0.01864179 +1
H -1.65741296 +1 -3.61175154 +1 -0.98729848 +1	H -0.51557897 +1 3.38478323 +1 0.35189285 +1
H -0.28122520 +1 -5.55702629 +1 -1.60609523 +1	H -1.65096050 +1 -3.63290410 +1 -1.05222439 +1
H 3.31247561 +1 -3.33898767 +1 -0.77275722 +1	H -0.26771152 +1 -5.57964198 +1 -1.65231023 +1
H 2.18384231 +1 -5.46954802 +1 -1.43551010 +1	H 3.31566668 +1 -3.35703396 +1 -0.78609189 +1
H -2.42329200 +1 -0.39090817 +1 0.81173647 +1	H 2.19584152 +1 -5.48986512 +1 -1.45505316 +1
H -1.98965659 +1 -3.33330393 +1 1.13708904 +1	H -2.42608269 +1 -0.42415837 +1 0.77926690 +1
H -3.34835798 +1 -2.79781137 +1 0.08869356 +1	H -1.97372843 +1 -3.35789582 +1 1.09495004 +1
H 3.24939168 +1 3.46763410 +1 0.53974817 +1	H -3.32571232 +1 -2.83033716 +1 0.01852011 +1
H 3.56584386 +1 5.94608943 +1 -0.25096049 +1	H -4.30295752 +1 -1.45983799 +1 1.90355579 +1
H 3.37845670 +1 5.77194040 +1 1.50144912 +1	H -2.92748828 +1 -1.82444478 +1 2.94355250 +1
H 1.27406464 +1 7.32231208 +1 1.36844005 +1	H 3.22103337 +1 3.46019859 +1 0.50577562 +1
H 1.66198428 +1 7.61226716 +1 -0.35244201 +1	H 3.62742019 +1 5.84990735 +1 -0.25557177 +1
H 3.88871629 +1 11.17330280 +1 2.02219947 +1	H 3.22892204 +1 5.84497216 +1 1.47009452 +1
H 3.03726184 +1 11.69000300 +1 0.56899302 +1	H 1.19563778 +1 7.40587954 +1 0.93964656 +1

H 1.99219237 +1 9.74964312 +1 2.33917540 +1	H 1.79245477 +1 7.52403283 +1 -0.74156980 +1
H 1.54192021 +1 9.84862766 +1 0.61025456 +1	H 3.81353551 +1 11.26763771 +1 1.53215342 +1
H 5.55090495 +1 10.21200053 +1 0.69969973 +1	H 3.17242954 +1 11.64362509 +1 -0.06537247 +1
H 4.68477354 +1 10.67201588 +1 -0.76283995 +1	H 1.85334302 +1 9.91261721 +1 1.74165964 +1
H 3.70424282 +1 8.53994491 +1 -1.02549453 +1	H 1.62806732 +1 9.84425793 +1 -0.03237565 +1
H 4.98971060 +1 7.97949232 +1 0.08463097 +1	H 5.60103800 +1 10.14013937 +1 0.54819349 +1
H 3.58771283 +1 8.09908098 +1 1.94143284 +1	H 4.95254974 +1 10.47106230 +1 -1.05537601 +1
C -3.50095374 +1 -2.17235471 +1 2.16763283 +1	H 3.93556392 +1 8.34622883 +1 -1.24172464 +1
C -4.97258355 +1 -1.59159086 +1 4.41194317 +1	H 5.05415435 +1 7.86957989 +1 0.07038924 +1
C -3.70918664 +1 -2.15606742 +1 4.57648286 +1	H 3.43727082 +1 8.20381828 +1 1.71620934 +1
C -5.52647857 +1 -1.30793114 +1 3.16530948 +1	C -4.19756385 +1 -3.52252182 +1 2.55399000 +1
C -2.97220048 +1 -2.44511235 +1 3.43139203 +1	C -5.39633082 +1 -5.91028630 +1 3.36104643 +1
C -4.77052849 +1 -1.60629907 +1 2.03480102 +1	C -4.05659115 +1 -5.66624682 +1 3.65982432 +1
F -5.68415863 +1 -1.31131734 +1 5.49624830 +1	C -6.13527138 +1 -4.96100335 +1 2.65818834 +1
H -3.30277861 +1 -2.36561527 +1 5.56707088 +1	C -3.45732588 +1 -4.47562386 +1 3.25849725 +1
H -6.51857087 +1 -0.86502345 +1 3.06948451 +1	C -5.53804055 +1 -3.76890658 +1 2.25371747 +1
H -1.97562978 +1 -2.88627007 +1 3.52729041 +1	H -5.86488723 +1 -6.84221560 +1 3.67649737 +1
H -5.17641935 +1 -1.39426189 +1 1.04146824 +1	H -3.47669568 +1 -6.40893788 +1 4.20890221 +1
	H -7.18312954 +1 -5.14958718 +1 2.42316735 +1
	H -2.40942404 +1 -4.28554778 +1 3.49427049 +1
	H -6.11906894 +1 -3.02972098 +1 1.70222338 +1
**PPA/17i**	**PBA/17j**
C -0.03585966 +1 0.03006460 +1 0.04576802 +1	C 0.02404846 +1 -0.02037471 +1 -0.04474013 +1
C 1.38874652 +1 0.00699696 +1 0.01543578 +1	C 1.44161603 +1 -0.01439721 +1 0.09755011 +1
C -0.69064075 +1 1.28077349 +1 0.12851332 +1	C -0.64741892 +1 1.21817766 +1 -0.17464716 +1
C 2.11107846 +1 1.22526032 +1 0.13983453 +1	C 2.14733912 +1 1.21948682 +1 0.04374850 +1
C 0.00976134 +1 2.45538084 +1 0.22026510 +1	C 0.03470062 +1 2.40615323 +1 -0.21131747 +1
C 1.43207084 +1 2.42758695 +1 0.24258935 +1	C 1.45480769 +1 2.40547412 +1 -0.12229457 +1
C -0.73326695 +1 -1.23363636 +1 0.03387521 +1	C -0.65080496 +1 -1.29663634 +1 -0.07798367 +1
C 0.00998764 +1 -2.38657996 +1 -0.39850044 +1	C 0.08647459 +1 -2.44099490 +1 0.38696087 +1
N 2.10352645 +1 -1.14150878 +1 -0.12126547 +1	N 2.16802728 +1 -1.14981459 +1 0.27260052 +1
C 1.43196054 +1 -2.29501179 +1 -0.37670553 +1	C 1.50491292 +1 -2.32093416 +1 0.45973270 +1
C -0.57309645 +1 -3.58027415 +1 -0.88938120 +1	C -0.49852941 +1 -3.65727719 +1 0.81709165 +1
C 0.19749534 +1 -4.67144792 +1 -1.20109860 +1	C 0.27438346 +1 -4.73944652 +1 1.15358630 +1
C 2.21585473 +1 -3.46028139 +1 -0.65409949 +1	C 2.29553373 +1 -3.47517865 +1 0.76362843 +1
C 1.60474577 +1 -4.61951870 +1 -1.04558766 +1	C 1.68712968 +1 -4.65565837 +1 1.08982790 +1
N -1.99563877 +1 -1.26310718 +1 0.48517895 +1	N -1.88555199 +1 -1.34001739 +1 -0.59943456 +1
C -2.76979747 +1 -2.47346717 +1 0.79761960 +1	C -2.64349232 +1 -2.55741292 +1 -0.92399656 +1
C -3.69245609 +1 -2.17586494 +1 1.99173501 +1	C -3.61869862 +1 -2.24804166 +1 -2.07227689 +1
C -4.48791699 +1 -3.43824910 +1 2.34941111 +1	C -4.39332810 +1 -3.51716564 +1 -2.43622864 +1
N 2.20653209 +1 3.59504324 +1 0.39771555 +1	C -5.37819489 +1 -3.23250263 +1 -3.58071791 +1
C 1.75352445 +1 4.89119816 +1 0.21164601 +1	C -6.11935911 +1 -4.48498424 +1 -3.92903384 +1
O 0.61996395 +1 5.14653349 +1 -0.14743742 +1	C -7.48365880 +1 -6.84000980 +1 -4.55452191 +1
C 2.77556894 +1 5.96800471 +1 0.48337978 +1	C -8.01593828 +1 -5.95135661 +1 -3.62225126 +1
C 2.14533942 +1 7.33669547 +1 0.23815248 +1	C -6.26942061 +1 -6.55078298 +1 -5.17462677 +1
N 3.07475861 +1 8.43180848 +1 0.69344492 +1	C -7.33668458 +1 -4.77650138 +1 -3.30937496 +1
C 3.53645995 +1 10.80214388 +1 0.44077999 +1	C -5.58803475 +1 -5.37688272 +1 -4.86383244 +1
C 2.39863587 +1 9.79173507 +1 0.61838575 +1	N 2.20348206 +1 3.60072856 +1 -0.14970557 +1
C 4.74671191 +1 10.01229103 +1 -0.08984174 +1	C 1.76537292 +1 4.79547621 +1 -0.69721289 +1
C 4.30905251 +1 8.54646276 +1 -0.18580335 +1	O 0.67745581 +1 4.91017688 +1 -1.22903962 +1
H -1.78703923 +1 1.32109917 +1 0.10898178 +1	C 2.73978137 +1 5.94280228 +1 -0.59262867 +1
H 3.19991783 +1 1.18345336 +1 0.14579545 +1	C 2.12412072 +1 7.18637171 +1 -1.22921298 +1
H -0.52036969 +1 3.40453877 +1 0.28871550 +1	N 3.01378483 +1 8.38285937 +1 -1.01265583 +1
H -1.65210376 +1 -3.62138521 +1 -1.06251528 +1	C 3.47765022 +1 10.62101019 +1 -1.83138616 +1
H -0.25943388 +1 -5.58535620 +1 -1.57760782 +1	C 2.33614485 +1 9.66231222 +1 -1.47780743 +1
H 3.29836727 +1 -3.38634824 +1 -0.55978394 +1	C 4.72661852 +1 9.75069050 +1 -2.06130174 +1
H 2.19223021 +1 -5.51579904 +1 -1.26193575 +1	C 4.30399064 +1 8.29858122 +1 -1.81153895 +1
H -2.47731836 +1 -0.38130922 +1 0.70238151 +1	H -1.74323901 +1 1.23828978 +1 -0.22965619 +1
H -2.08883094 +1 -3.32062800 +1 1.05419067 +1	H 3.23273023 +1 1.20099009 +1 0.13805551 +1
H -3.37184746 +1 -2.77637653 +1 -0.09392764 +1	H -0.50513106 +1 3.34887737 +1 -0.29391516 +1
H -4.38491110 +1 -1.34245504 +1 1.75850868 +1	H -1.58420864 +1 -3.72571552 +1 0.92483033 +1
H -3.09831395 +1 -1.83769901 +1 2.86538972 +1	H -0.18588977 +1 -5.67103066 +1 1.47984559 +1
H -3.79916376 +1 -4.29748134 +1 2.50201780 +1	H 3.37992326 +1 -3.37519456 +1 0.74274015 +1
H -5.13553906 +1 -3.73666305 +1 1.49801947 +1	H 2.27941442 +1 -5.54425898 +1 1.32391525 +1
H 3.19853023 +1 3.45548322 +1 0.64583826 +1	H -2.36029190 +1 -0.46219939 +1 -0.85049283 +1
H 3.67820773 +1 5.81653009 +1 -0.15626710 +1	H -3.20760842 +1 -2.90286826 +1 -0.02291206 +1
H 3.15281700 +1 5.88021699 +1 1.53350707 +1	H -1.94945710 +1 -3.37794524 +1 -1.22834790 +1
H 1.18001028 +1 7.43162023 +1 0.80085977 +1	H -3.06924781 +1 -1.86556768 +1 -2.95627552 +1
H 1.88758881 +1 7.47469945 +1 -0.84031154 +1	H -4.31990884 +1 -1.43961622 +1 -1.78259869 +1
H 3.78054826 +1 11.30633018 +1 1.39542037 +1	H -4.94226797 +1 -3.90223500 +1 -1.55424861 +1
H 3.24407234 +1 11.61126158 +1 -0.25501922 +1	H -3.69487314 +1 -4.32495808 +1 -2.73294494 +1
H 1.80511168 +1 9.96628320 +1 1.54206513 +1	H -4.83269393 +1 -2.83454139 +1 -4.46200991 +1
H 1.68925423 +1 9.82192417 +1 -0.23801317 +1	H -6.07757707 +1 -2.42236024 +1 -3.28715665 +1
H 5.62009055 +1 10.12957909 +1 0.57954230 +1	H -8.01597486 +1 -7.75878980 +1 -4.79847995 +1
H 5.07754949 +1 10.39145597 +1 -1.07535479 +1	H -8.96598840 +1 -6.17480879 +1 -3.13585190 +1
H 4.06264020 +1 8.26371583 +1 -1.23349249 +1	H -5.85110654 +1 -7.24439501 +1 -5.90474786 +1
H 5.09143278 +1 7.84212109 +1 0.16820753 +1	H -7.75252162 +1 -4.08429445 +1 -2.57793248 +1
H 3.37377767 +1 8.25040263 +1 1.68909564 +1	H -4.63701197 +1 -5.15295729 +1 -5.34668965 +1
C -5.29953299 +1 -3.18594754 +1 3.58201165 +1	H 3.16490800 +1 3.54944011 +1 0.21726715 +1
C -6.79672305 +1 -2.69067645 +1 5.88638659 +1	H 3.70814170 +1 5.67396839 +1 -1.07918481 +1
C -7.36582075 +1 -2.49830246 +1 4.62906168 +1	H 3.00254930 +1 6.12574070 +1 0.47986555 +1
C -5.47834266 +1 -3.13086514 +1 5.99170024 +1	H 1.12029288 +1 7.40102609 +1 -0.77855002 +1
C -6.62031159 +1 -2.74508712 +1 3.47861757 +1	H 1.94442095 +1 7.03441167 +1 -2.32156879 +1
C -4.73077293 +1 -3.37839419 +1 4.84356700 +1	H 3.65130666 +1 11.35673229 +1 -1.02299724 +1
H -7.38104637 +1 -2.49691310 +1 6.78543913 +1	H 3.23019563 +1 11.21966523 +1 -2.72849210 +1
H -8.39661692 +1 -2.15297764 +1 4.54394625 +1	H 1.67732693 +1 10.05130762 +1 -0.67151751 +1

H -5.03055073 +1 -3.28085582 +1 6.97468110 +1<br>H -7.06645610 +1 -2.59007568 +1 2.49663310 +1<br>H -3.69827996 +1 -3.71868039 +1 4.92697452 +1	H 1.68923361 +1 9.45672280 +1 -2.35917519 +1<br>H 5.54941892 +1 10.05302081 +1 -1.38585169 +1<br>H 5.12480785 +1 9.87630429 +1 -3.08602850 +1<br>H 4.12978710 +1 7.75605681 +1 -2.76728895 +1<br>H 5.06420467 +1 7.72340467 +1 -1.24163396 +1<br>H 3.24629004 +1 8.46852910 +1 0.01289571 +1

Table S5. Top five most populated cluster with the lowest binding energy (kcal/mol) for the derivatives **17a**–**17j** in the cleavage complex with *h*Topo I (pdb id: 1T8I) proposed by docking simulations.

Ligand	cluster _01	energy _01	cluster _02	energy _02	cluster _03	energy _03	cluster _04	energy _04	cluster _05	energy _05
**17a**	16	-10.95	30	-10.74	33	-10.66	24	-10.12	15	-9.62
**17b**	8	-11.81	59	-11.13	18	-11.06	19	-10.69	12	-10.68
**17c**	33	-11.09	10	-10.5	19	-10.2	23	-10.16	10	-10.04
**17d**	37	-10.93	16	-10.66	28	-10.05	16	-9.78	17	-9.64
**17e**	26	-11.16	17	-11.14	22	-10.95	9	-10.86	-	-
**17f**	11	-11.37	17	-11.22	17	-11.03	10	-10.88	10	-10.41
**17g**	30	-10.79	13	-10.44	16	-10.24	14	-9.74	-	-
**17h**	36	-11.42	8	-10.58	12	10.52	10	9.93	9	9.38
**17i**	10	-12.44	33	-12.28	7	-11.95	11	-11.33	12	-10.56
**17j**	3	-11.98	21	-11.66	15	-11.04	15	-10.68	-	-

Table S6. Top five most populated cluster with the lowest binding energy (kcal/mol) for the derivatives **17a**–**17j** in the cleavage complex with *h*Topo IIα (pdb id: 5GWK) proposed by docking simulations.

Ligand	cluster _01	energy _01	cluster _02	energy _02	cluster _03	energy _03	cluster _04	energy _04	cluster _05	energy _05
**17a**	42	-11.35	45	-11.32	20	-11.29	14	-11.04	15	-10.79
**17b**	33	-12.35	37	-11.73	24	-11.6	25	-11.18	15	-11.07
**17c**	39	-12.12	42	-11.98	14	-11.77	21	-11.5	12	-11.28
**17d**	56	-11.42	10	-11.02	15	-10.83	17	-10.8	15	-10.59
**17e**	40	-11.6	17	-11.32	14	-11.03	21	11.01	18	-10.93
**17f**	12	-11.8	30	-11.78	23	-11.41	42	-11.19	15	-11.07
**17g**	35	-11.51	12	-11.13	29	-10.83	31	-10.08	12	-10.5
**17h**	38	-11.81	18	-11.76	28	-11.64	15	-11.17	16	-11.06
**17i**	14	-12.56	19	-12.23	19	-12.17	16	-12.06	22	-11.9
**17j**	24	-12.55	17	-12.48	12	-12.38	15	-12.3	17	-12.13

Table S7. Top five most populated cluster with the lowest binding energy (kcal/mol) for the derivatives **17a**–**17j** in the cleavage complex with *h*Topo IIβ (pdb id: 4G0U) proposed by docking simulations.

Ligand	cluster _01	energy _01	cluster _02	energy _02	cluster _03	energy _03	cluster _04	energy _04	cluster _05	energy _05
**17a**	90	-11.77	61	-11.6	11	-10.78	7	-9.73	9	-9.17
**17b**	30	-12.08	24	-11.98	32	-11.72	14	-10.79	17	-10.34
**17c**	74	-12.44	36	-11.85	12	-10.26	12	-9.89	13	-9.73
**17d**	73	-11.5	59	-11.06	10	-10.08	6	9.86	14	-9.47
**17e**	74	-12.07	53	-11.96	10	-10.28	8	-10.24	10	-9.35
**17f**	63	-12.25	19	-11.5	12	-10.52	18	-10.06	16	-9.09
**17g**	66	-11.32	23	-10.73	13	-10.29	12	-9.89	12	-9.36
**17h**	72	-12.61	51	-12.33	16	-10.13	9	-9.75	9	-9.07
**17i**	59	-13.04	12	-12.52	10	-11.17	20	-10.75	9	-10.22
**17j**	40	-12.59	23	-12.37	9	-11.26	16	-10.71	9	-10.46

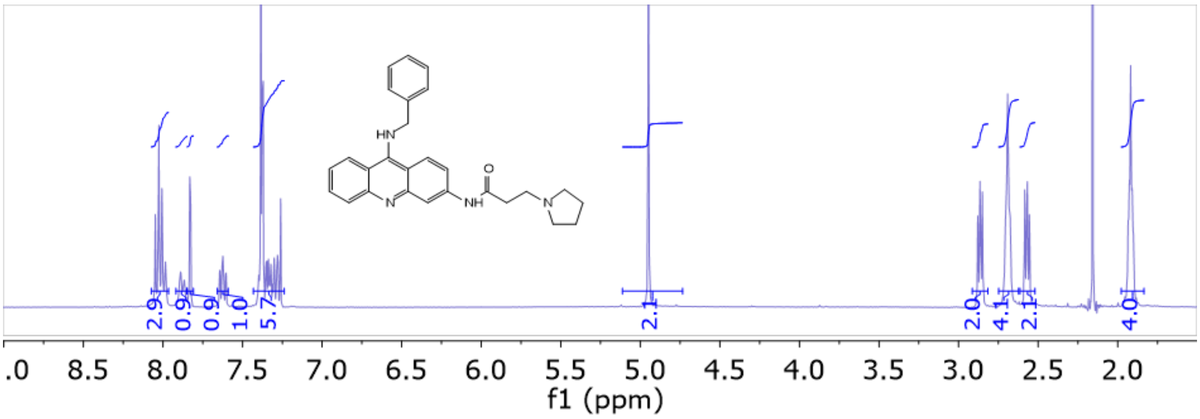

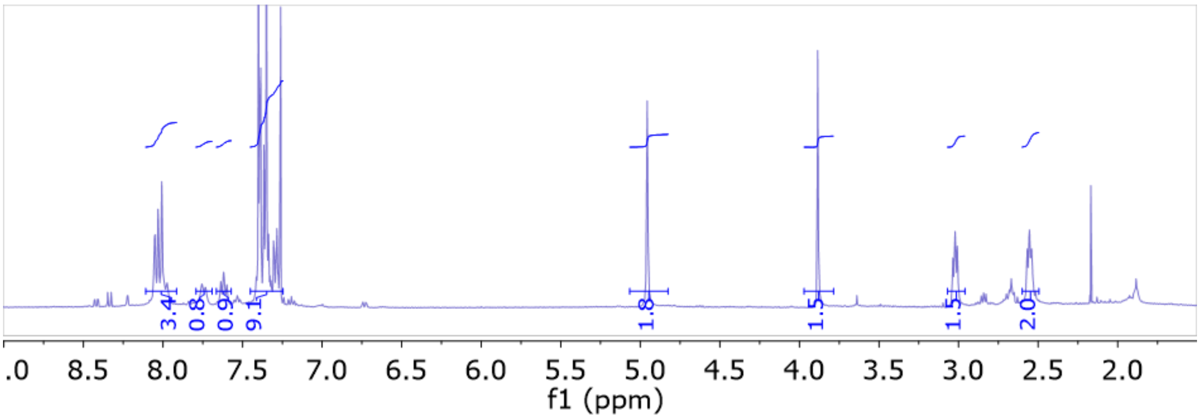

Figure S1. ^{1}H NMR spectra of **17e** (upper) and of **17k** (lower) produced through the reaction of chloroacridine **16** and benzylamine.

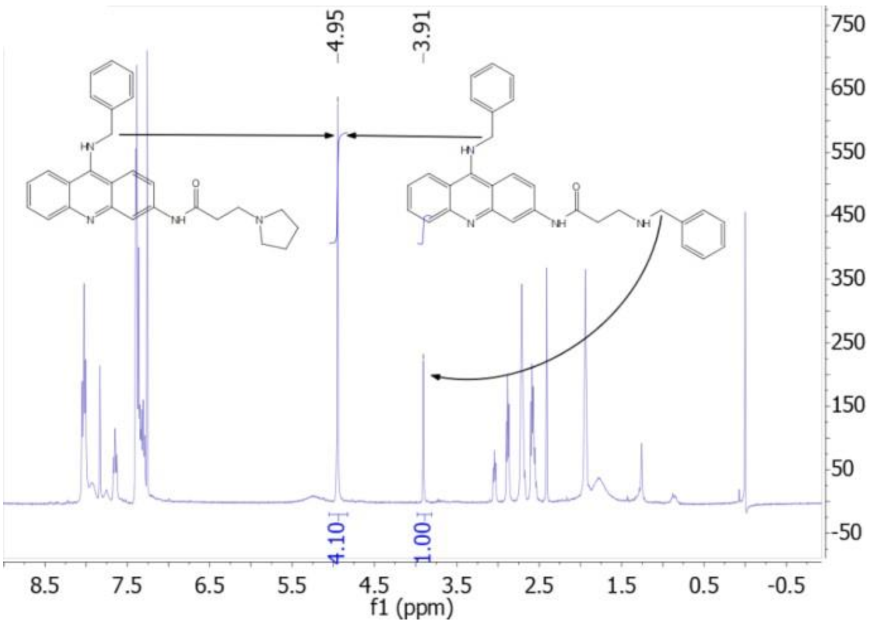

Figure S2. ^{1}H NMR spectra (400 MHz, $CDCl_3$) of the crude reaction mixture of the reaction of choroacridine **16** and benzylamine in DMF at 100 °C after 2 h.

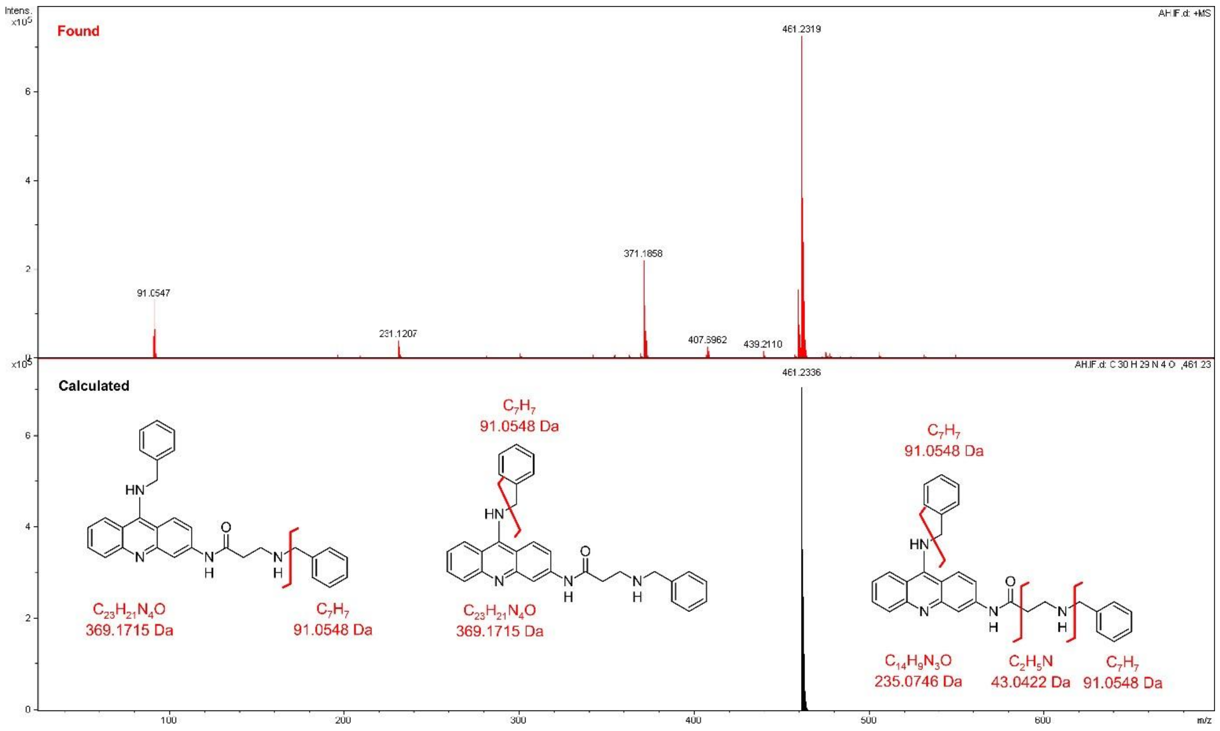

Figure S3. HRMS (ESI+) spectrum of side product **17k** with its hypothesised fragmentation.

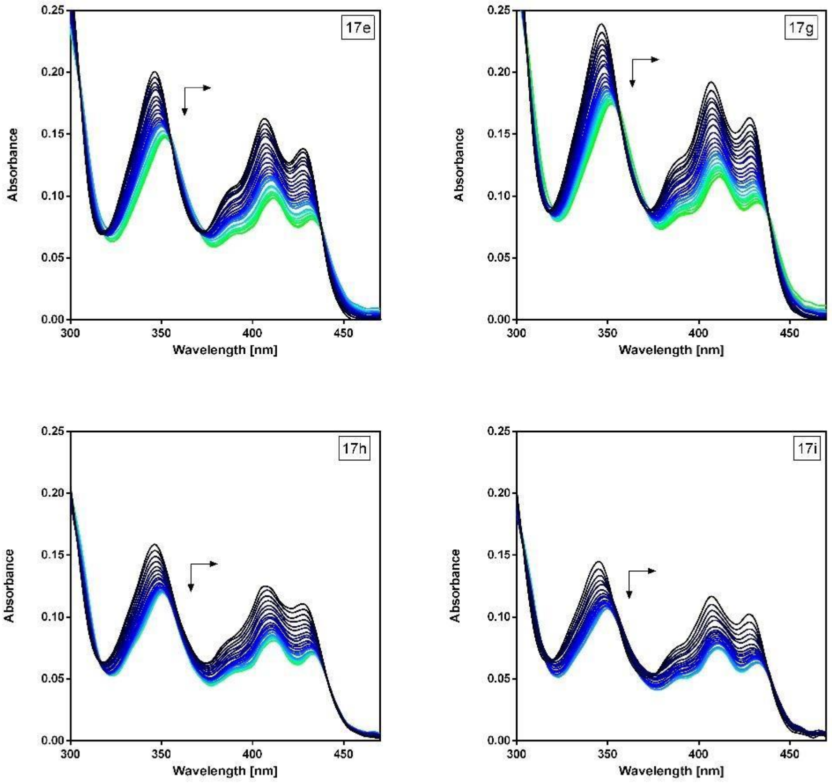

Absorption titration spectra of compounds **17e**, **17g**–**17i** (16 µM) in the absence and presence of *ct*DNA (**17e**: 0–31.4 µM; **17g**: 0–32.8 µM; **17h**: 0–27.4 µM; **17i**: 0–24.6 µM).

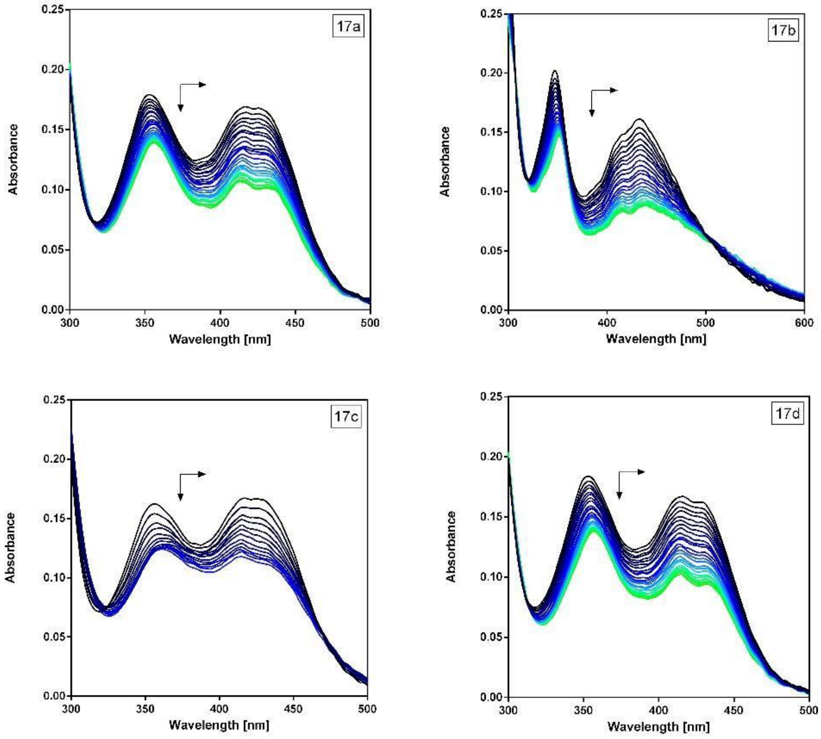

Absorbtion titration spectra of compounds **17a**–**17d** (16 µM) in the absence and presence of *ct*DNA (**17a**: 0–33 µM; **17d**: 0–33 µM; **17c**: 0–17.3 µM; **17b**: 0–31.5 µM).

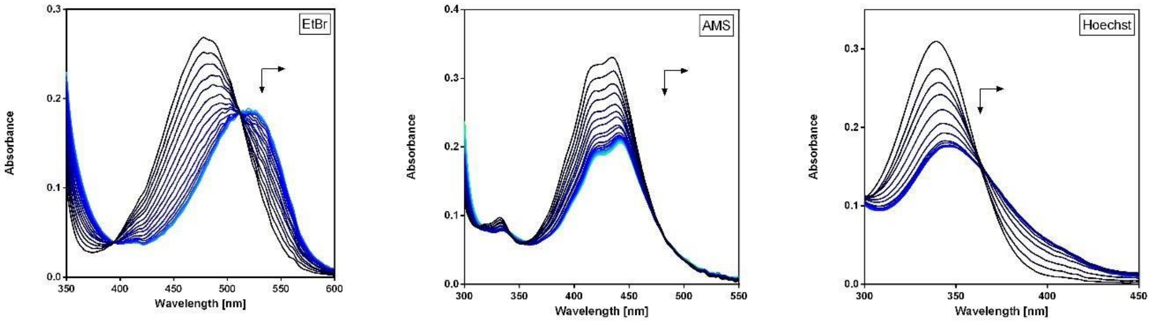

Absorbtion titration spectra of compounds **EtBr** (47 µM), **AMS** (32 µM) and **Hoechst** (6.4 µM) the absence and presence of *ct*DNA (**EtBr**: 0–133.4 µM; **AMS**: 0–217.7 µM; **Hoechst**: 0–8.6 µM).

Figure S4. Absorbtion titration spectra of compounds **17a**–**17i**, **EtBr**, **AMS** and **Hoechst** in Tris-HCl buffer (pH 7.4) in the absence and presence of *ct*DNA. Arrows indicate change in absorption spectra upon increasing *ct*DNA concentration

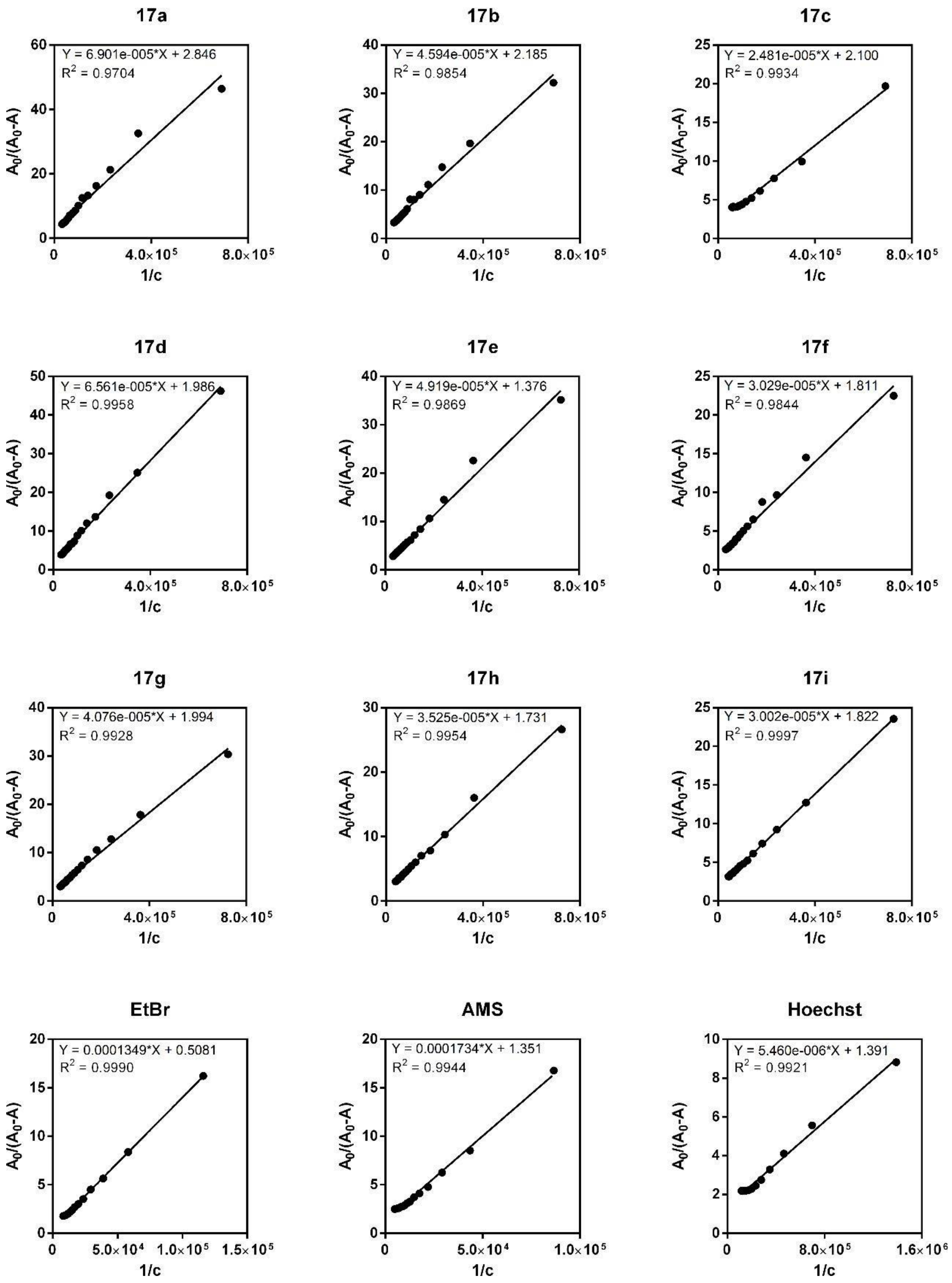

Figure S5. Selected Benesi-Hildebrand plots for studied ligands **17a**–**17i** and reference molecules **EtBr**, **AMS** and **Hoechst**.

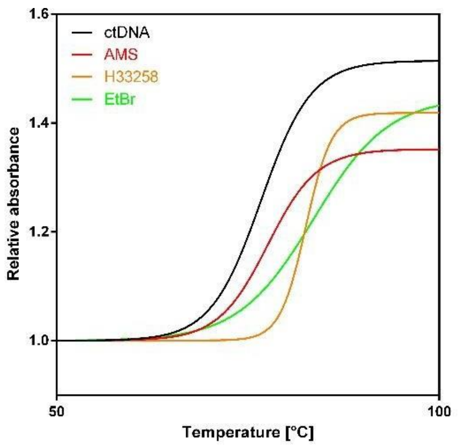

Figure S6. Absorption thermal denaturation profiles of *ct*DNA (145 µM) in absence (black line) and presence of reference molecules (20 µM **EtBr**, **AMS** and 1.3 µM **Hoechst**, respectively) in BPES buffer (pH 7.1) measured at 260 nm.

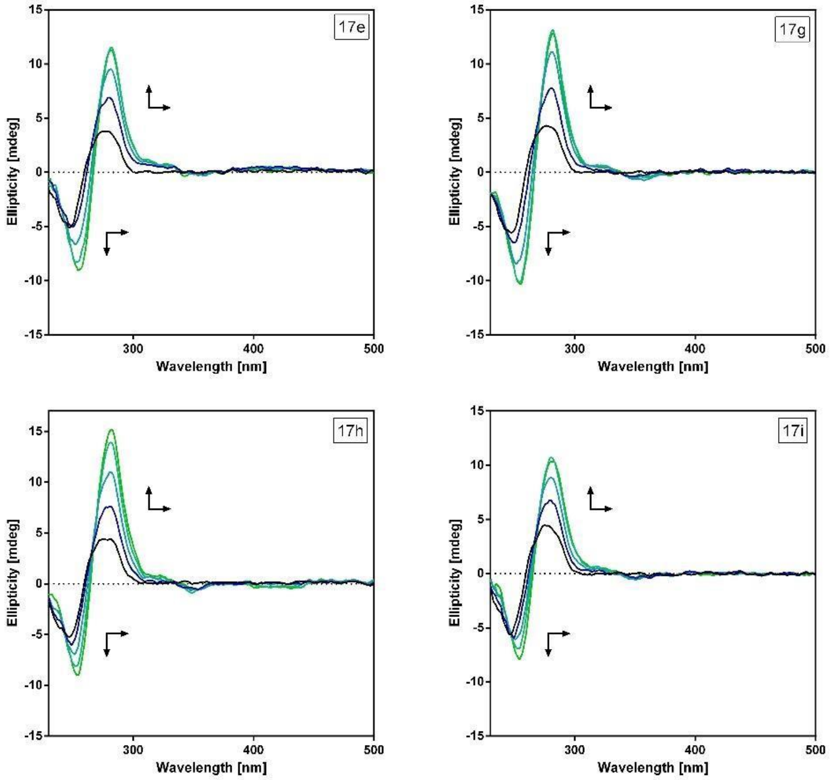

Circular dichroism spectra of *ct*DNA (28 µM) in absence or presence of the studied compounds **17e**, **17g**–**17i** (0–16 µM).

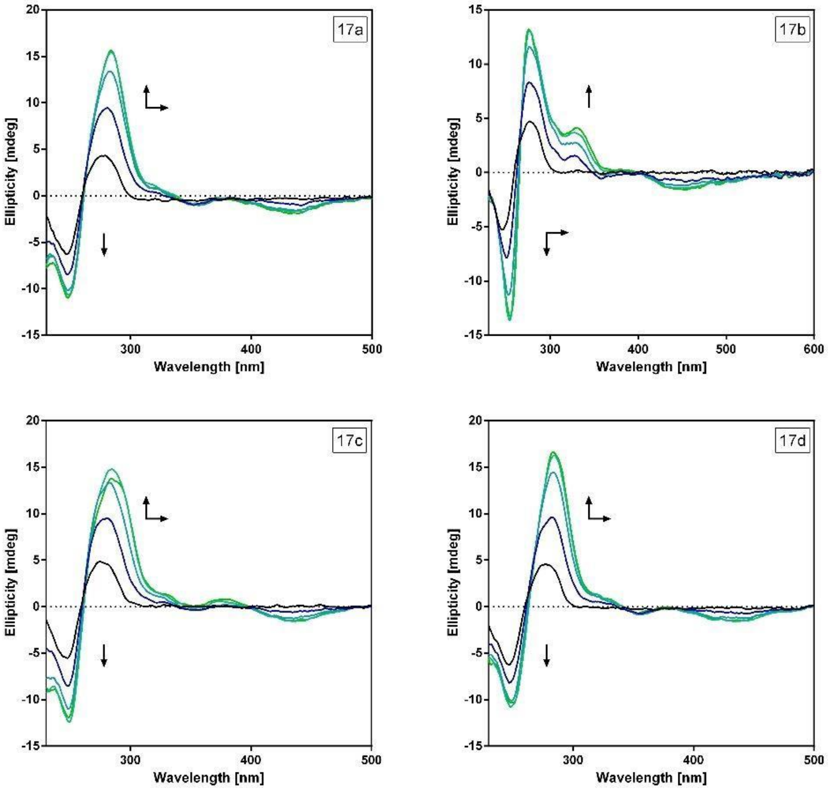

Circular dichroism spectra of *ct*DNA (29 µM) in absence or presence of the studied compounds **17a**–**17d** (0–16 µM).

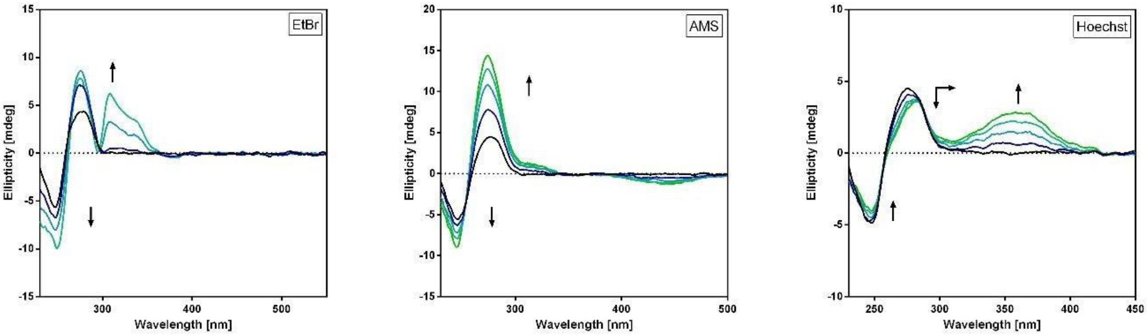

Circular dichroism spectra of ctDNA (29 µM) in absence or presence of the compounds **EtBr** (0–12 µM), **AMS** (0–16 µM) and **Hoechst** (0–1.3 µM).

Figure S7. Circular dichroism spectra of ctDNA in Tris-HCl buffer (pH 7.4) in absence or presence of the studied compounds **17a**–**17i**, **EtBr**, **AMS** and **Hoechst**. Arrows indicate the changes in circular dichroism spectra.

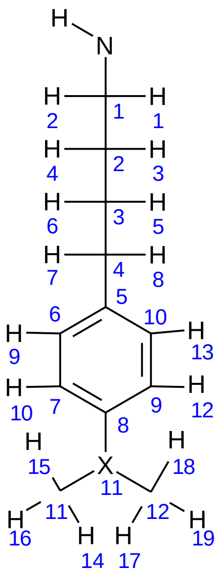

Figure S8. Numbering of the atoms within a substituent located at position 9 of the acridine core for derivatives **17a**–**17j**.

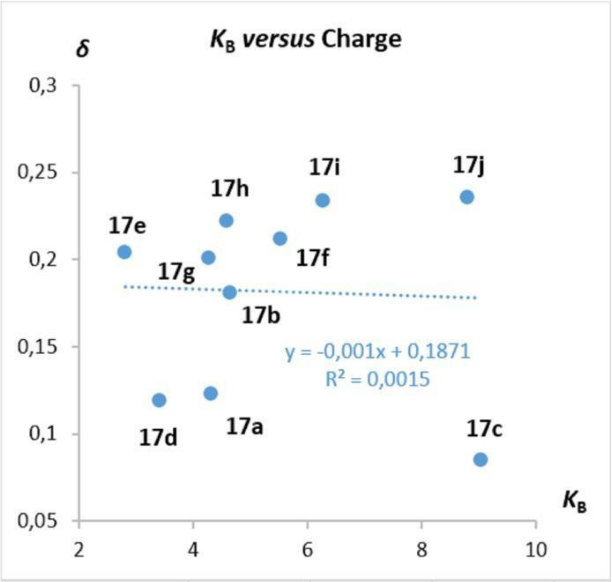

Figure S9. Relationship of binding constant K_B and the sum [δ] of partial charges of atoms within a substituent in position 9 of the acridine scaffold for acridine derivatives **17a**–**17j** obtained using the PM7 method. The binding constants $K_B \times 10^4$ of the *ct*DNA – acridines **17a**–**17j** intercalation complexes are given as a molar concentration.

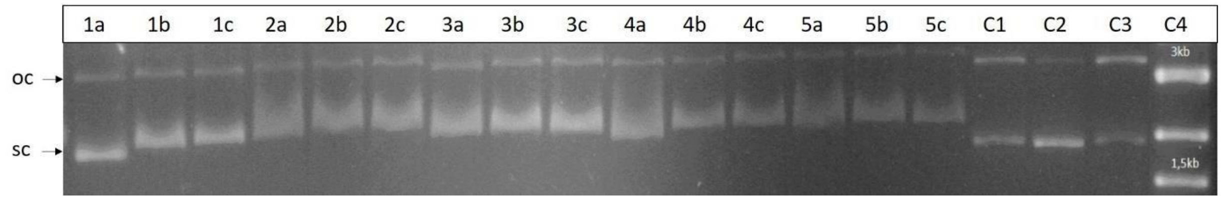

Sample order: 1 – **17i**, 2 – **17h**, 3 – **17e**, 4 – **17f**, 5 – **17g**, C1 – incubated pUC19, C2 – non-incubated pUC19, C3 – pUC19 + DMSO; C4 – 1kb ladder.

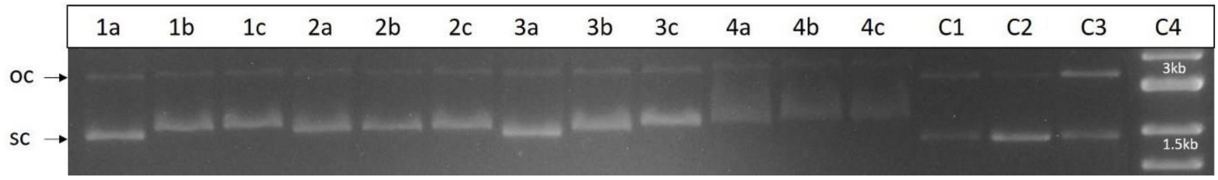

Sample order: 1 – **17a**, 2 – **17d**, 3 – **17c**, 4 – **17b**, C1 – incubated pUC19, C2 – non-incubated pUC19, C3 – pUC19 + DMSO; C4 – 1kb ladder.

Figure S10. Electrophoretic record of nuclease activity of studied compounds **17a**–**17i** on pUC19 plasmid DNA. Concentrations: a – 10 µM, b – 50 µM, c – 100 µM. Abbreviations: oc – open circular form, sc – supercoiled form.

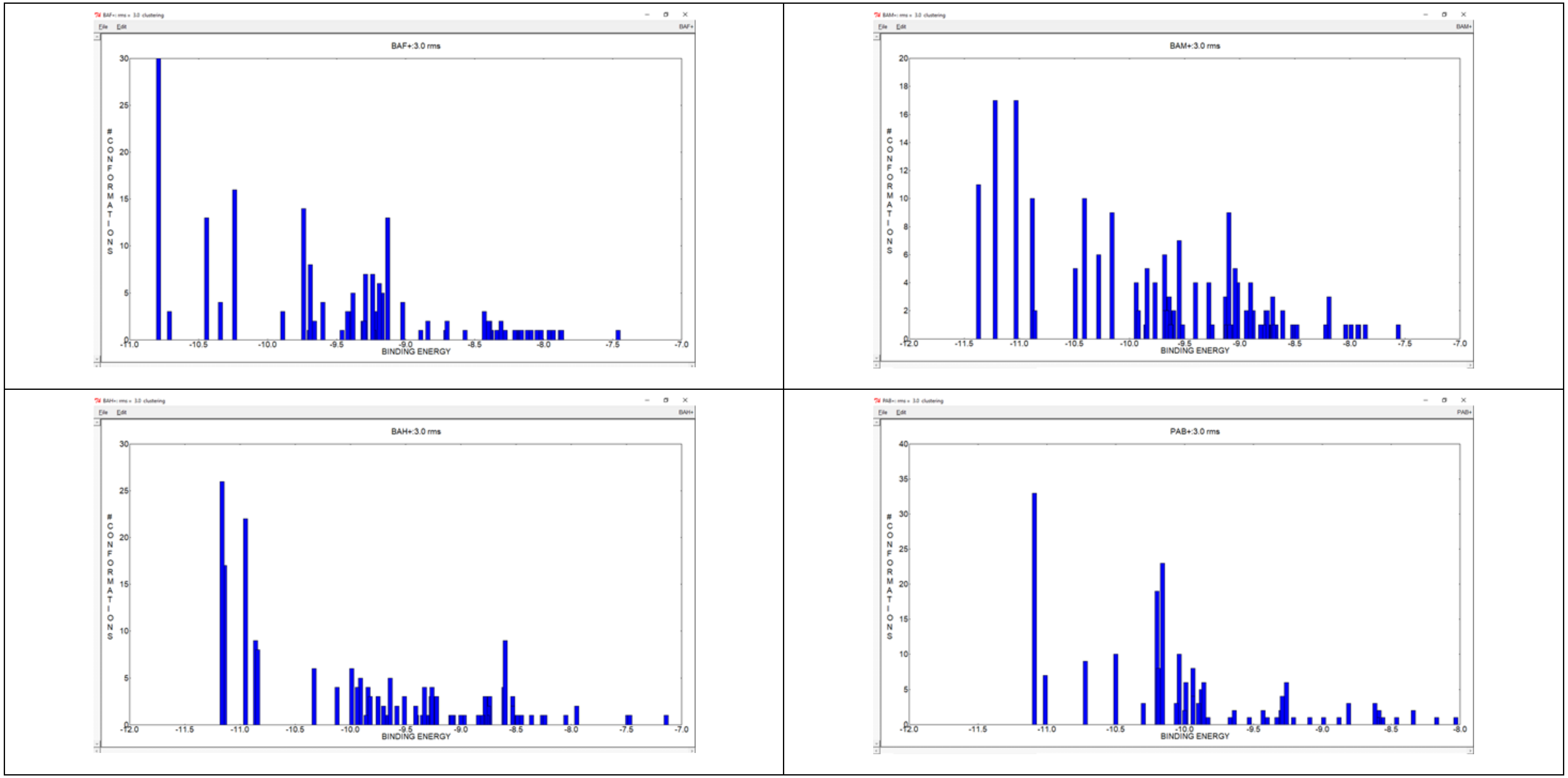

Figure S11. Clustering histogram of the molecular docking simulations for the derivatives **17a**–**17j** in the cleavage complex with *h*Topo I (pdb id: 1T8I).

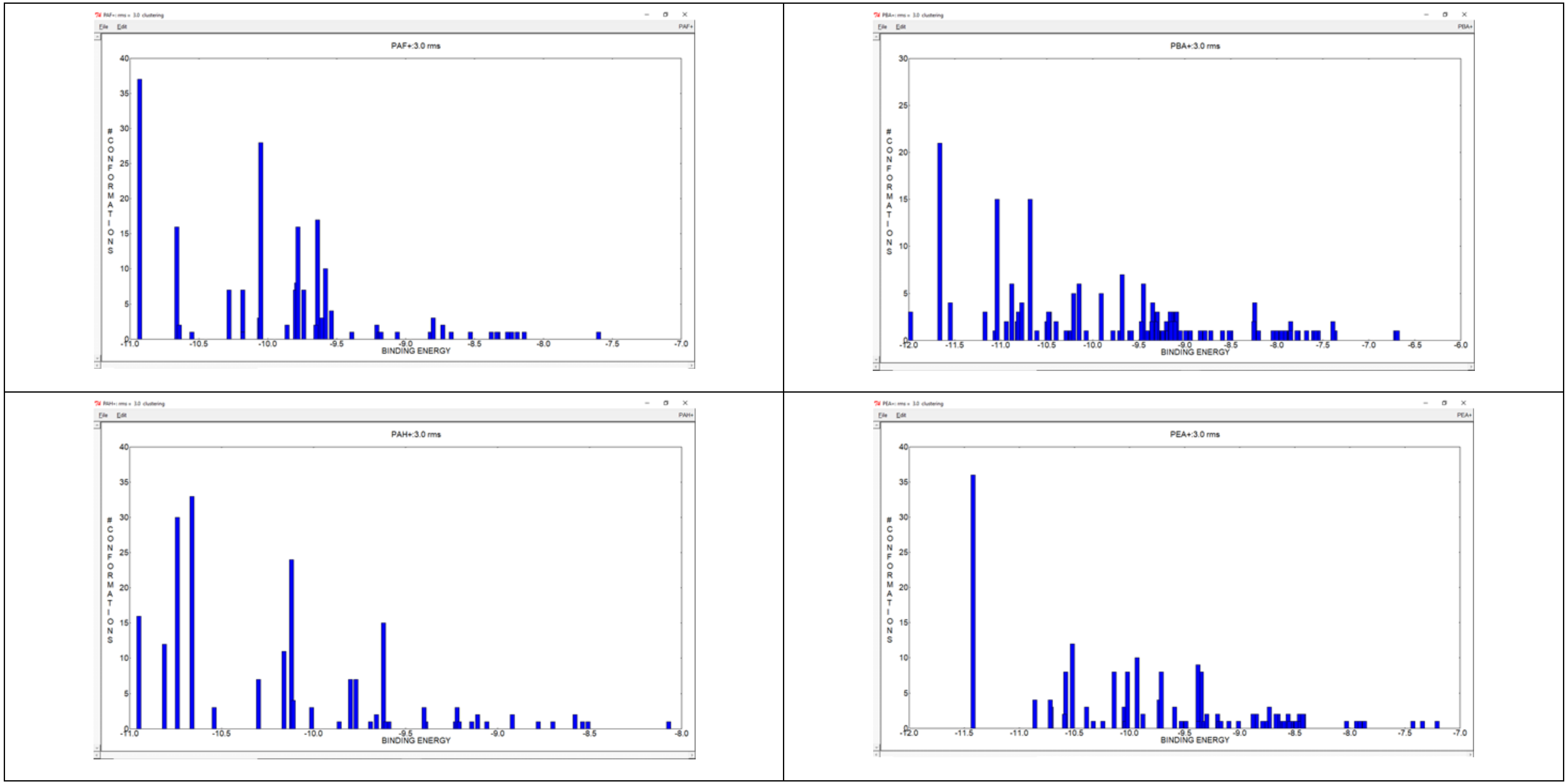

Figure S12. Clustering histogram of the molecular docking simulations for the derivatives **17a**–**17j** in the cleavage complex with *h*Topo I (pdb id: 1T8I).

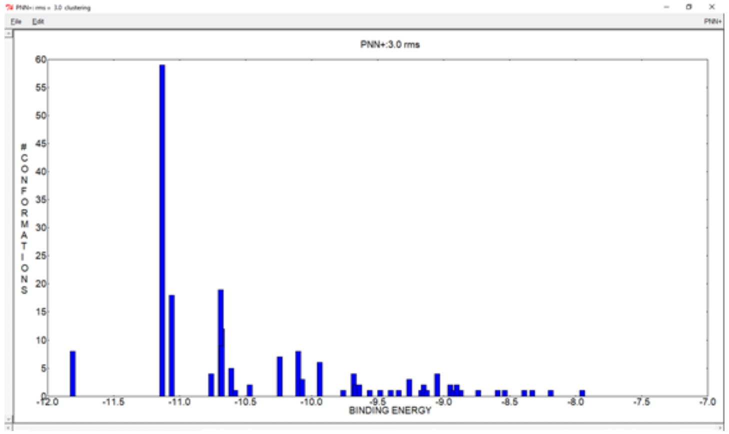

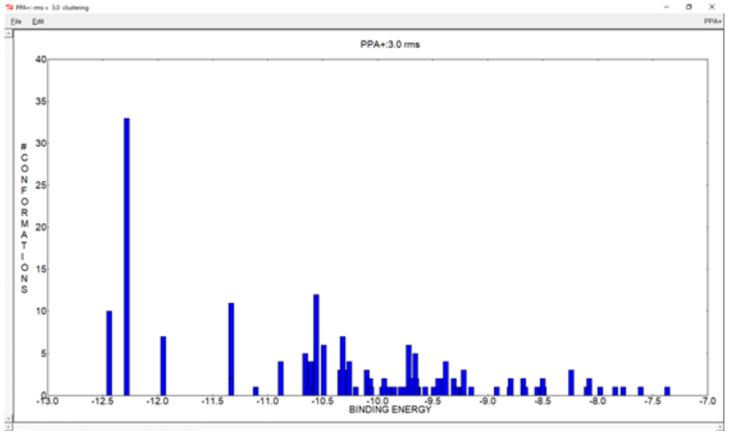

Figure S13. Clustering histogram of the molecular docking simulations for the derivatives **17a**–**17j** in the cleavage complex with *h*Topo I (pdb id: 1T8I).

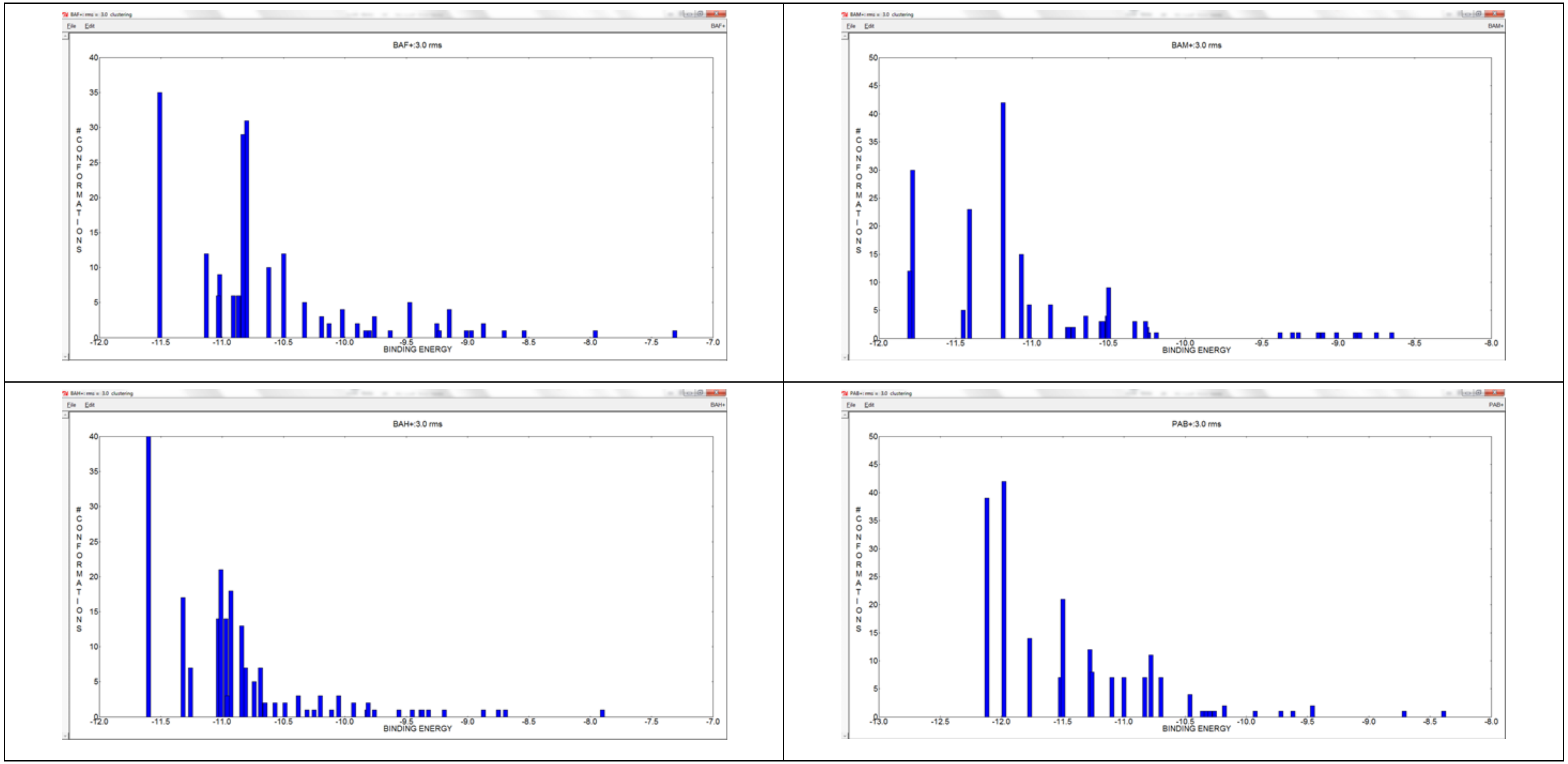

Figure S14. Clustering histogram of the molecular docking simulations for the derivatives **17a–17j** in the cleavage complex with *h*Topo IIα (pdb id: 5GWK).

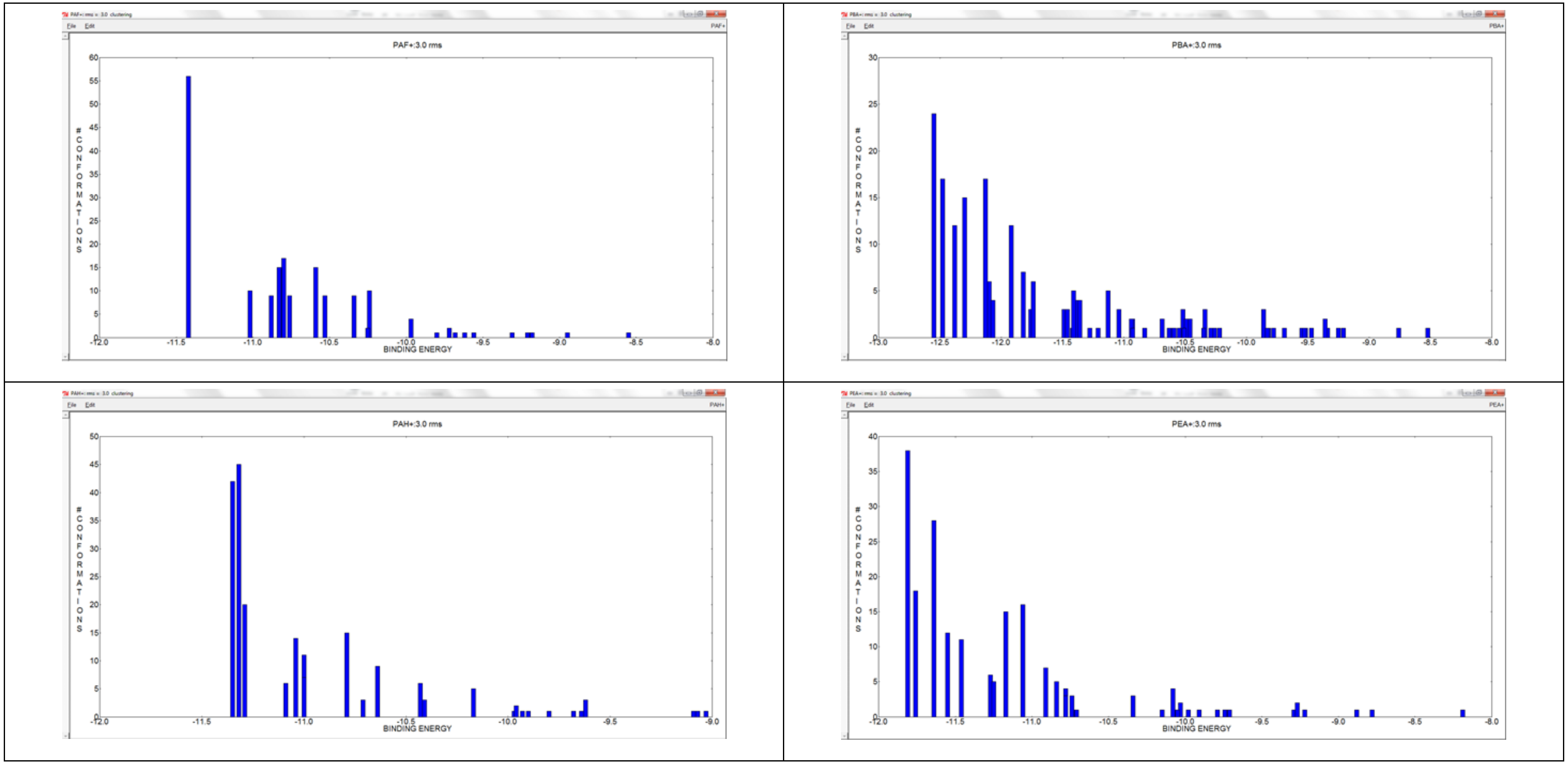

Figure S15. Clustering histogram of the molecular docking simulations for the derivatives **17a**–**17j** in the cleavage complex with *h*Topo IIα (pdb id: 5GWK).

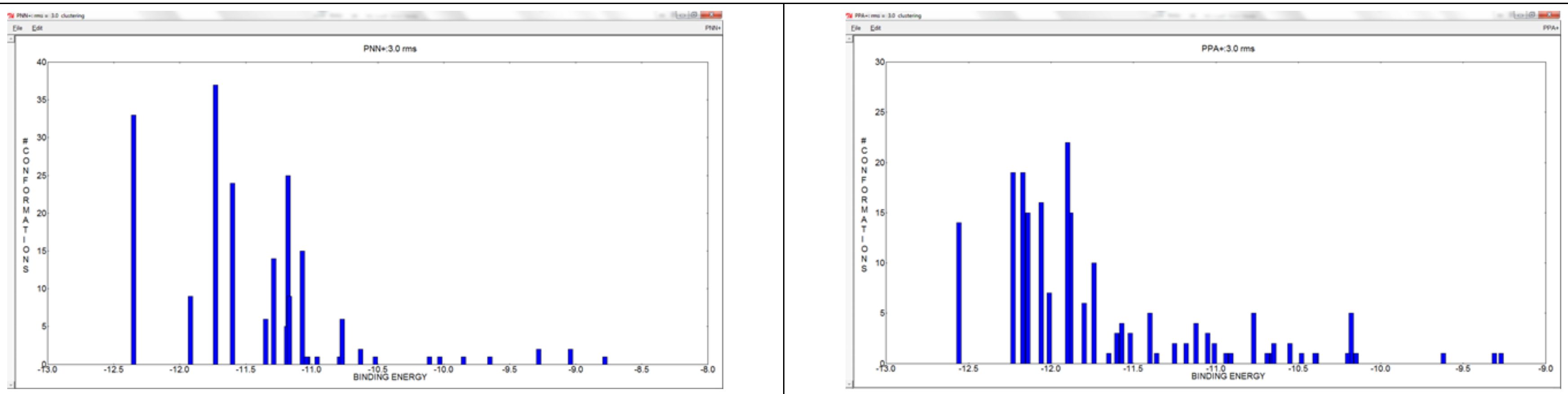

Figure S16. Clustering histogram of the molecular docking simulations for the derivatives **17a**–**17j** in the cleavage complex with *h*Topo IIα (pdb id: 5GWK).

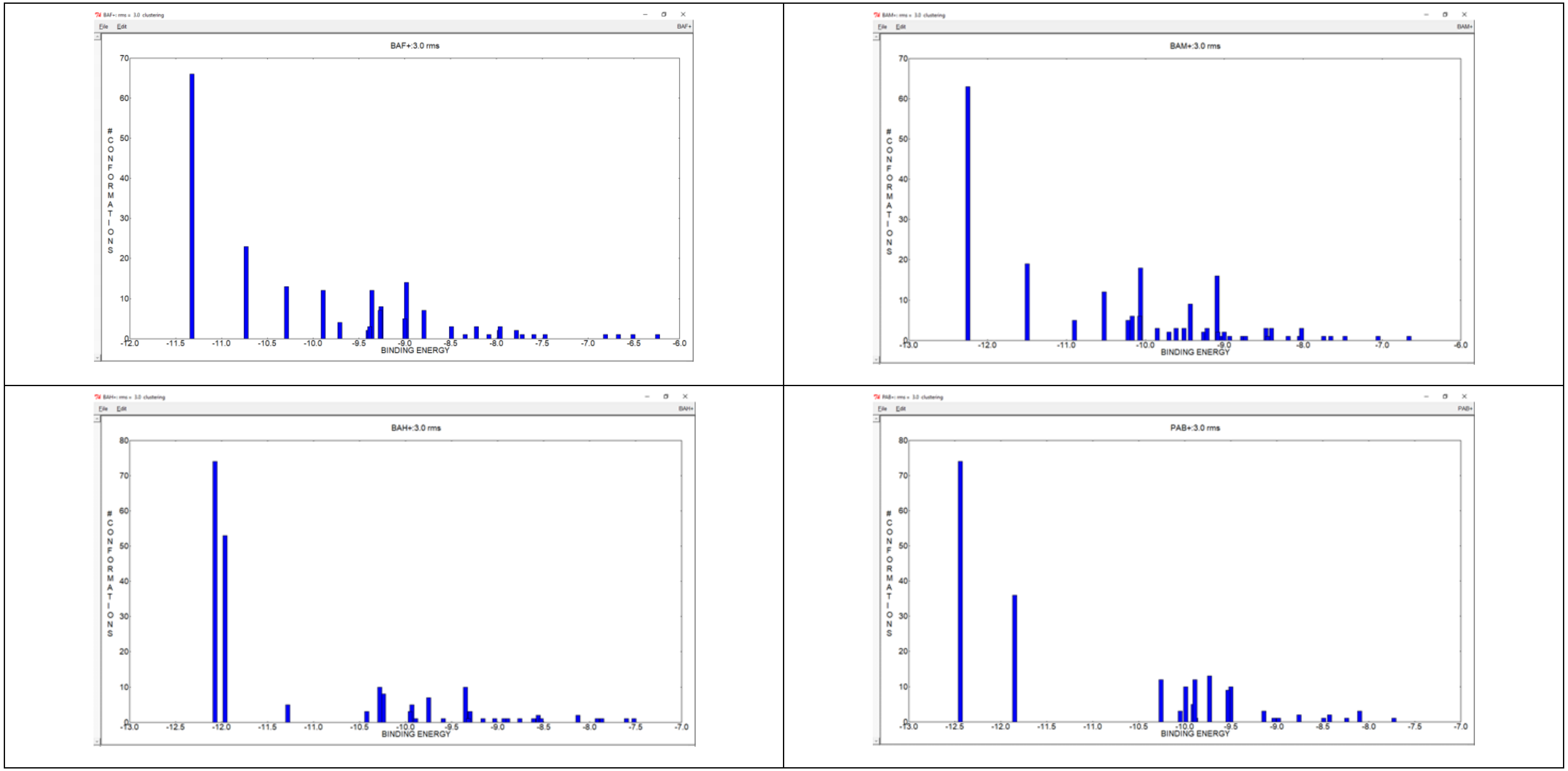

Figure S17. Clustering histogram of the molecular docking simulations for the derivatives **17a**–**17j** in the cleavage complex with *h*Topo IIβ (pdb id: 4G0U).

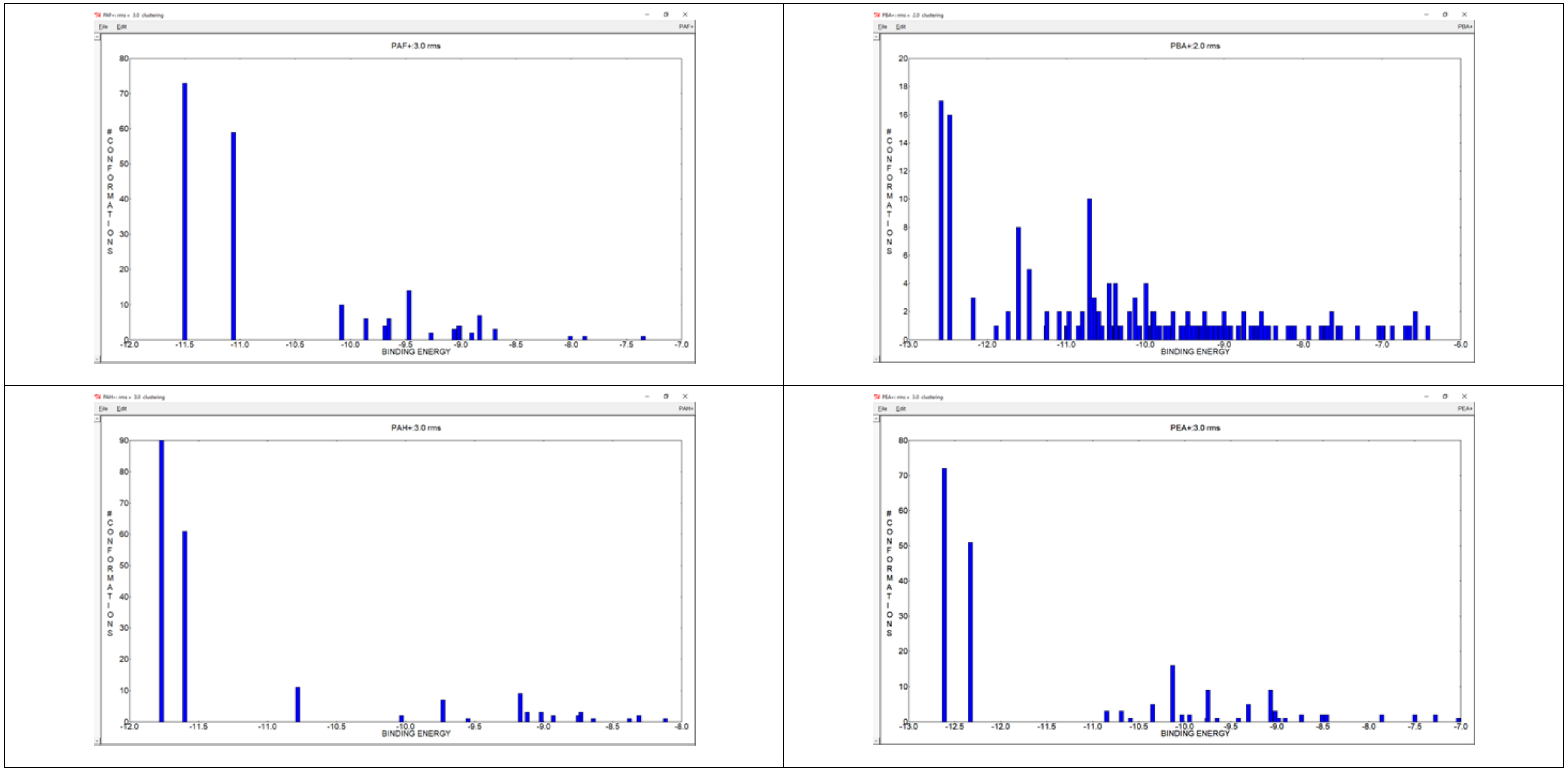

Figure S18. Clustering histogram of the molecular docking simulations for the derivatives **17a**–**17j** in the cleavage complex with *h*Topo IIβ (pdb id: 4G0U).

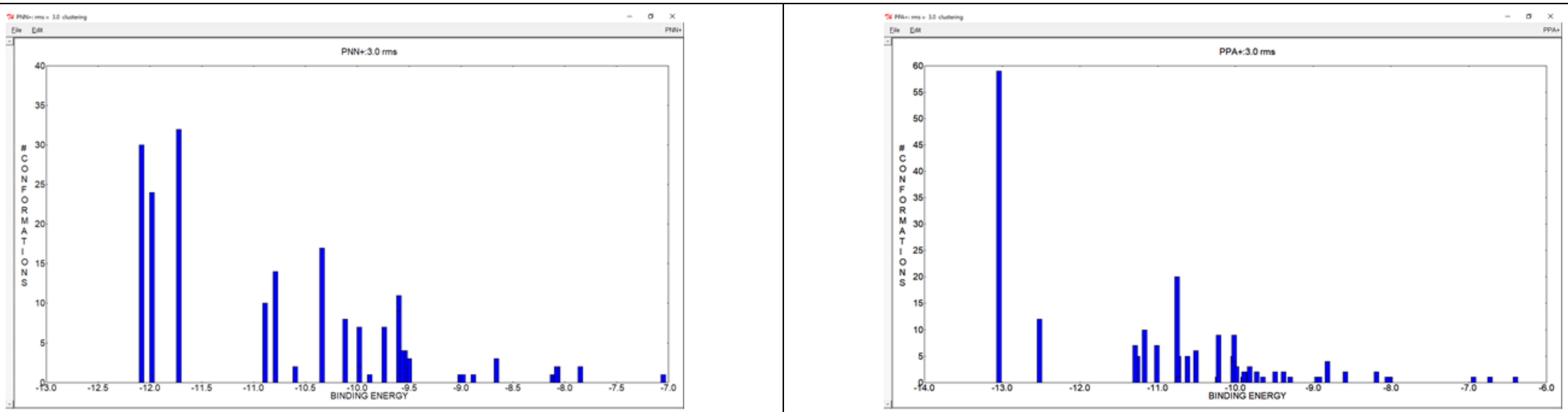

Figure S19. Clustering histogram of the molecular docking simulations for the derivatives **17a**–**17j** in the cleavage complex with *h*Topo IIβ (pdb id: 4G0U).

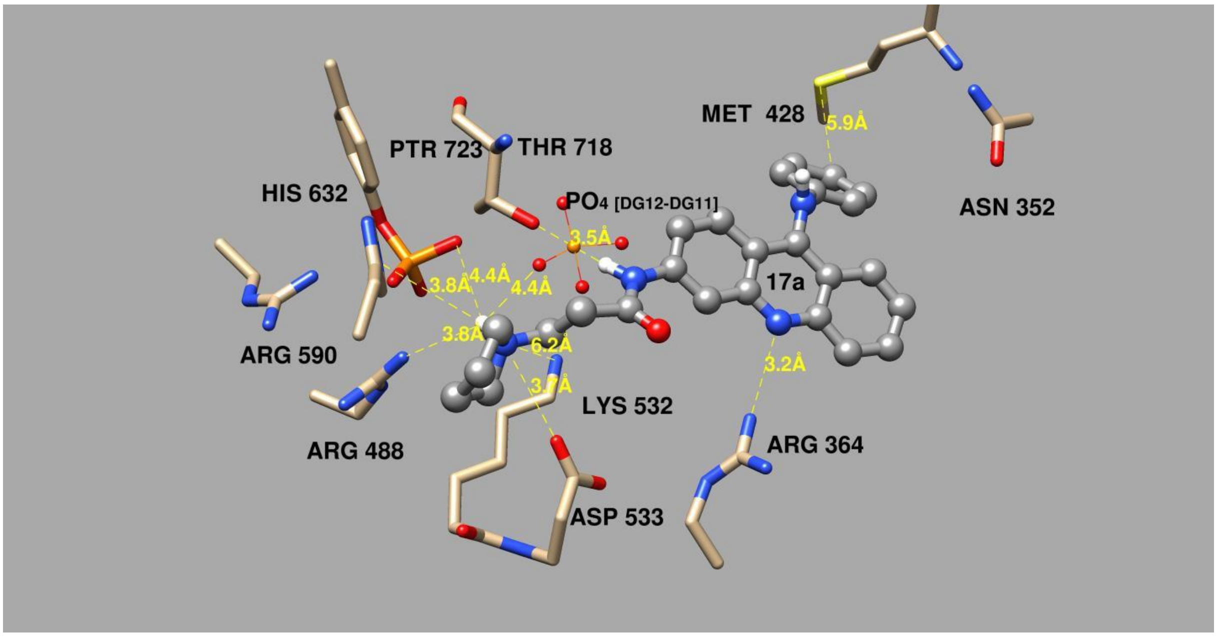

Figure S20. Intermolecular distances between a putative docking pose of the ligand **17a** and amino acid residues of ternary DNA cleavage complex for *h*Topo I (pdb ID: 1T8I) proposed by docking simulations.

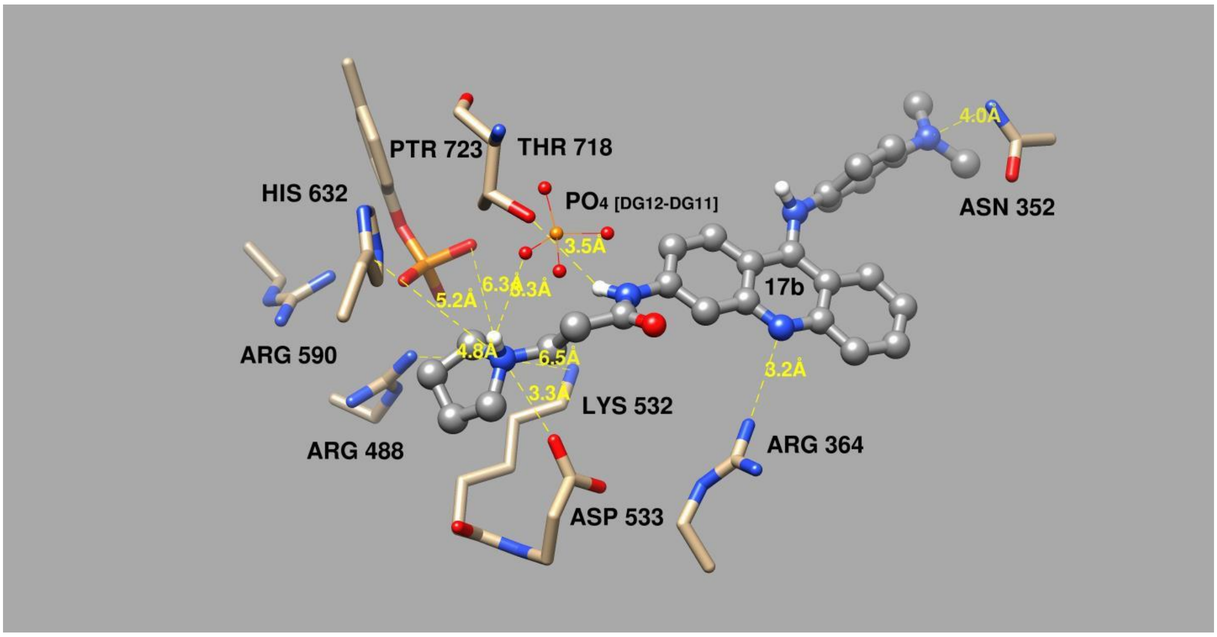

Figure S21. Intermolecular distances between a putative docking pose of the ligand **17b** and amino acid residues of ternary DNA cleavage complex for *h*Topo I (pdb ID: 1T8I) proposed by docking simulations.

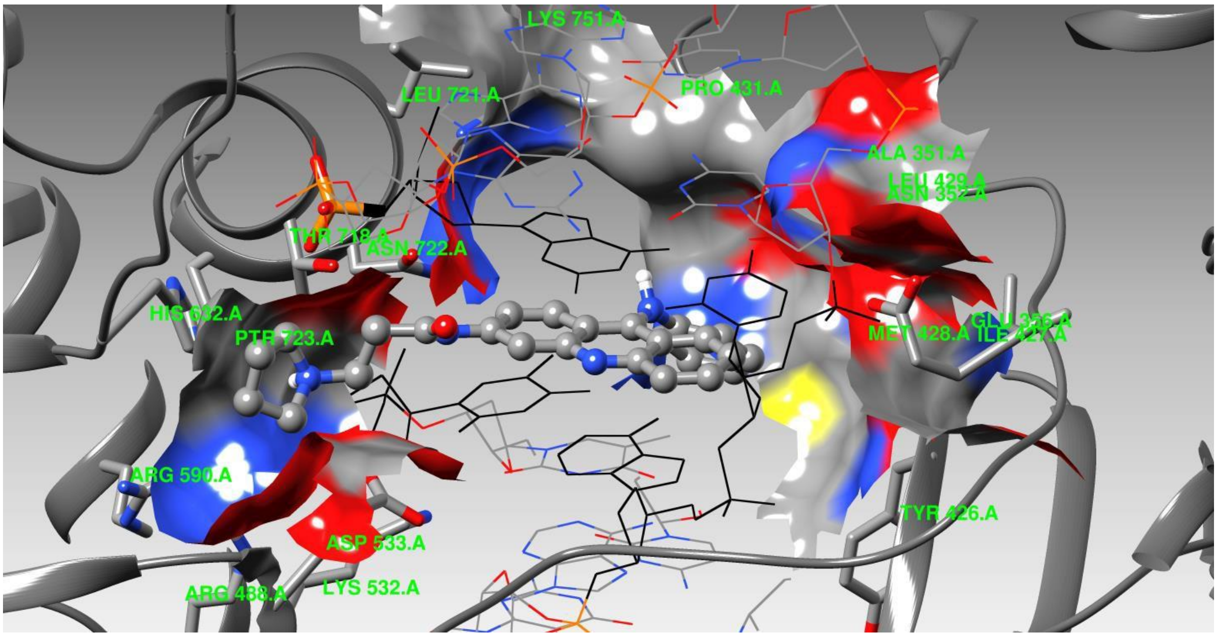

Figure S22. Putative interactions for the acridine derivative **17a** with the enzyme surface of *h*Topo I (pdb ID: 1T8I) proposed by docking simulations.

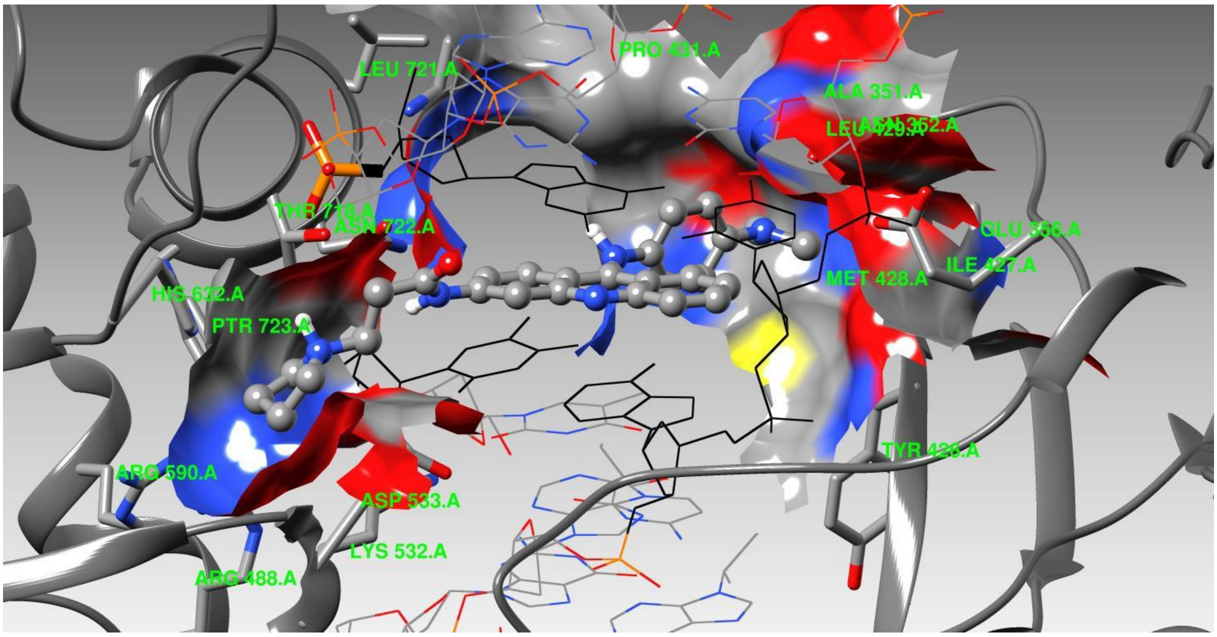

Figure S23. Putative interactions for the acridine derivative **17b** with the enzyme surface of *h*Topo I (pdb ID: 1T8I) proposed by docking simulations.

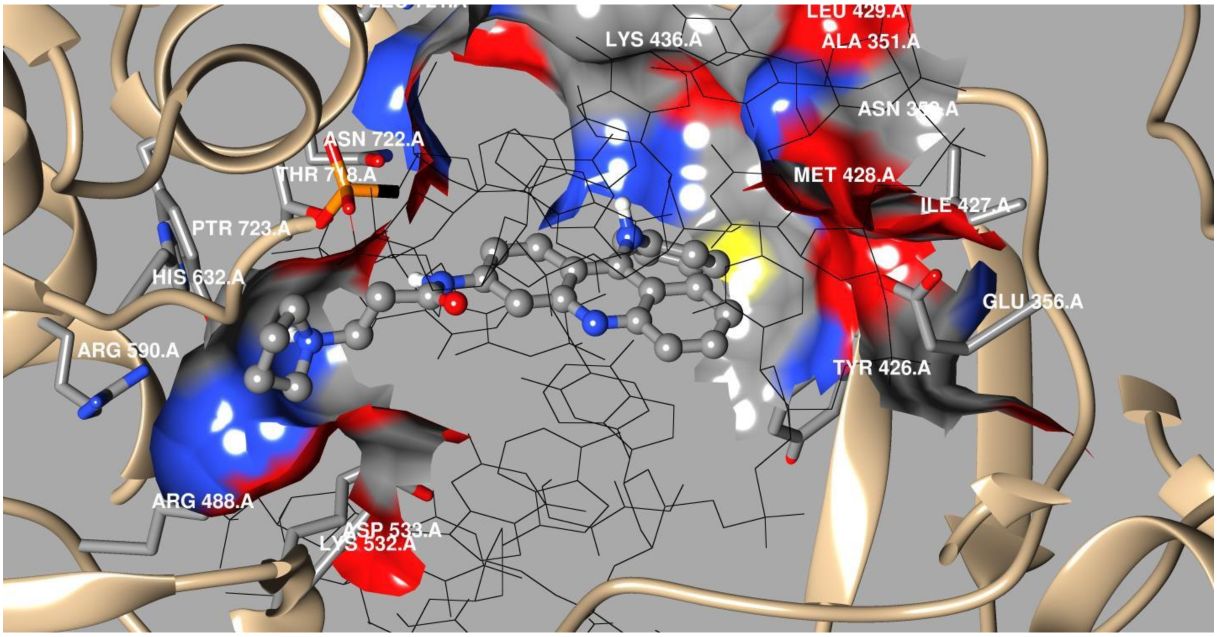

Figure S24. Putative interactions for the acridine derivative **17a** with the enzyme surface of *h*Topo I (pdb ID: 1T8I) proposed by docking simulations.

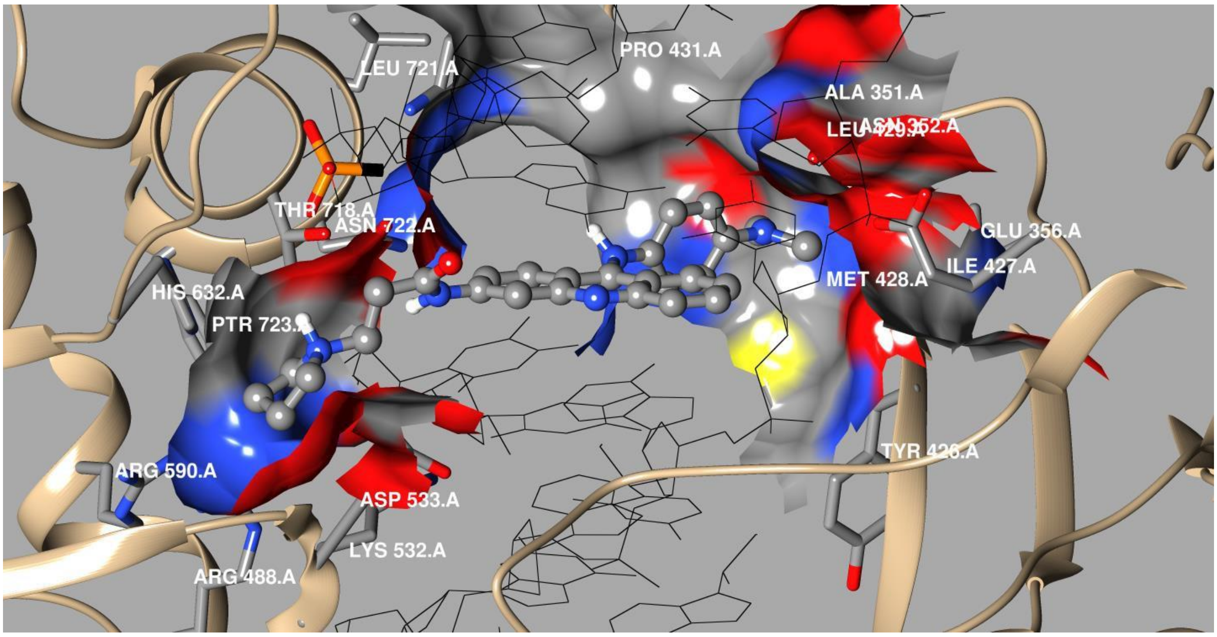

Figure S25. Putative interactions for the acridine derivative **17b** with the enzyme surface of *h*Topo I (pdb ID: 1T8I) proposed by docking simulations.

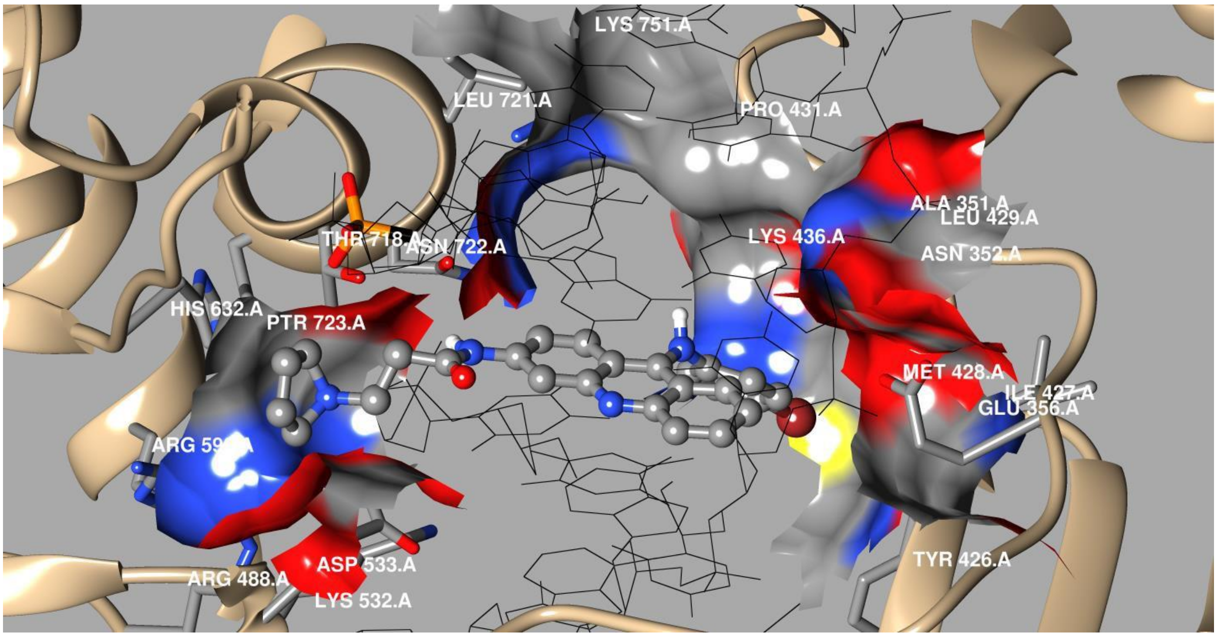

Figure S26. Putative interactions for the acridine derivative **17c** with the enzyme surface of *h*Topo I (pdb ID: 1T8I) proposed by docking simulations.

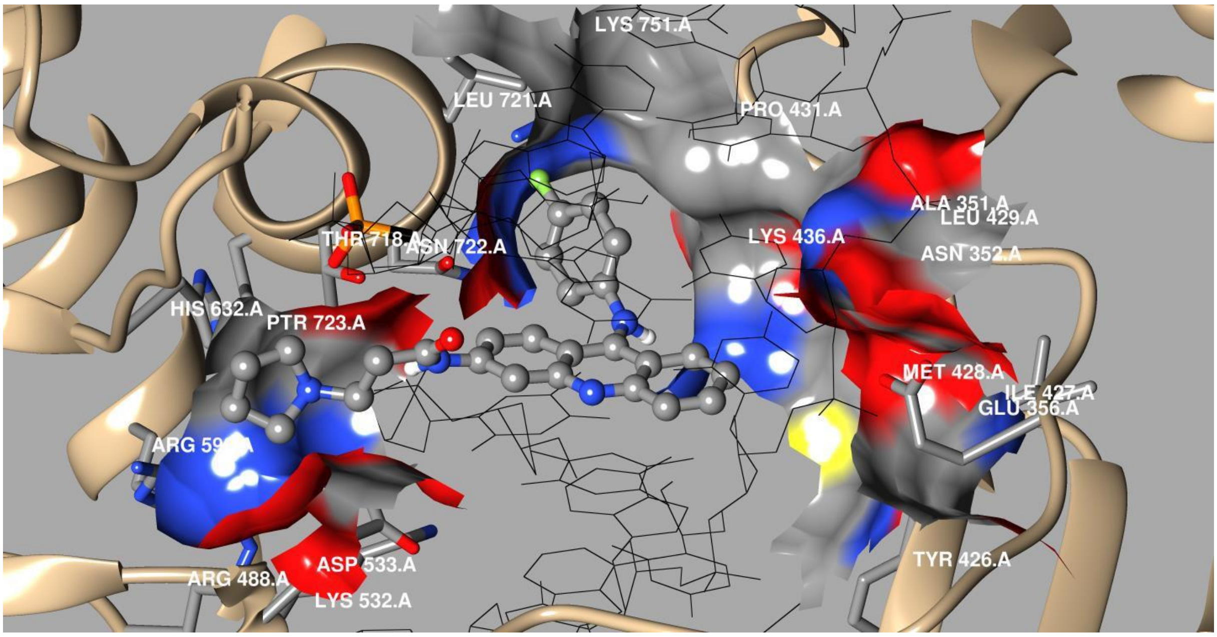

Figure S27. Putative interactions for the acridine derivative **17d** with the enzyme surface of *h*Topo I (pdb ID: 1T8I) proposed by docking simulations.

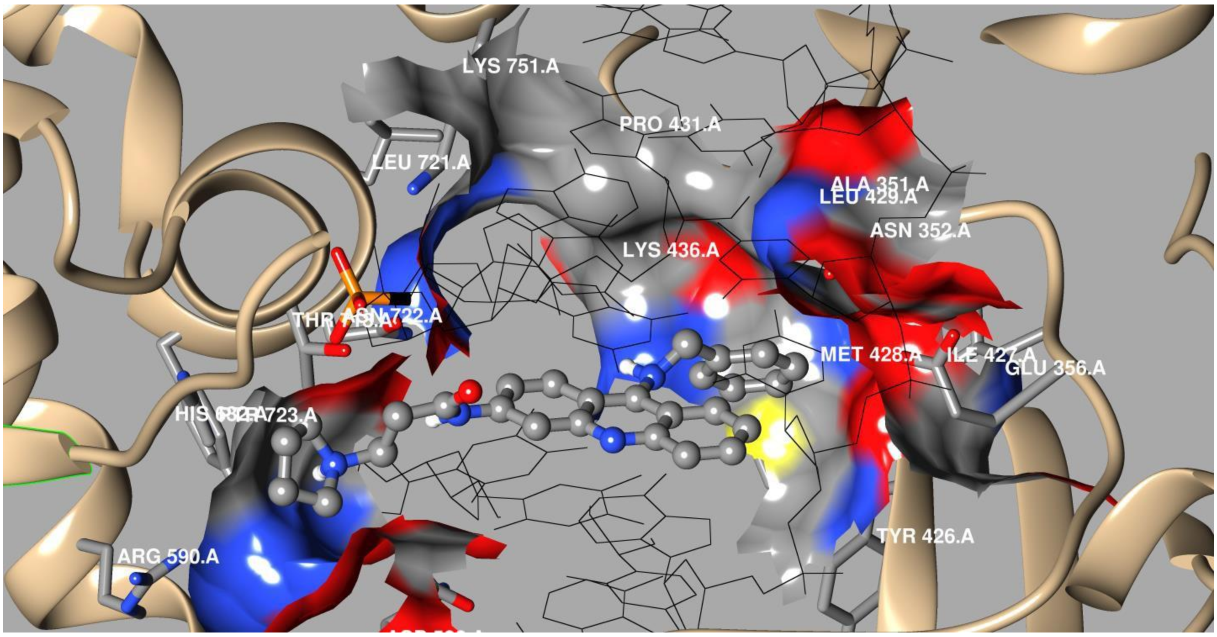

Figure S28. Putative interactions for the acridine derivative **17e** with the enzyme surface of *h*Topo I (pdb ID: 1T8I) proposed by docking simulations.

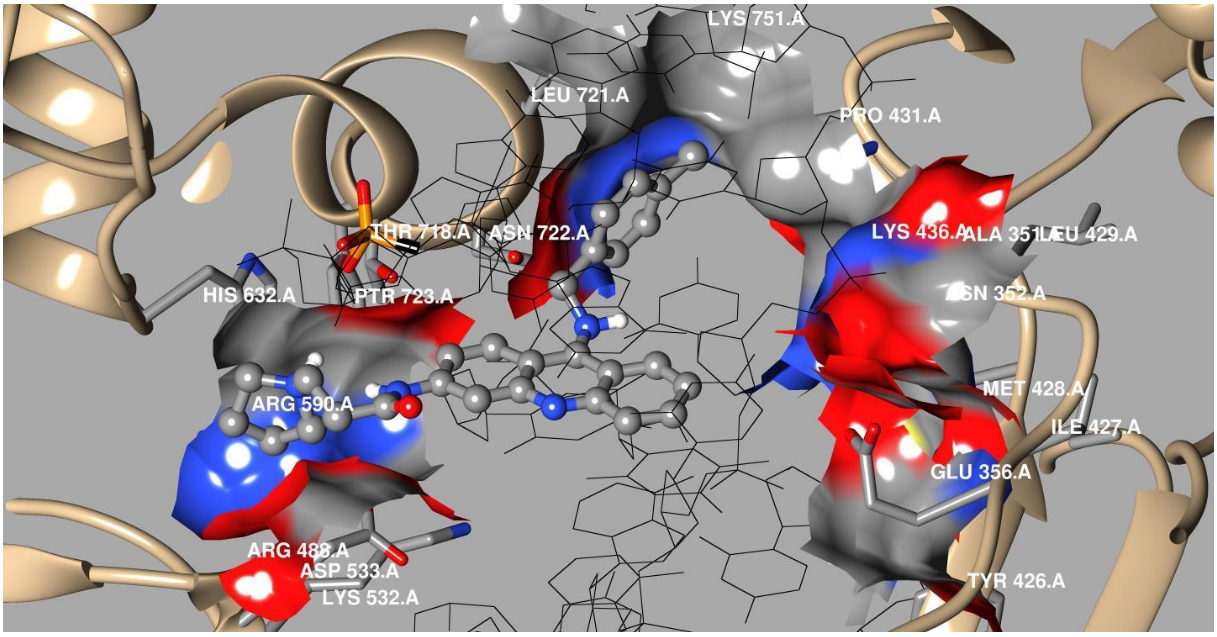

Figure S29. Putative interactions for the acridine derivative **17f** with the enzyme surface of *h*Topo I (pdb ID: 1T8I) proposed by docking simulations.

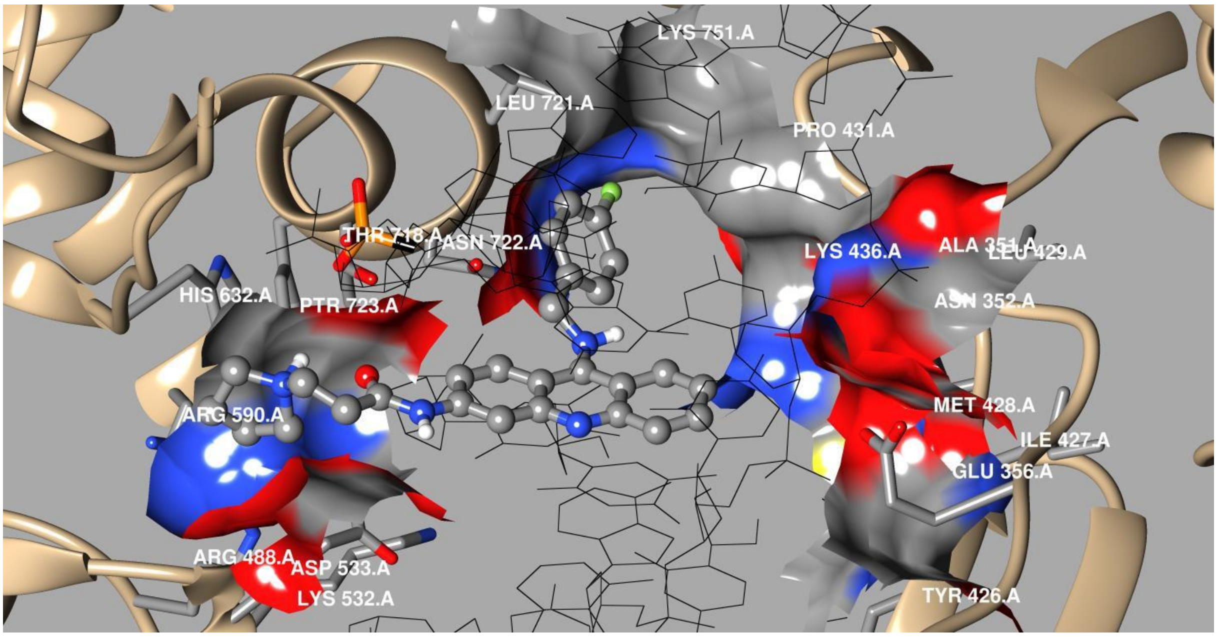

Figure S30. Putative interactions for the acridine derivative **17g** with the enzyme surface of *h*Topo I (pdb ID: 1T8I) proposed by docking simulations.

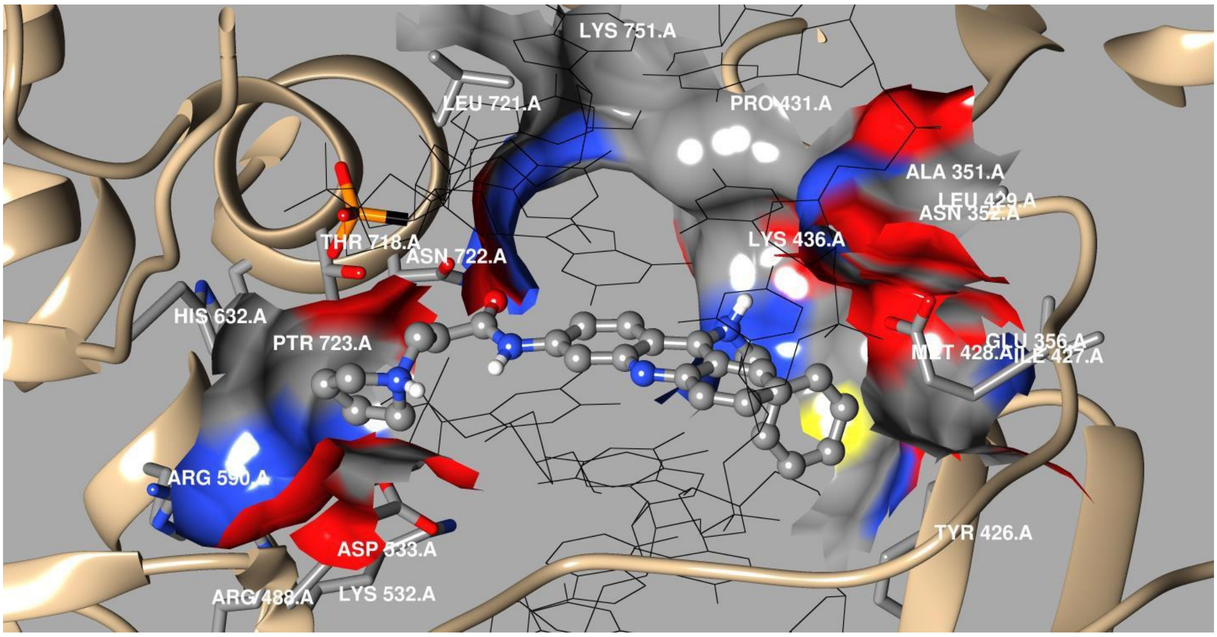

Figure S31. Putative interactions for the acridine derivative **17h** with the enzyme surface of *h*Topo I (pdb ID: 1T8I) proposed by docking simulations.

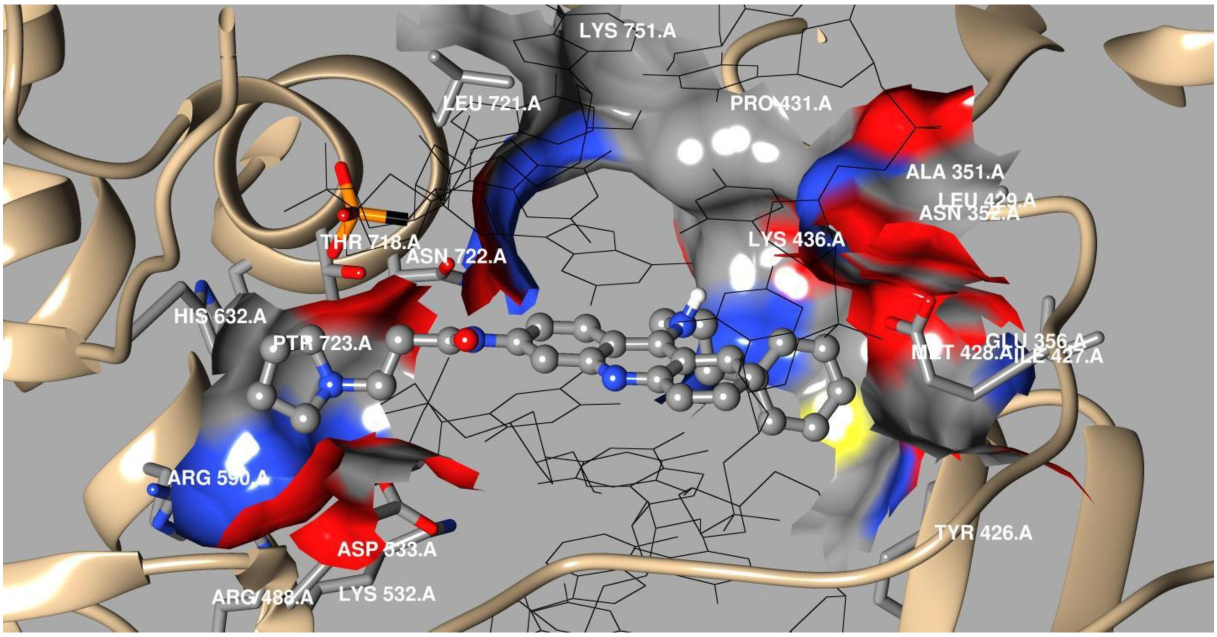

Figure S32. Putative interactions for the acridine derivative **17i** with the enzyme surface of *h*Topo I (pdb ID: 1T8I) proposed by docking simulations.

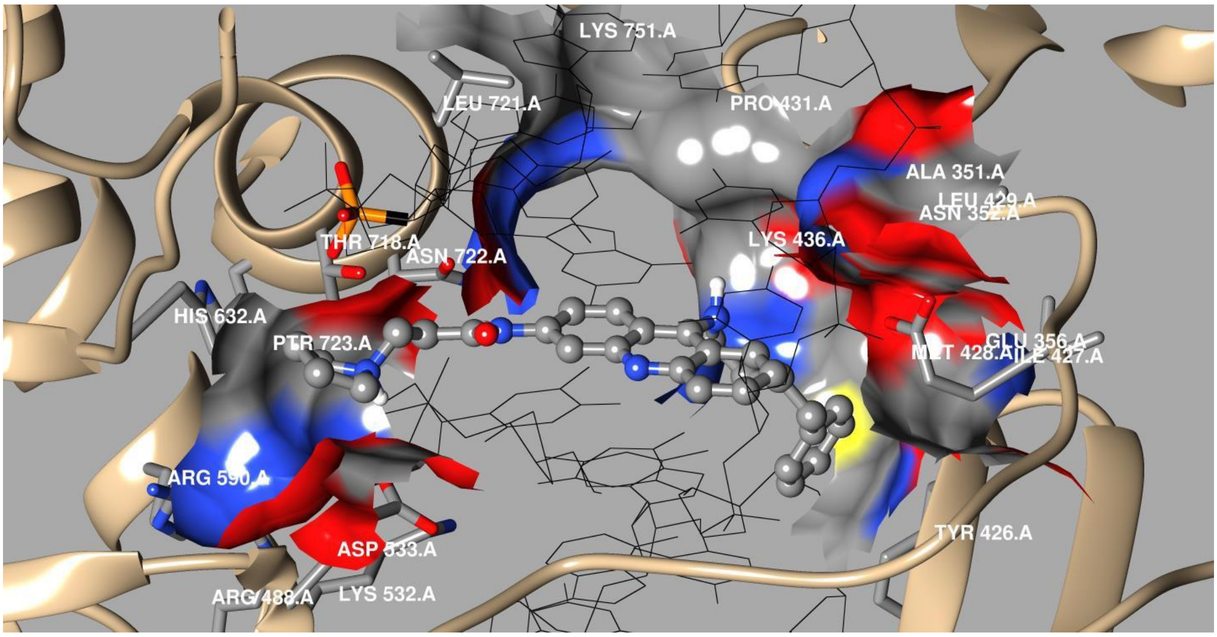

Figure S33. Putative interactions for the acridine derivative **17j** with the enzyme surface of *h*Topo I (pdb ID: 1T8I) proposed by docking simulations.

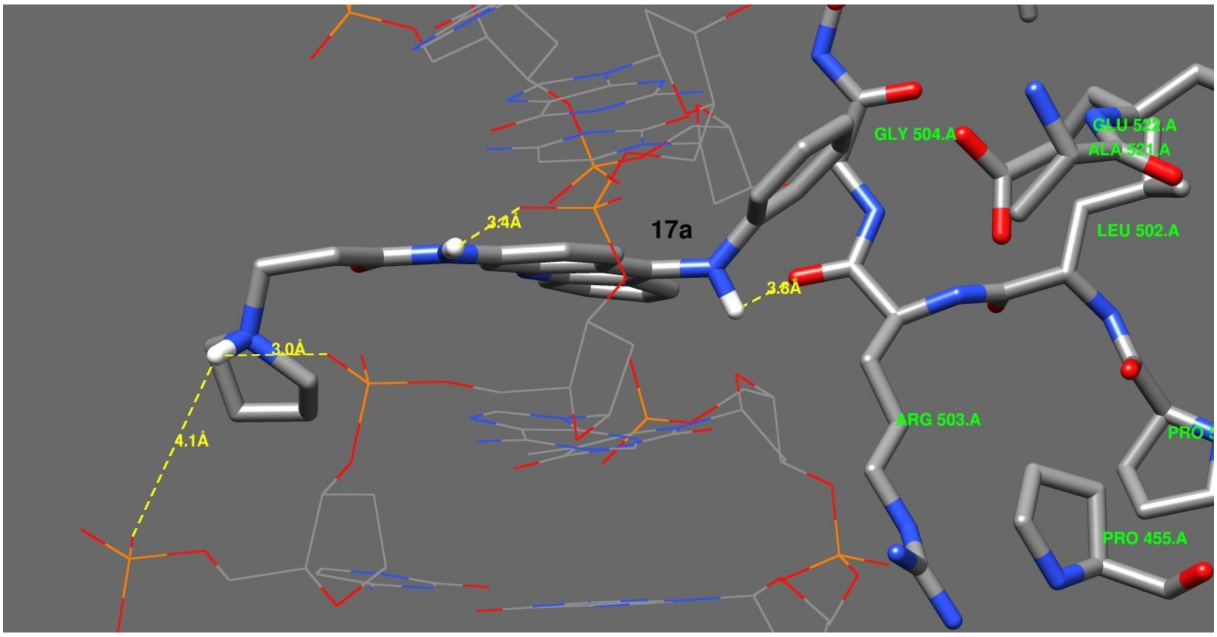

Figure S34. Intermolecular distances between a putative docking pose of the ligand **17a** and amino acid residues of ternary DNA cleavage complex for *h*Topo IIβ (pdb ID: 4G0U) proposed by docking simulations.

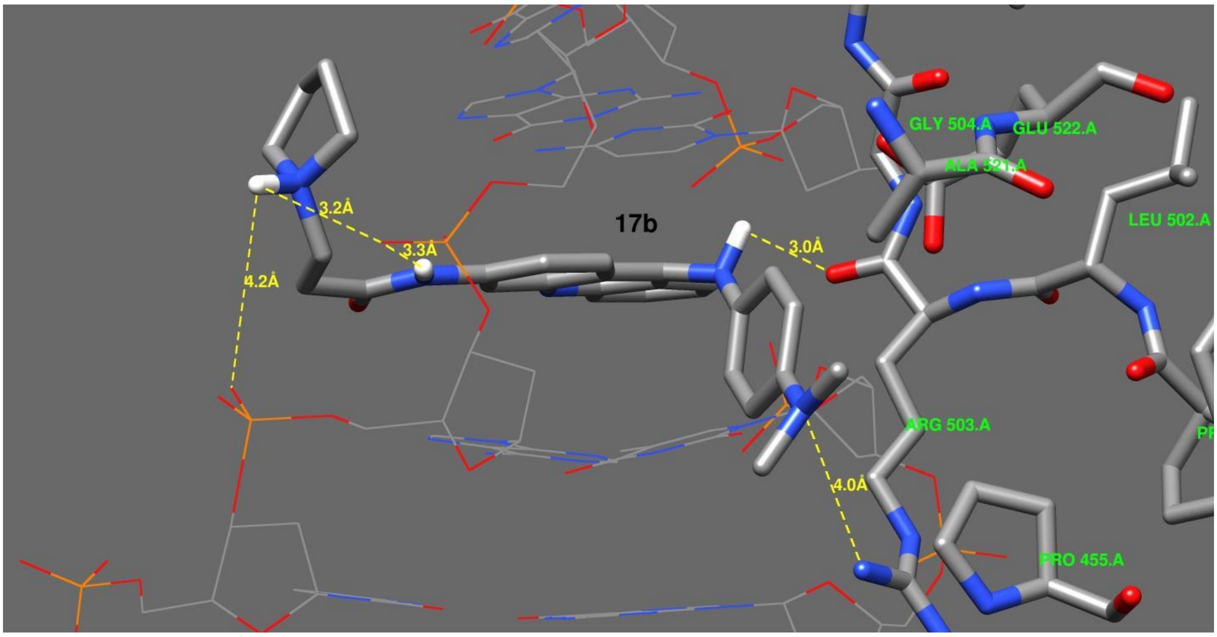

Figure S35. Intermolecular distances between a putative docking pose of the ligand **17b** and amino acid residues of ternary DNA cleavage complex for *h*Topo IIβ (pdb ID: 4G0U) proposed by docking simulations.

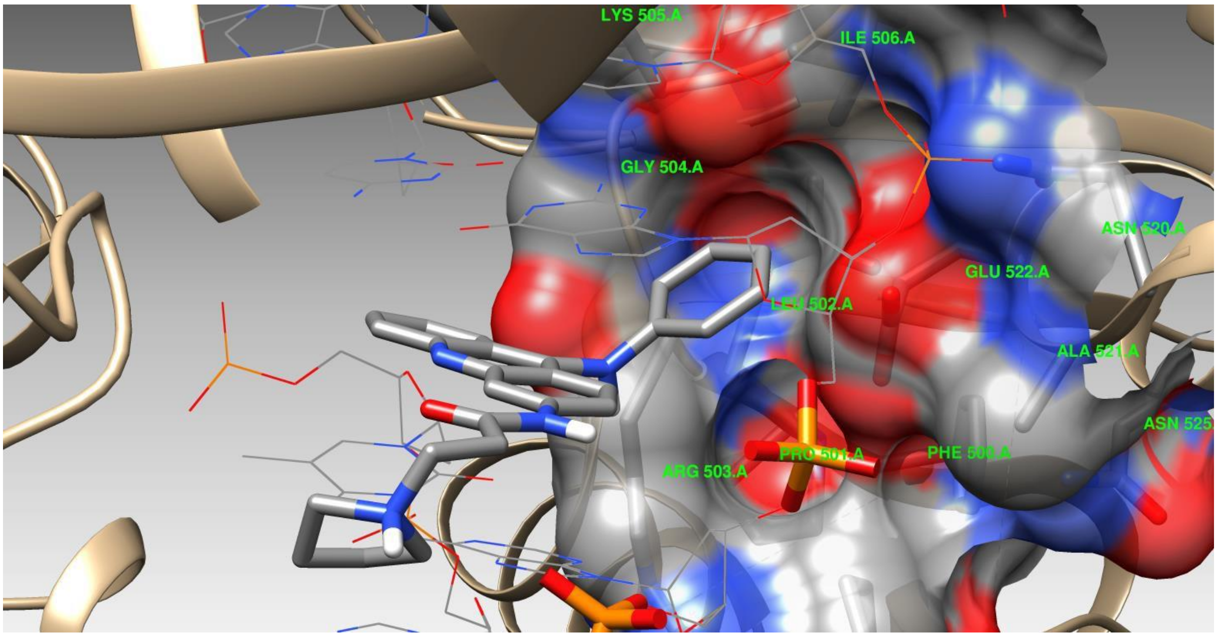

Figure S36. Putative interactions for the acridine derivative **17a** with the enzyme surface of *h*Topo IIβ (pdb ID: 4G0U) proposed by docking simulations.

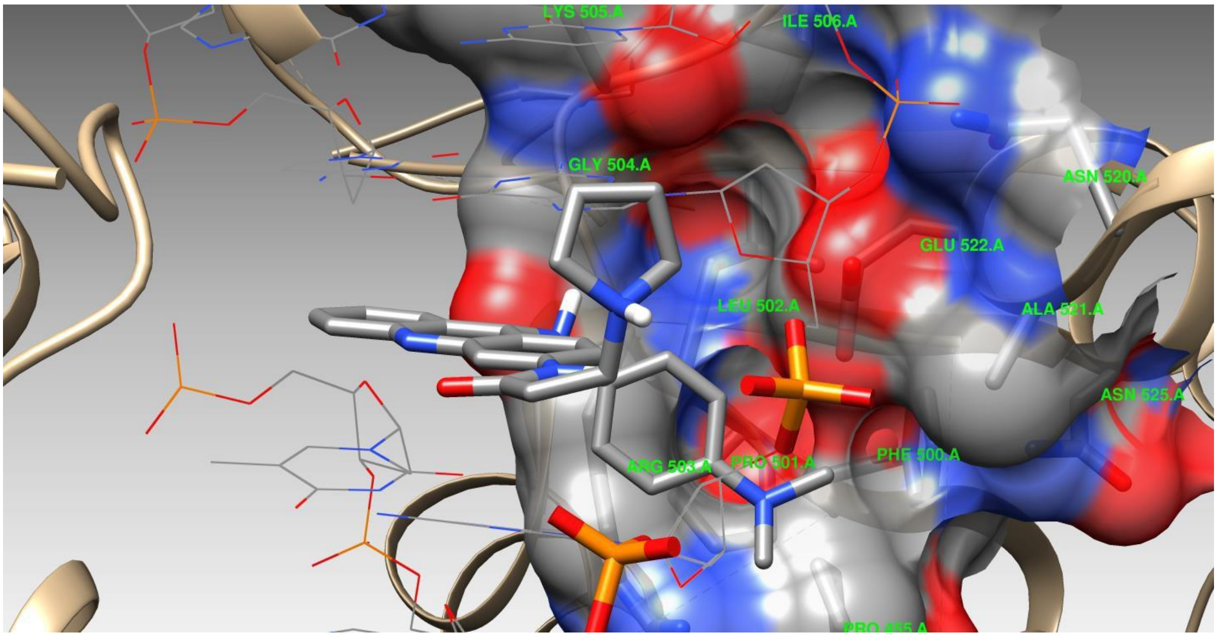

Figure S37. Putative interactions for the acridine derivative **17b** with the enzyme surface of *h*Topo IIβ (pdb ID: 4G0U) proposed by docking simulations.

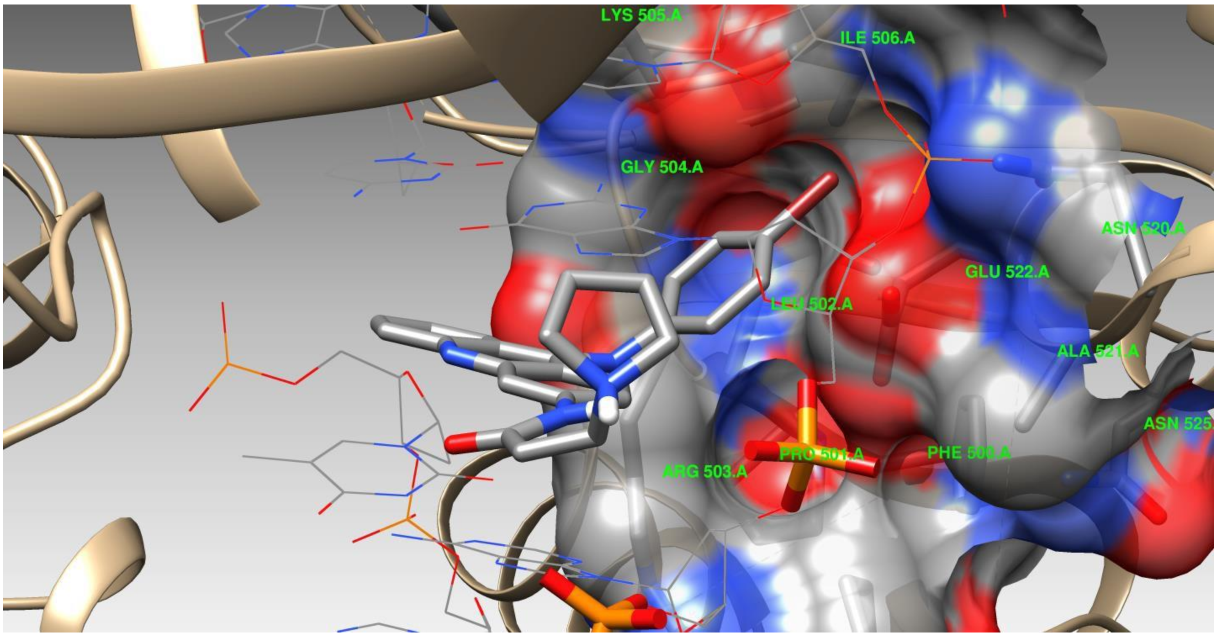

Figure S38. Putative interactions for the acridine derivative **17c** with the enzyme surface of *h*Topo IIβ (pdb ID: 4G0U) proposed by docking simulations.

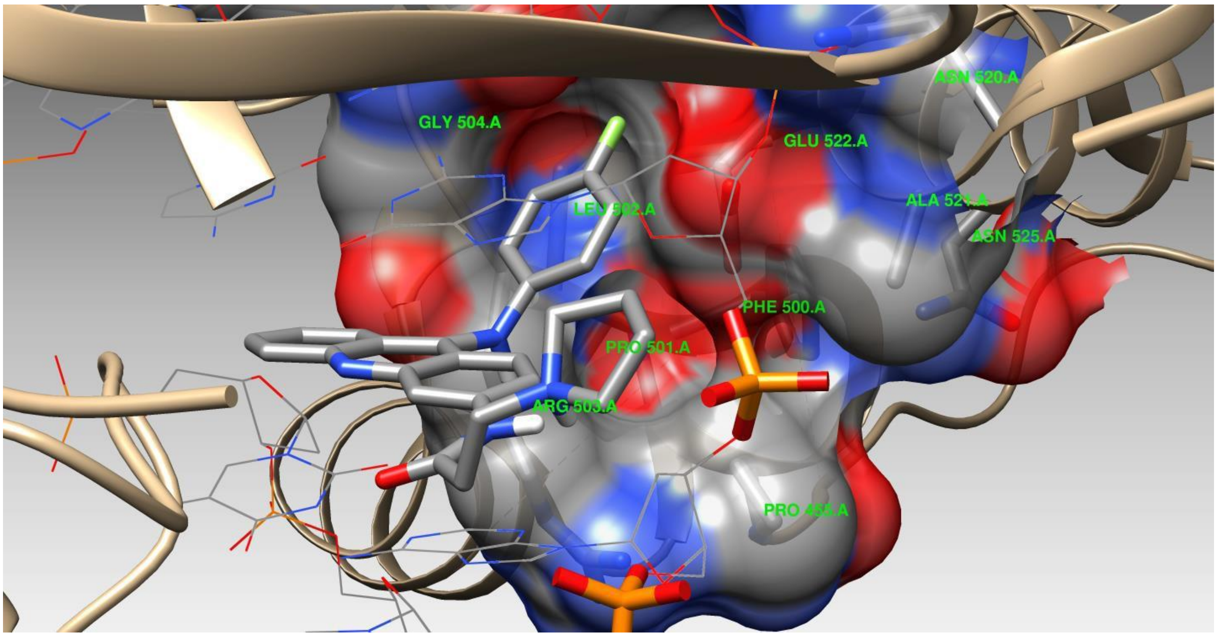

Figure S39. Putative interactions for the acridine derivative **17d** with the enzyme surface of *h*Topo IIβ (pdb ID: 4G0U) proposed by docking simulations.

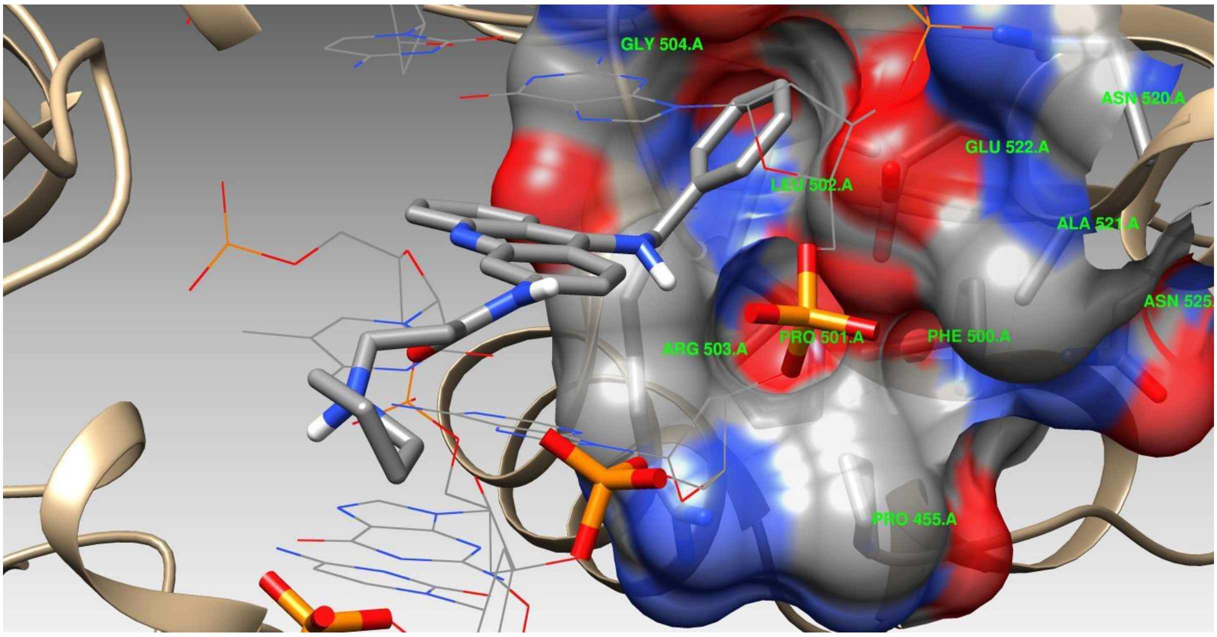

Figure S40. Putative interactions for the acridine derivative **17e** with the enzyme surface of *h*Topo IIβ (pdb ID: 4G0U) proposed by docking simulations.

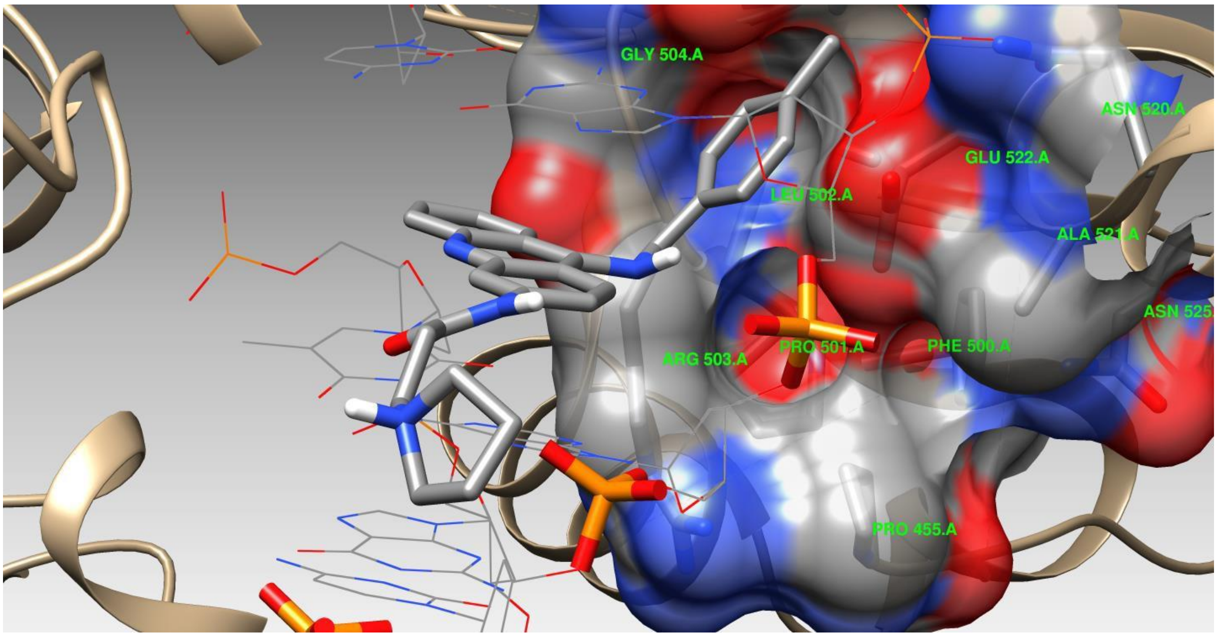

Figure S41. Putative interactions for the acridine derivative **17f** with the enzyme surface of *h*Topo IIβ (pdb ID: 4G0U) proposed by docking simulations.

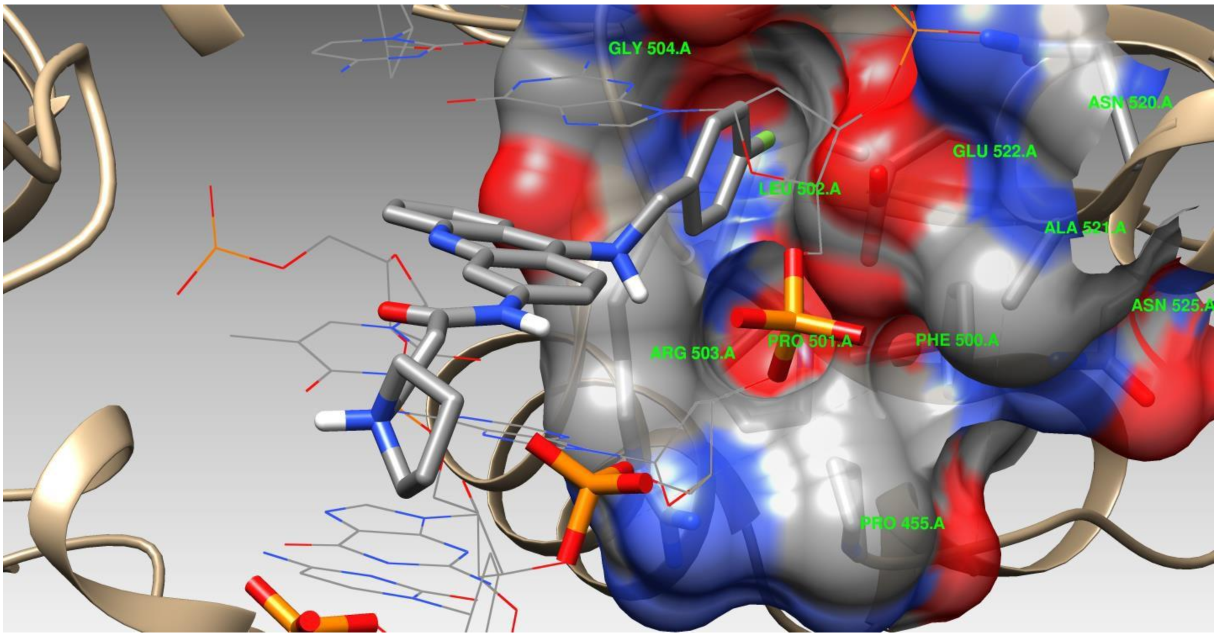

Figure S42. Putative interactions for the acridine derivative **17g** with the enzyme surface of *h*Topo IIβ (pdb ID: 4G0U) proposed by docking simulations.

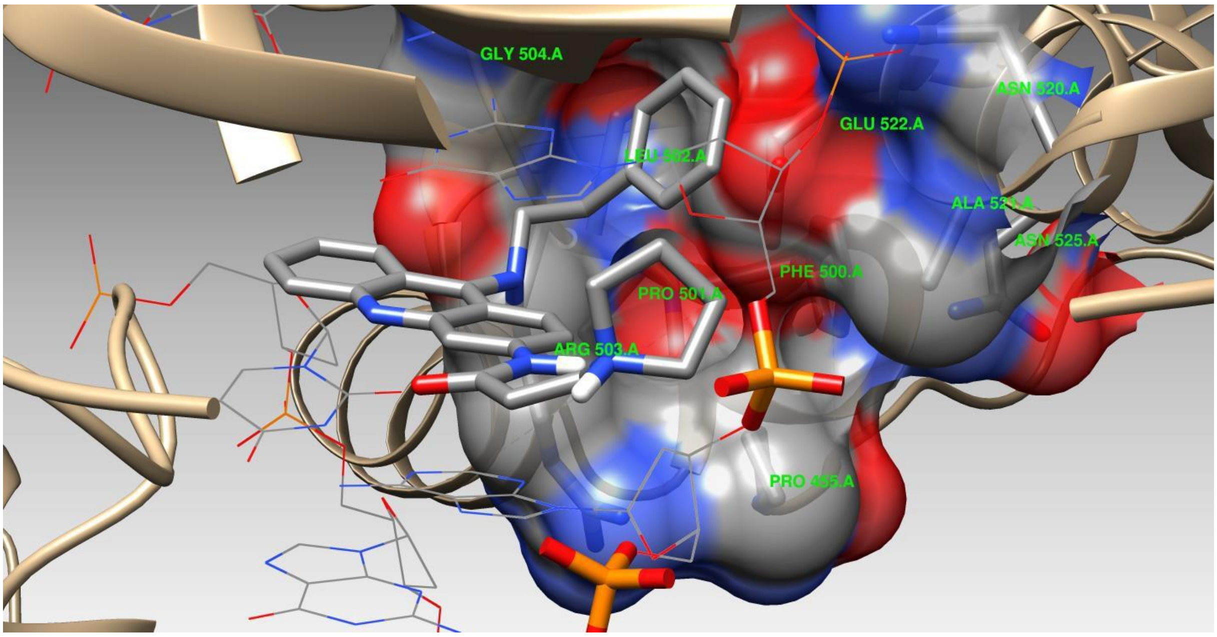

Figure S43. Putative interactions for the acridine derivative **17h** with the enzyme surface of *h*Topo IIβ (pdb ID: 4G0U) proposed by docking simulations.

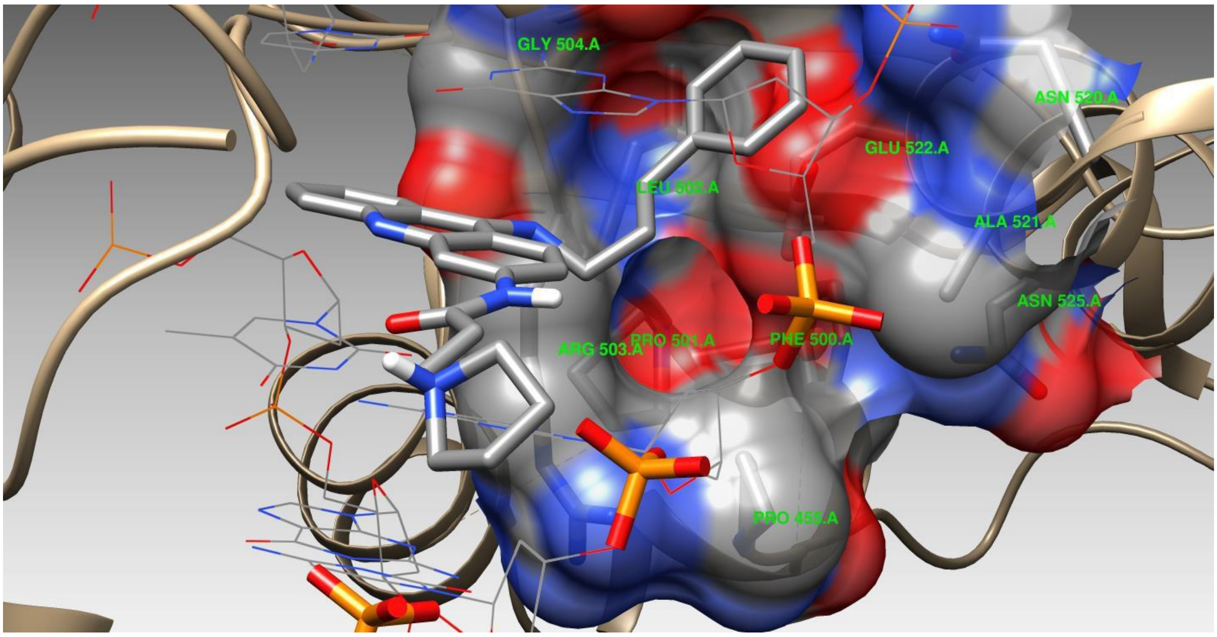

Figure S44. Putative interactions for the acridine derivative **17i** with the enzyme surface of *h*Topo IIβ (pdb ID: 4G0U) proposed by docking simulations.

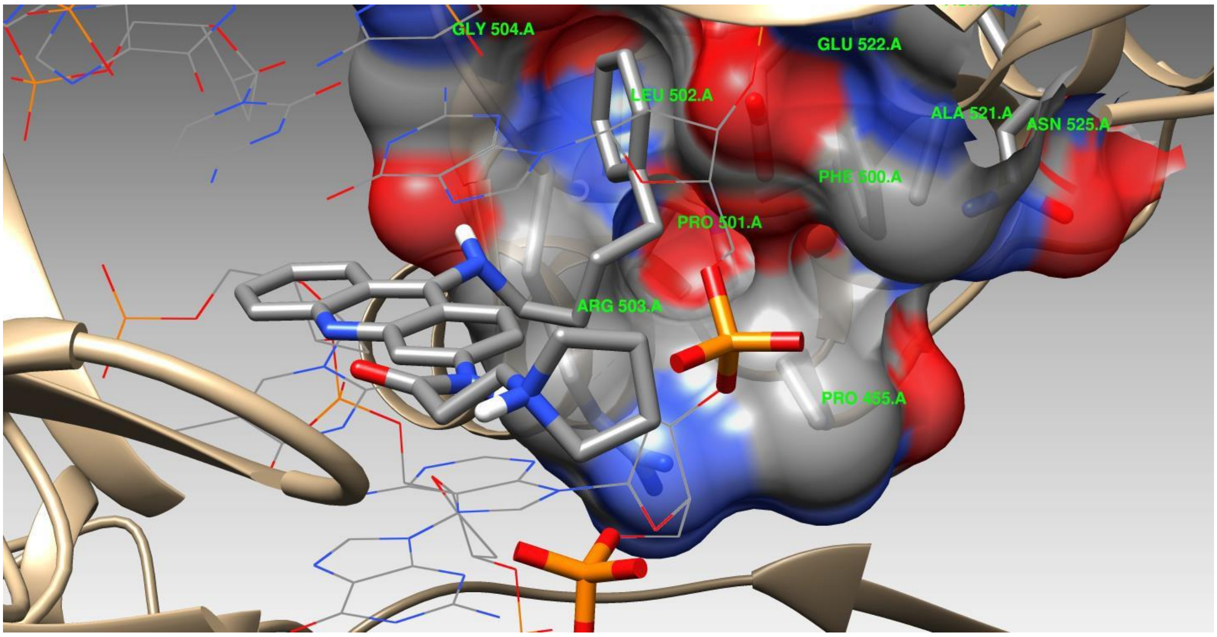

Figure S45. Putative interactions for the acridine derivative **17j** with the enzyme surface of *h*Topo IIβ (pdb ID: 4G0U) proposed by docking simulations.

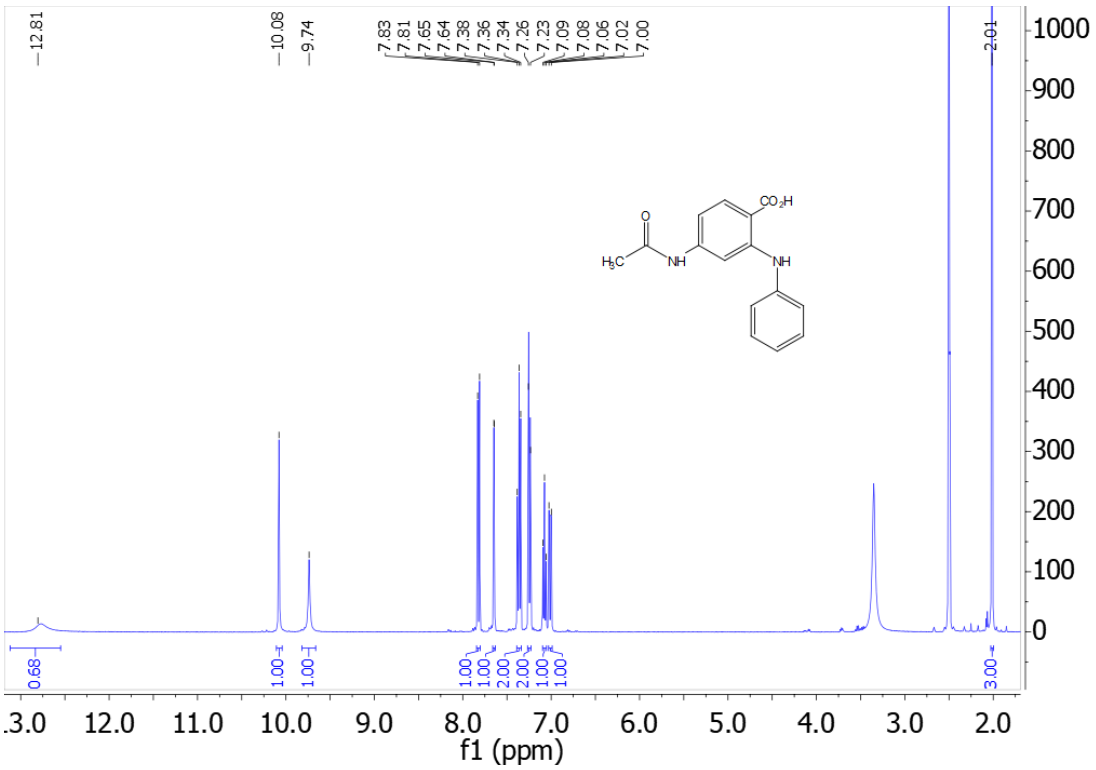

Figure S46. ^{1}H NMR spectra (DMSO-d_6, 600 MHz) of *N*-phenylanthranilic acid **12**.

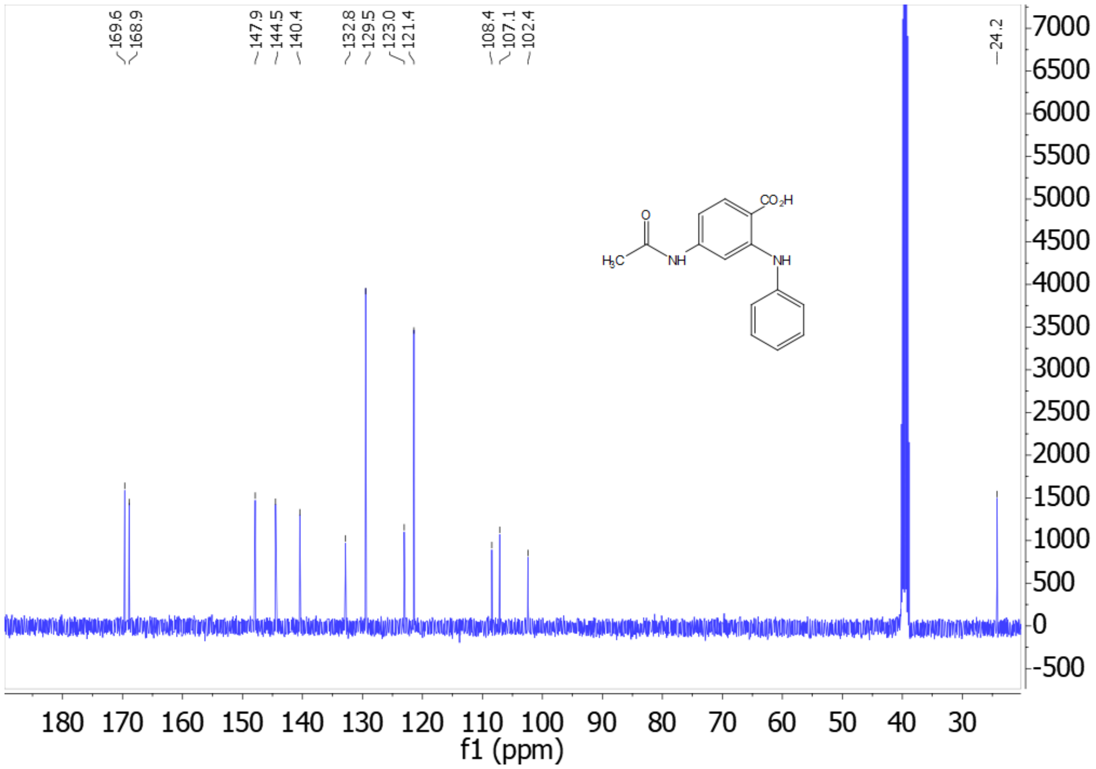

Figure S47. ^{13}C NMR spectra (DMSO-d_6, 150 MHz) of *N*-phenylanthranilic acid **12**.

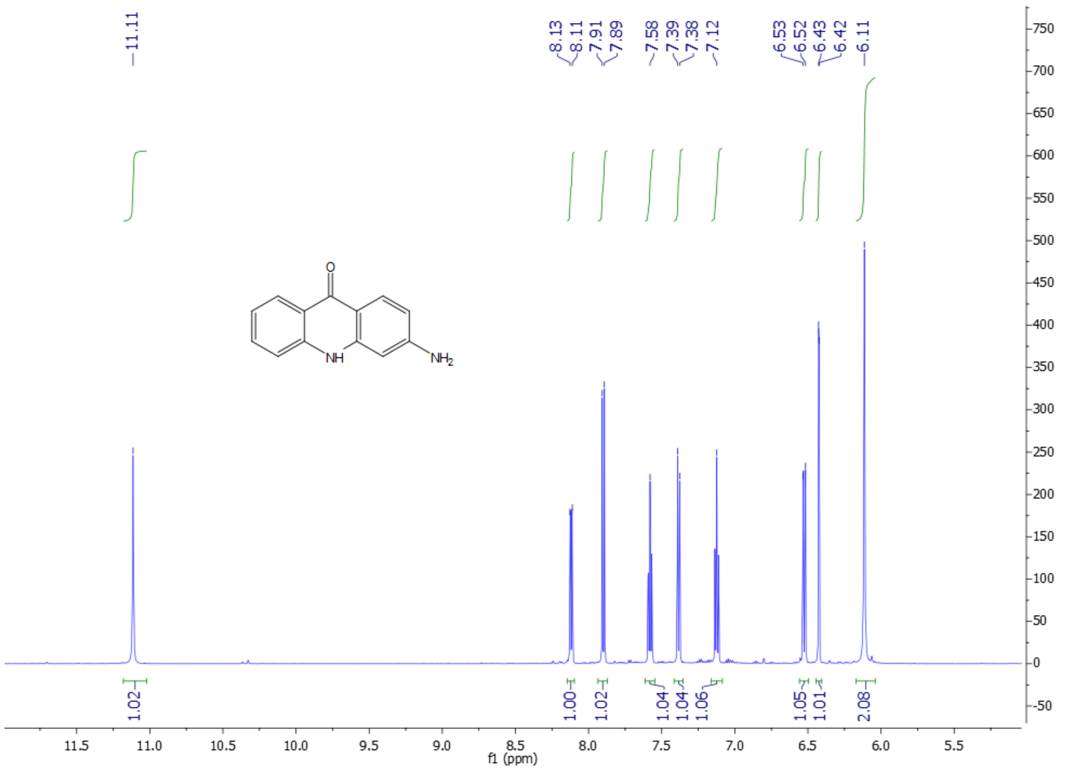

Figure S48. ^{1}H NMR spectra (DMSO-d_6, 600 MHz) of acridine **13**.

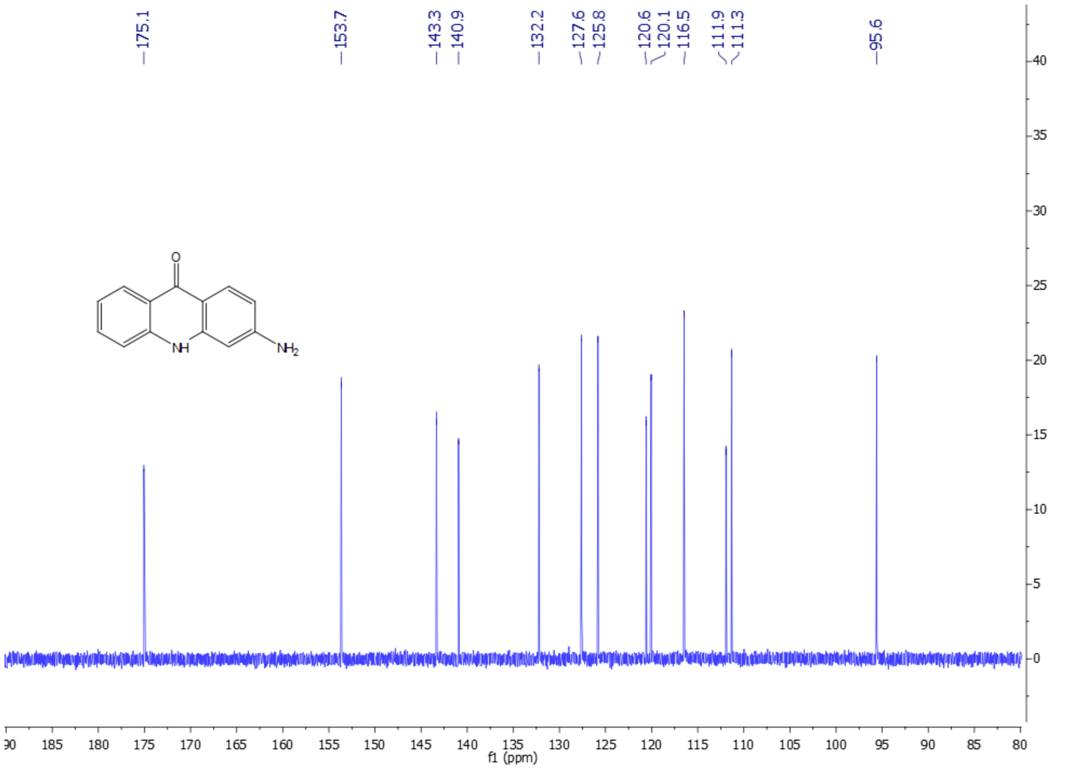

Figure S49. ^{13}C NMR spectra (DMSO-d_6, 150 MHz) of acridine **13**.

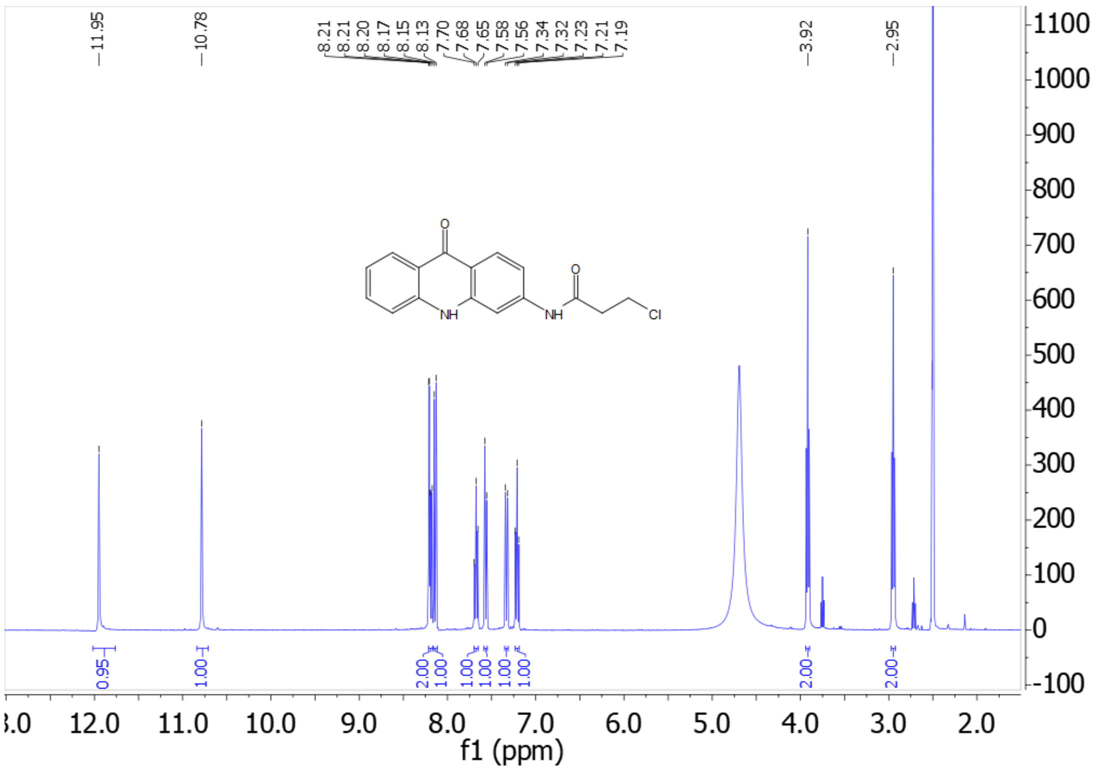

Figure S50. ^{1}H NMR spectra (DMSO-d_6, 600 MHz) of acridine derivative **14**.

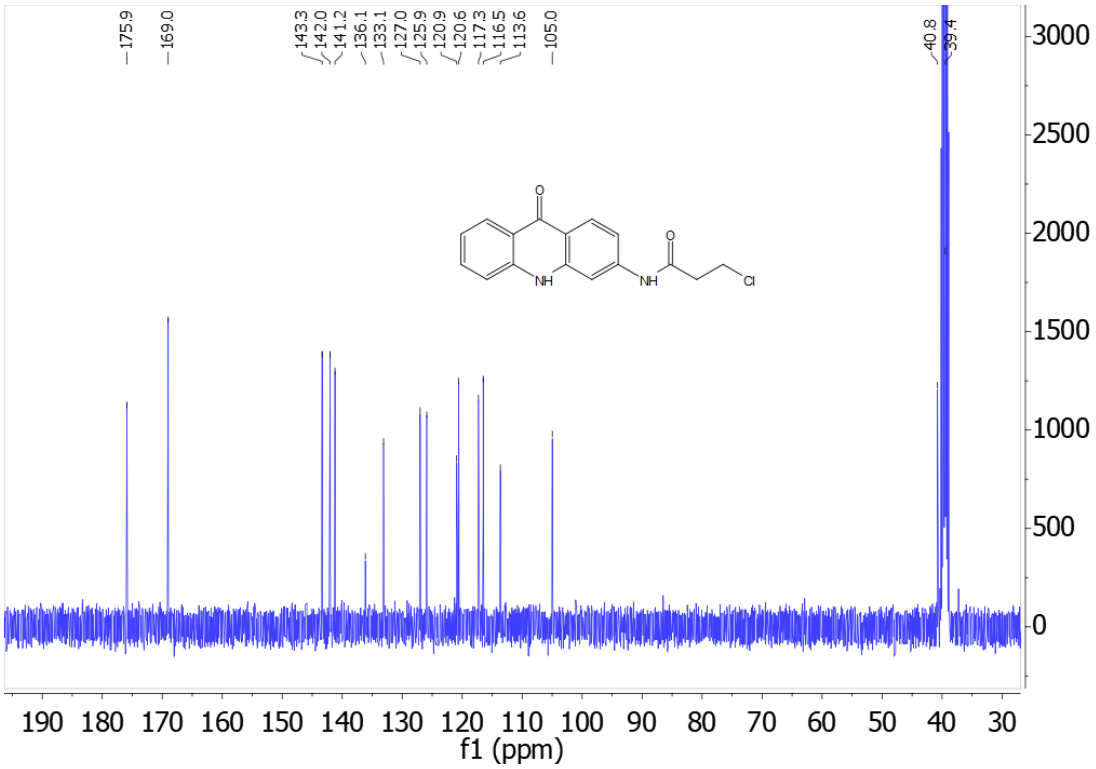

Figure S51. ^{13}C NMR spectra (DMSO-d_6, 150 MHz) of acridine derivative **14**.

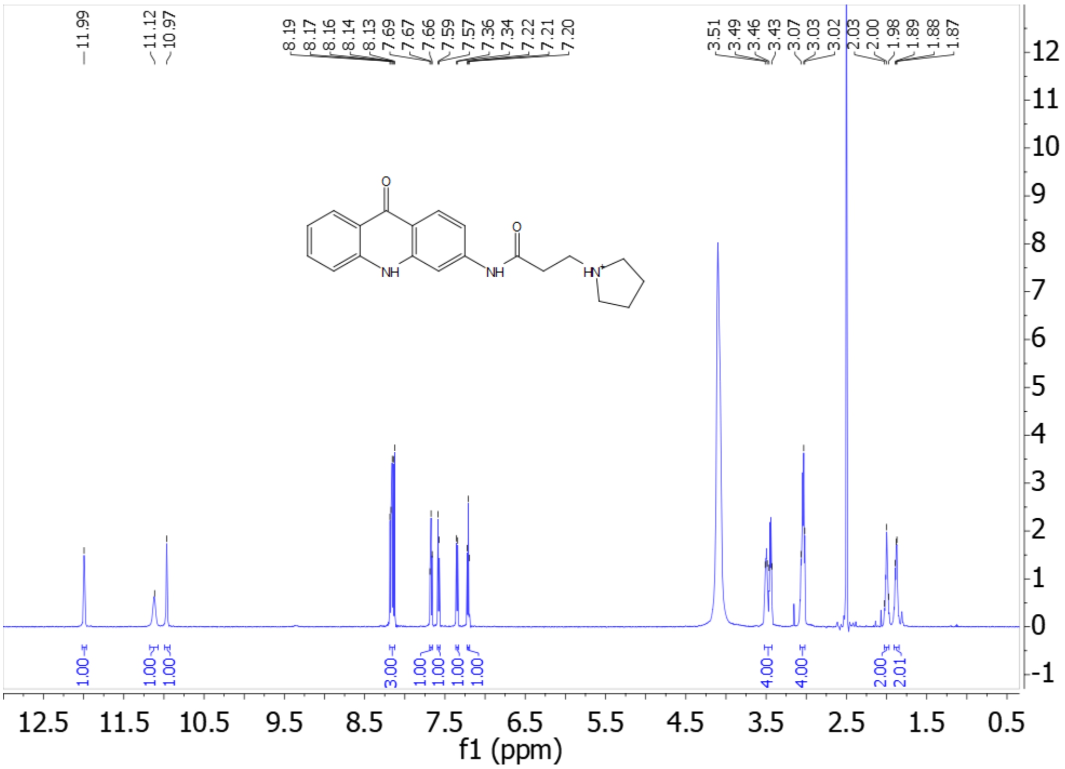

Figure S52. ^{1}H NMR spectra (DMSO-d_6, 600 MHz) of the acridine derivative **15** HCl salt.

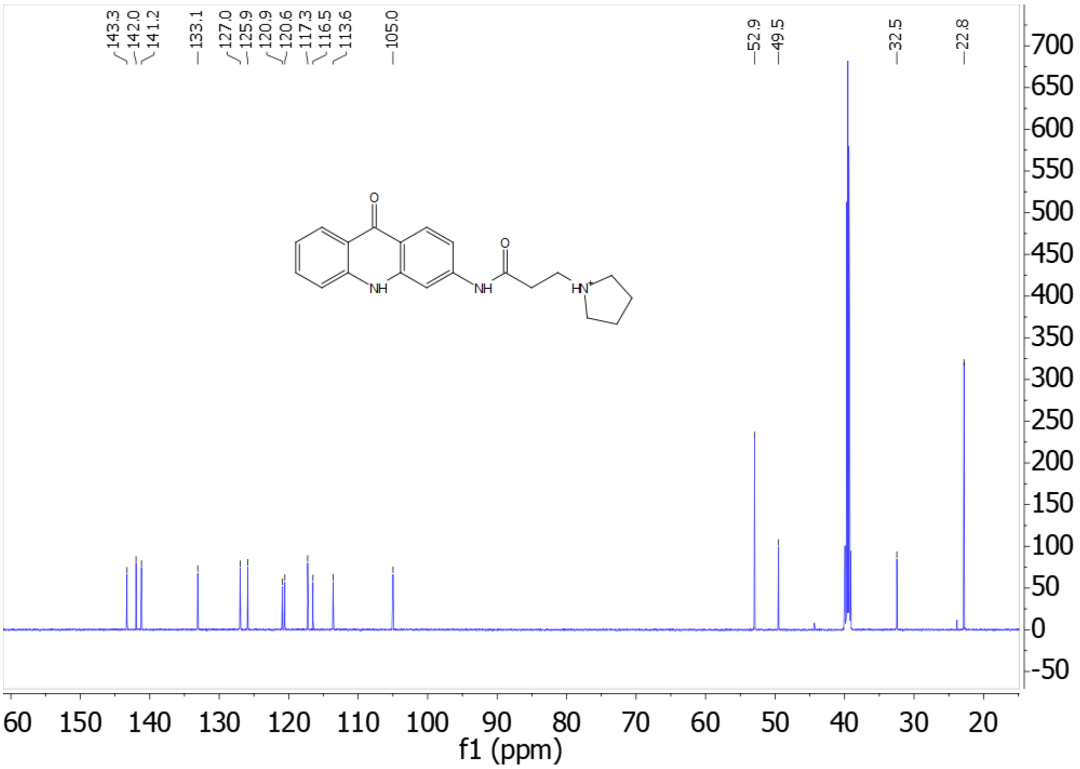

Figure S53. ^{13}C NMR spectra (DMSO-d_6, 150 MHz) of the acridines derivative **15** HCl salt.

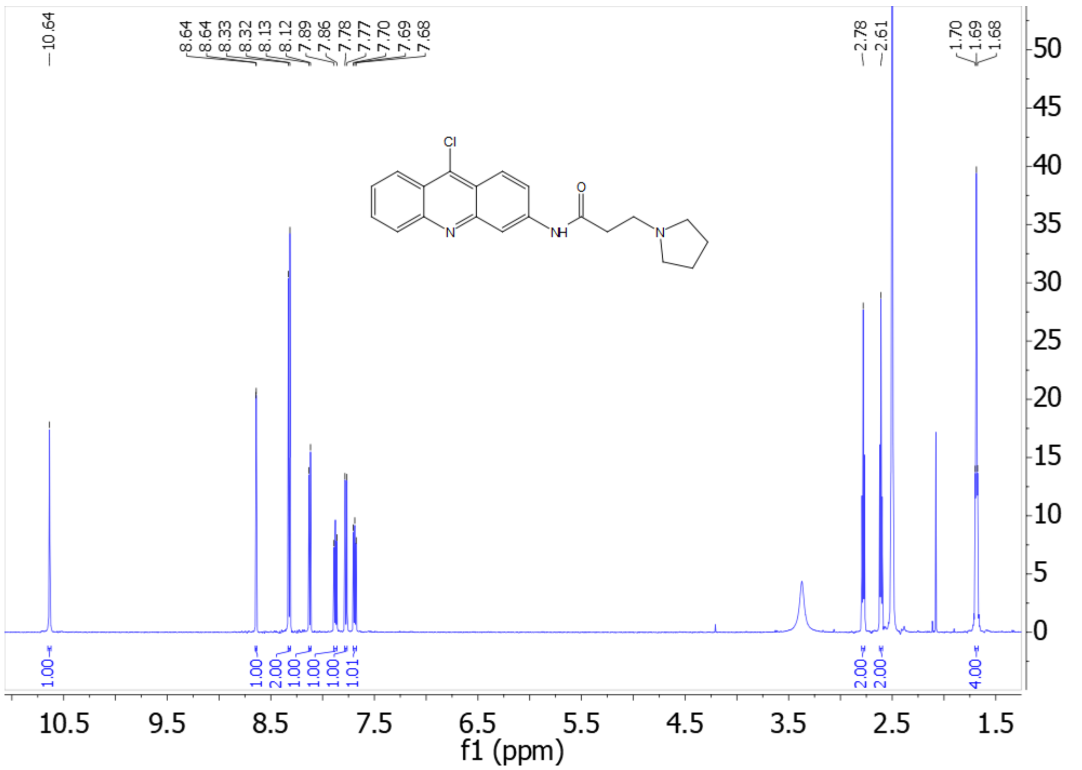

Figure S54. ^{1}H NMR spectra (DMSO-d_6, 600 MHz) of chloroacridine **16**.

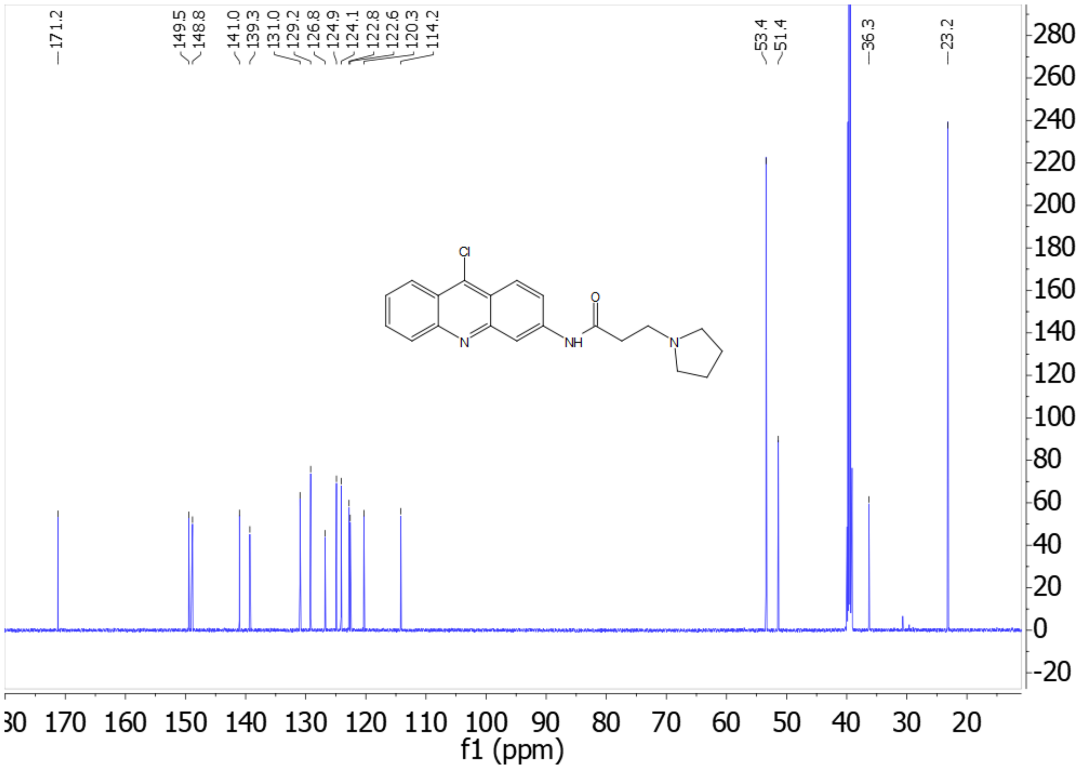

Figure S55. ^{13}C NMR spectra (DMSO-d_6, 150 MHz) of chloroacridine **16**.

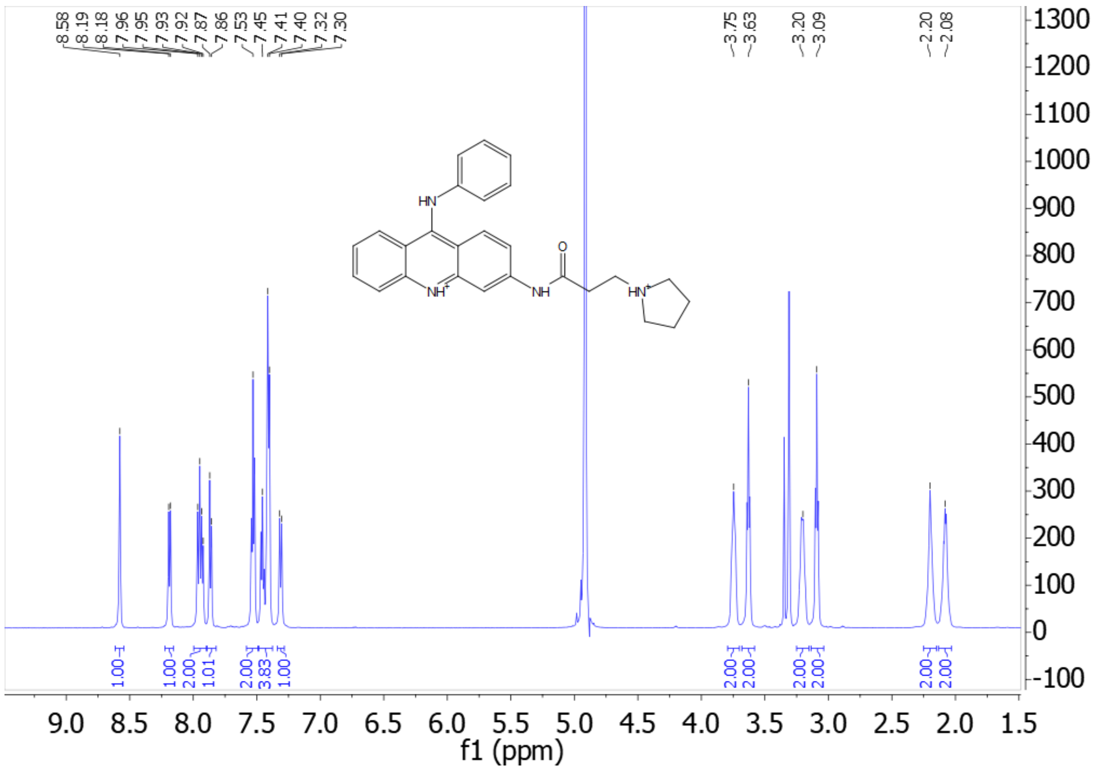

Figure S56. ^{1}H NMR spectra (Methanol-d_4, 600 MHz) of compound **17a** HCl salt.

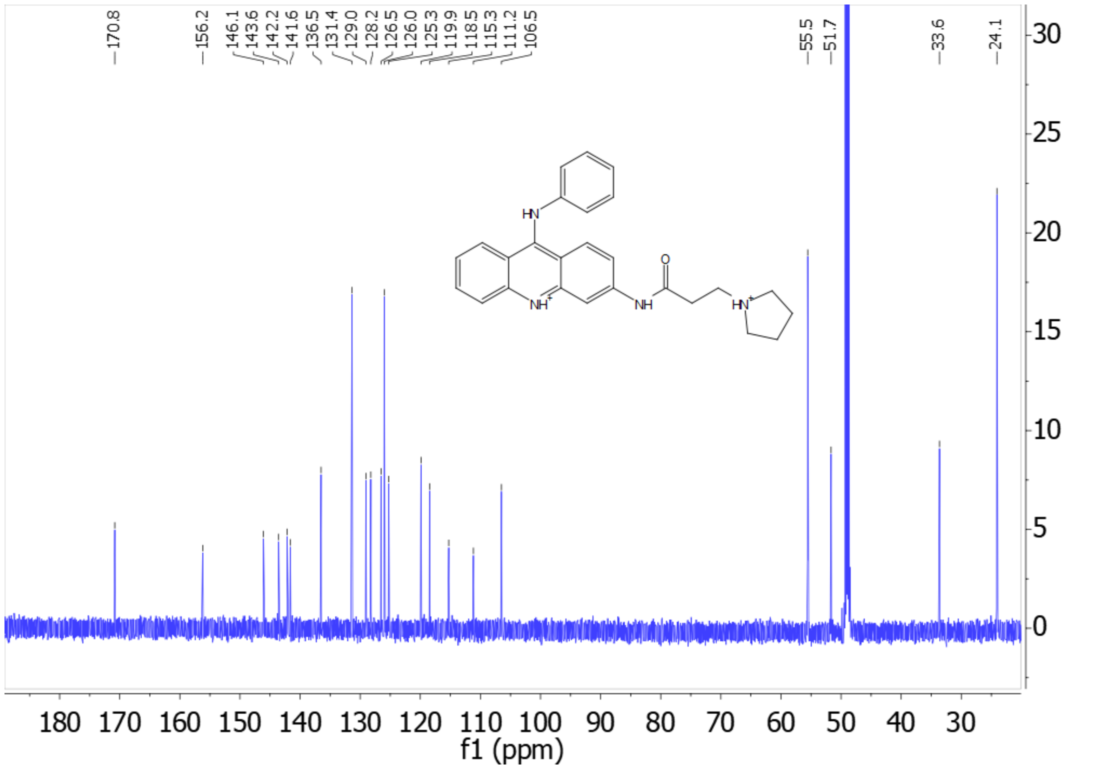

Figure S57. ^{13}C NMR spectra (Methanol-d_4, 150 MHz) of compound **17a** HCl salt.

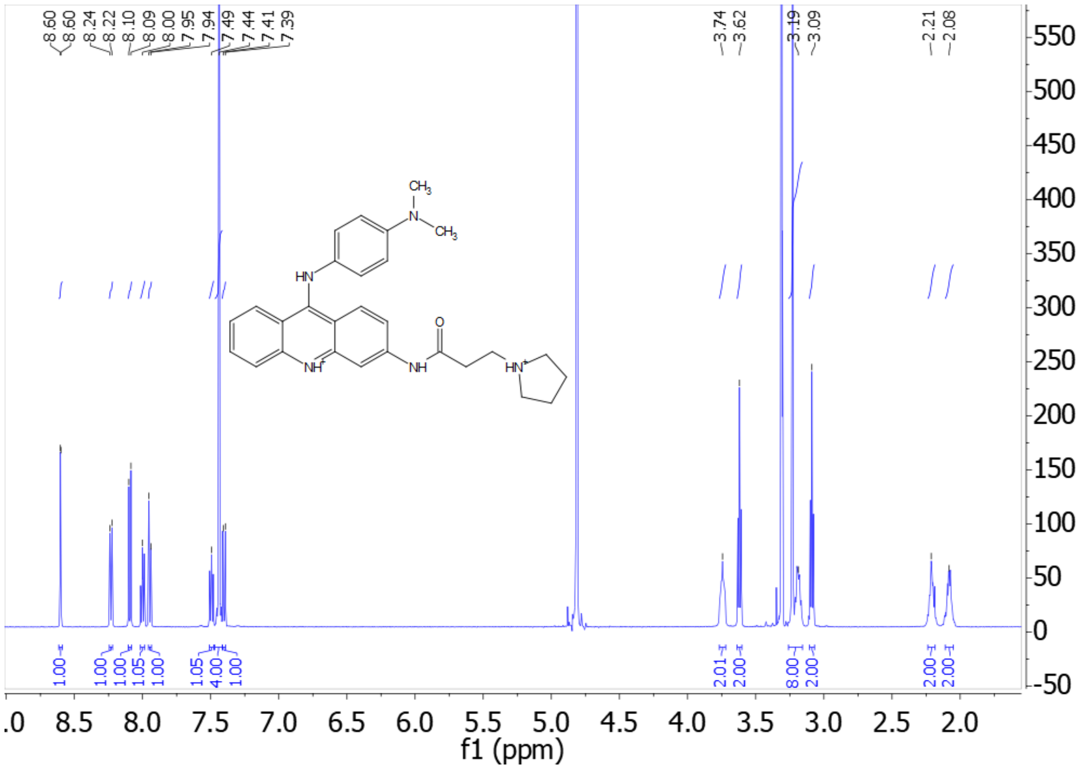

Figure S58. ^{1}H NMR spectra (Methanol-d_4 / D_2O (5:1), 600 MHz) of compound **17b** HCl salt.

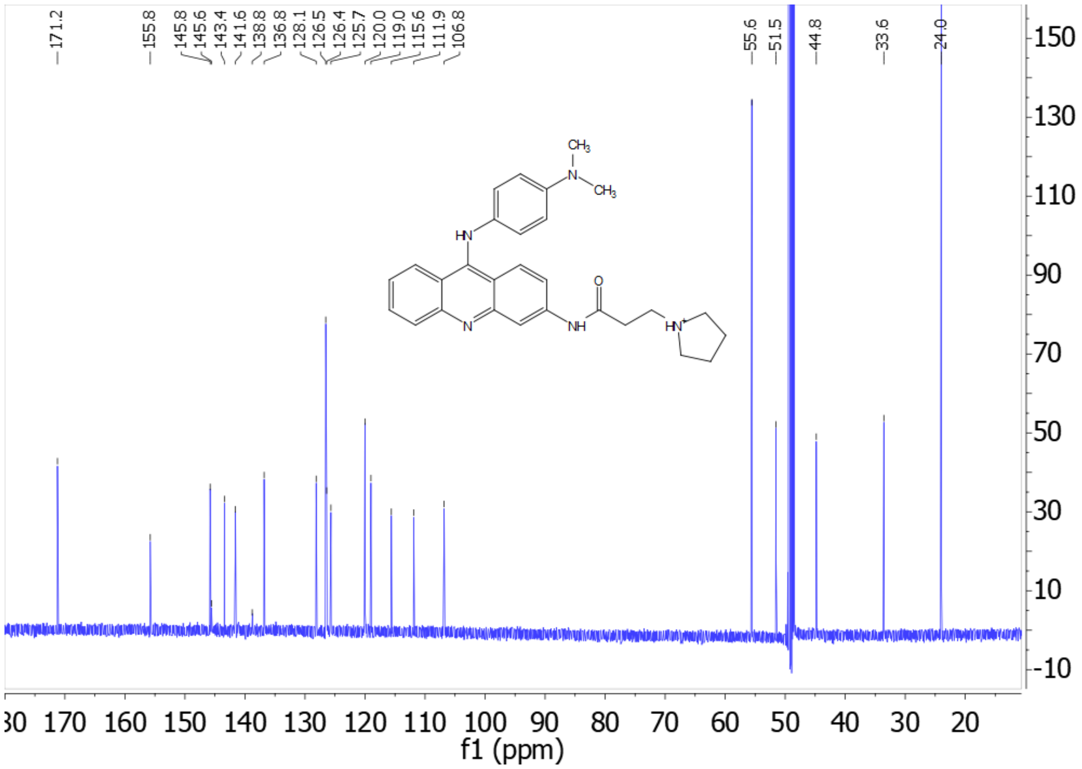

Figure S59. ^{13}C NMR spectra (Methanol-d_4 / D_2O (5:1), 150 MHz) of compound **17b** HCl salt.

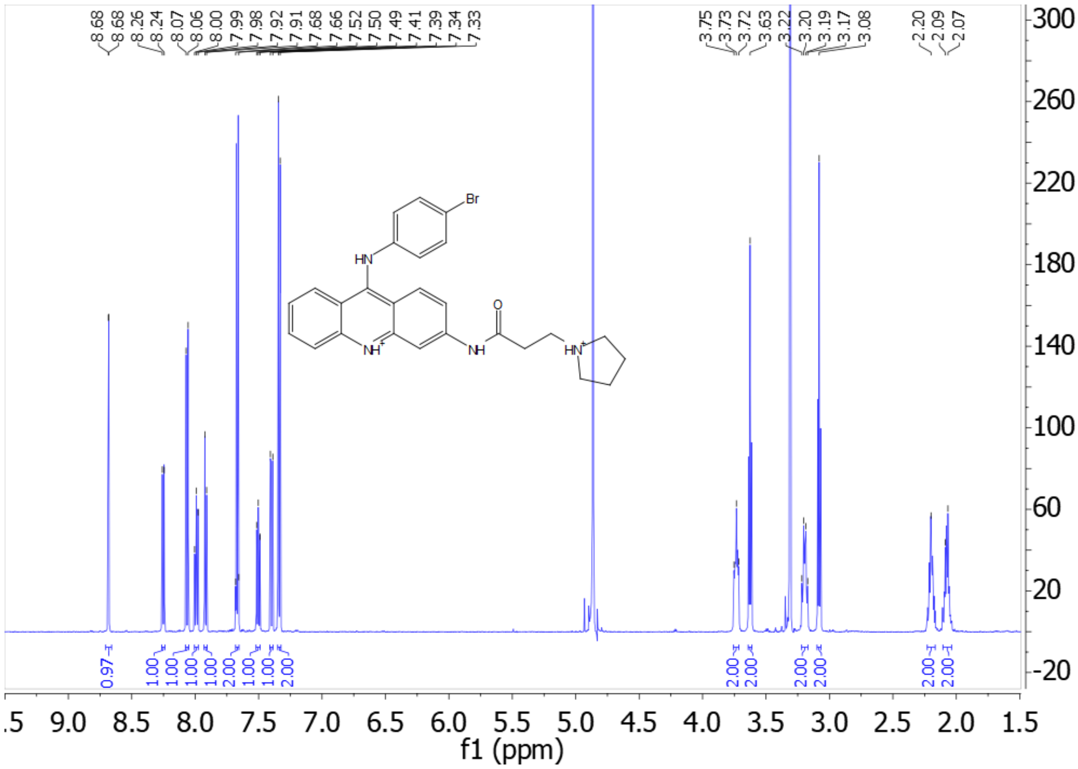

Figure S60. ^{1}H NMR spectra (Methanol-d_4, 600 MHz) of compound **17c** HCl salt.

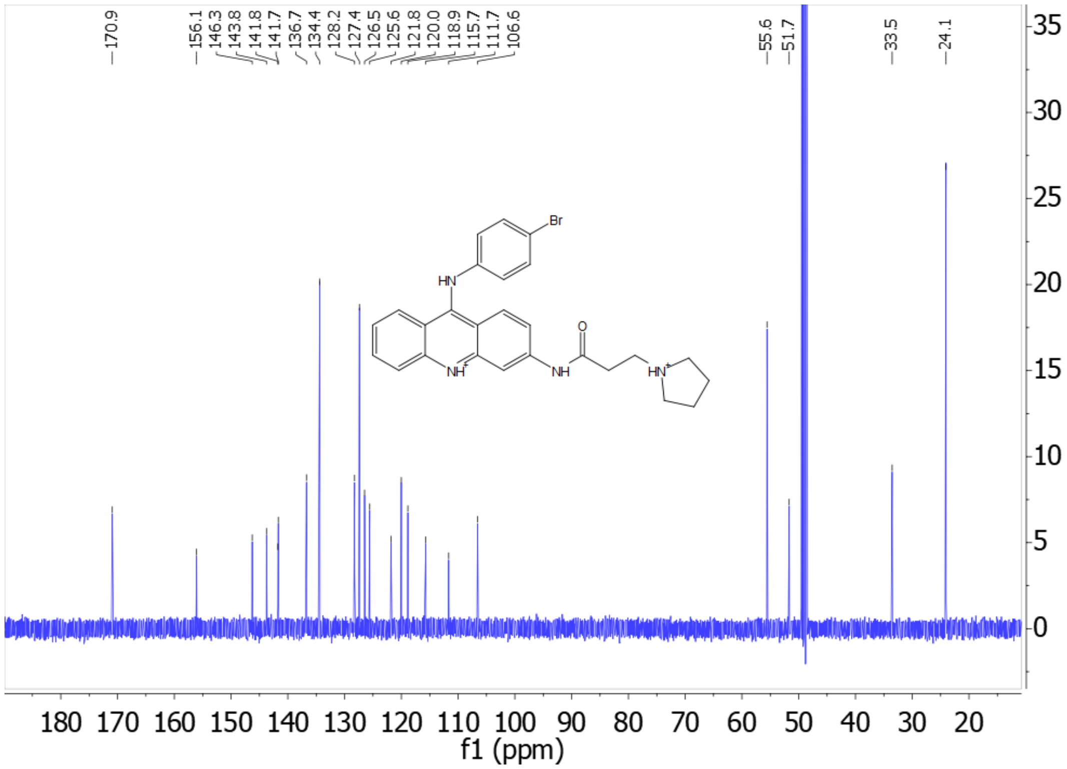

Figure S61. ^{13}C NMR spectra (Methanol-d_4, 150 MHz) of compound **17c** HCl salt.

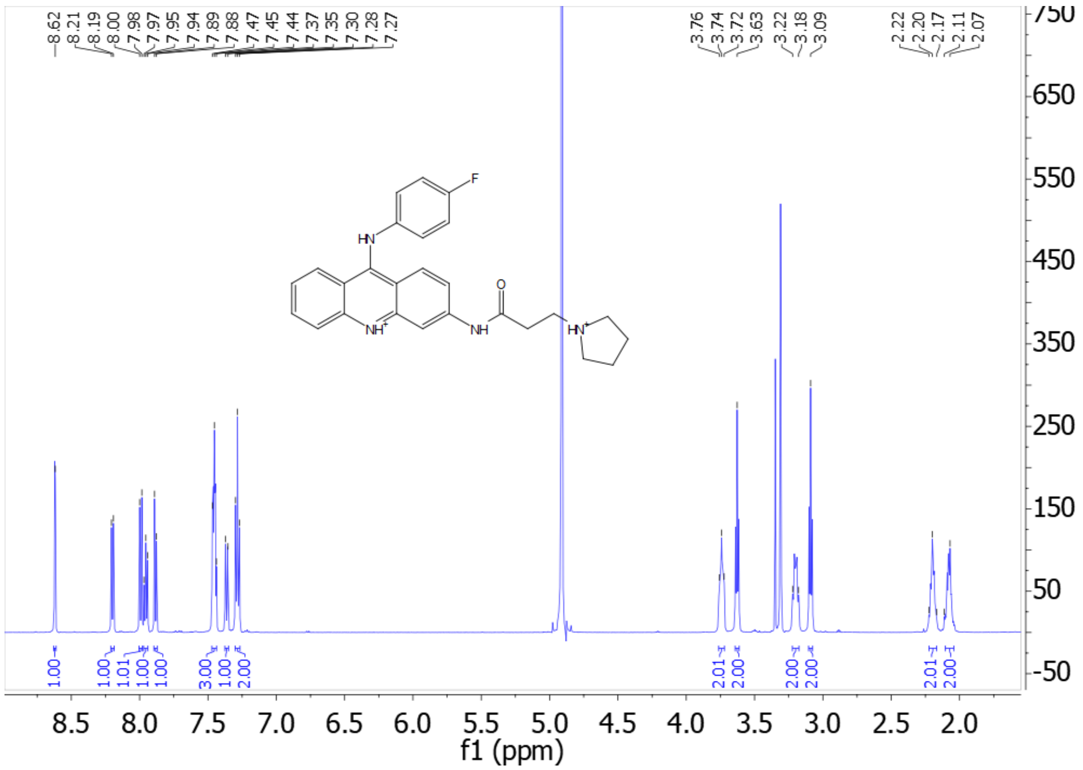

Figure S62. ^{1}H NMR spectra (Methanol-d_4, 600 MHz) of compound **17d** HCl salt.

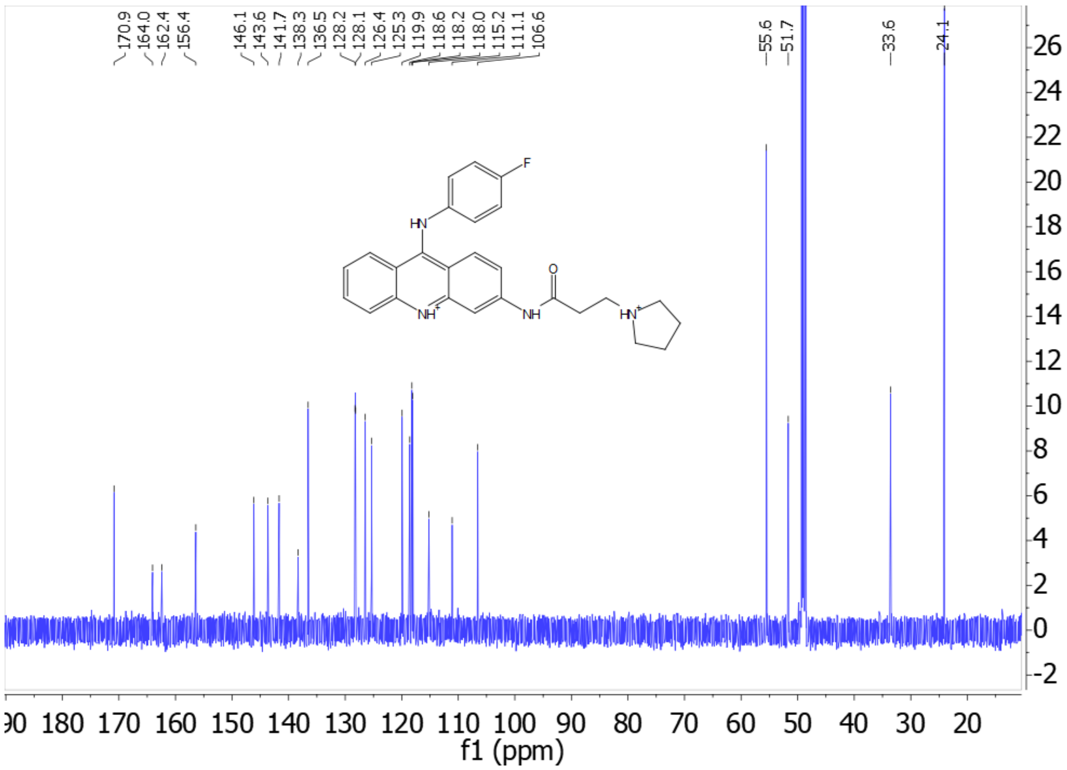

Figure S63. ^{13}C NMR spectra (Methanol-d_4, 150 MHz) of compound **17d** HCl salt.

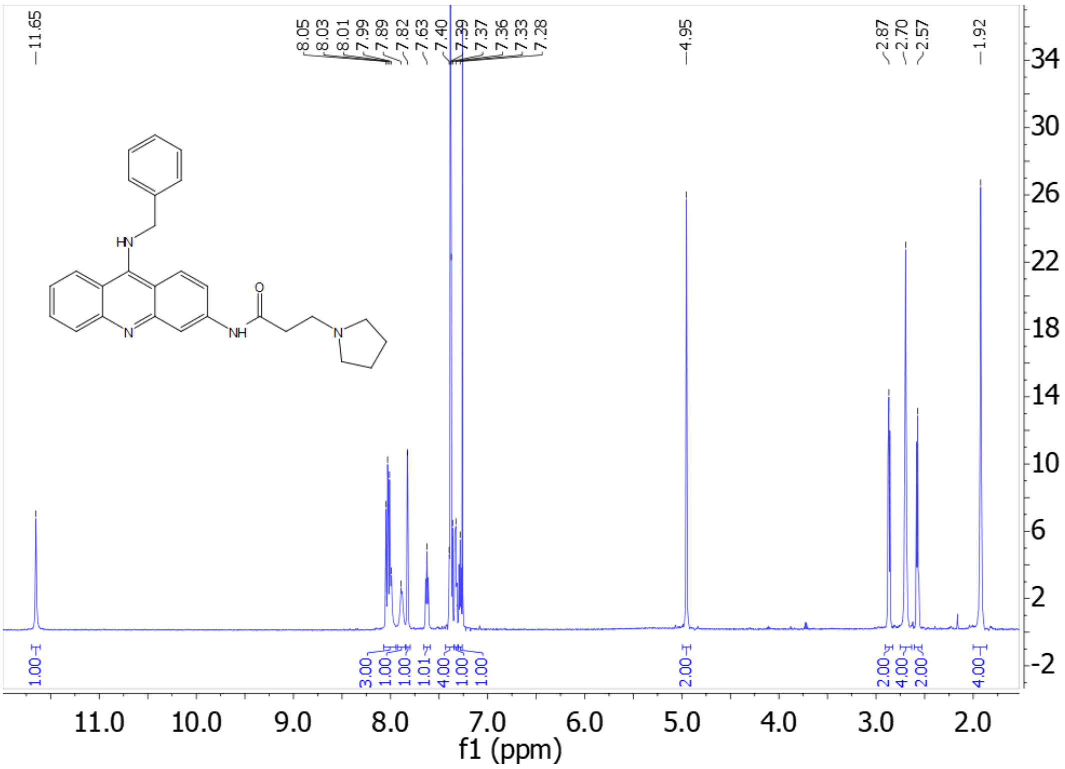

Figure S64. ^{1}H NMR spectra (Chloroform-*d*, 600 MHz) of compound **17e**.

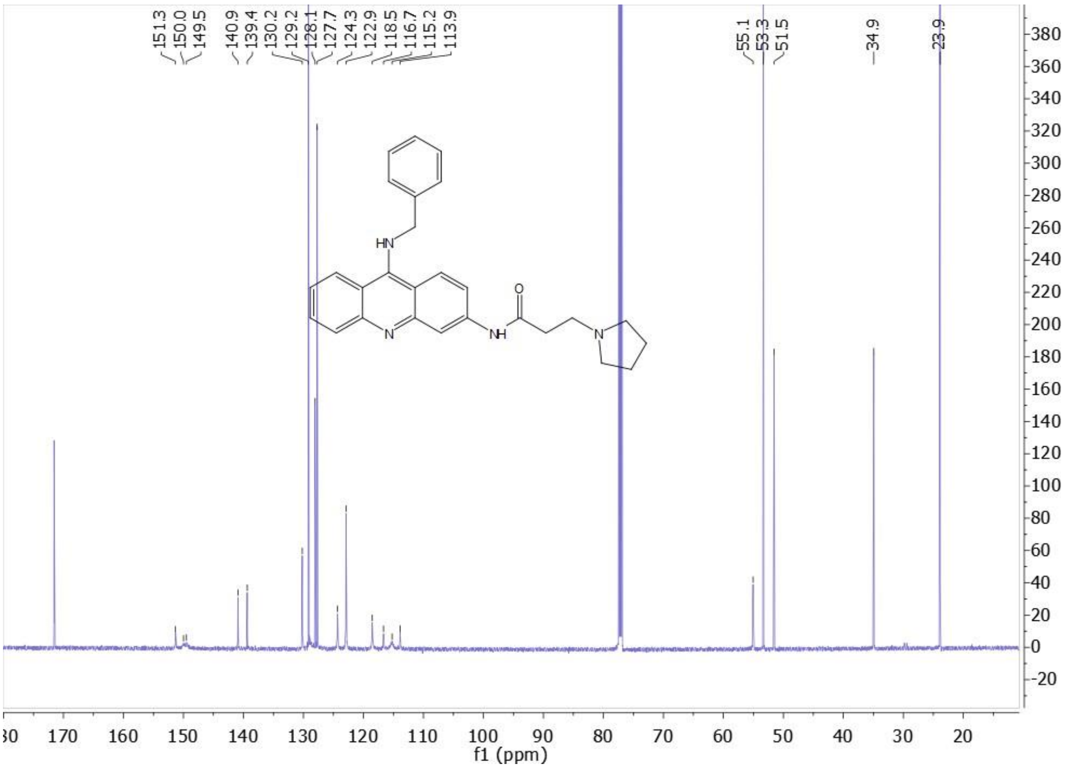

Figure S65. ^{13}C NMR spectra (Chloroform-*d*, 150 MHz) of compound **17e**.

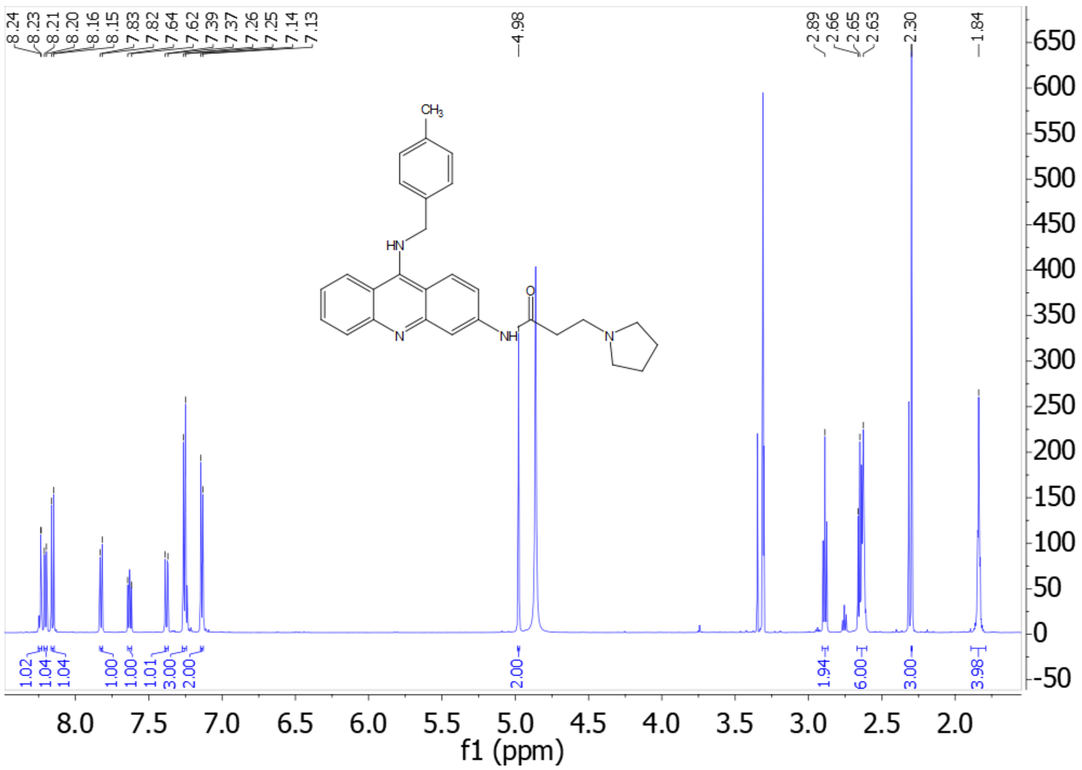

Figure S66. ^{1}H NMR spectra (Methanol-d_4, 600 MHz) of compound **17f**.

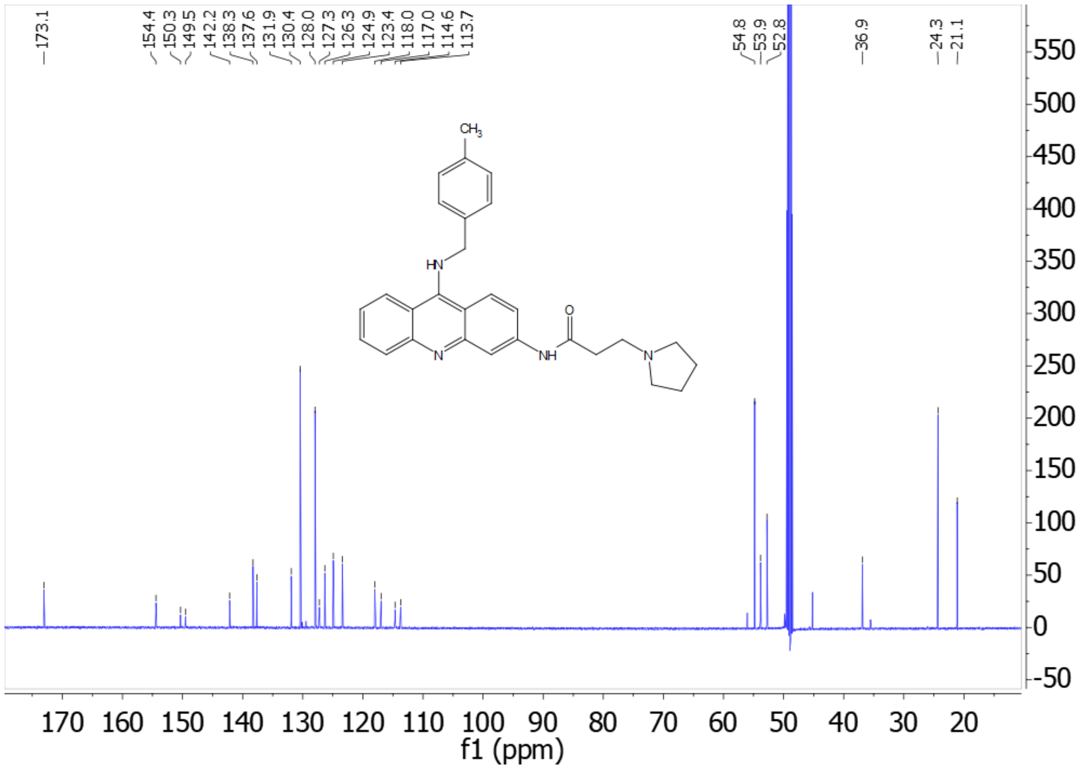

Figure S67. ^{13}C NMR spectra (Methanol-d_4, 150 MHz) of compound **17f**.

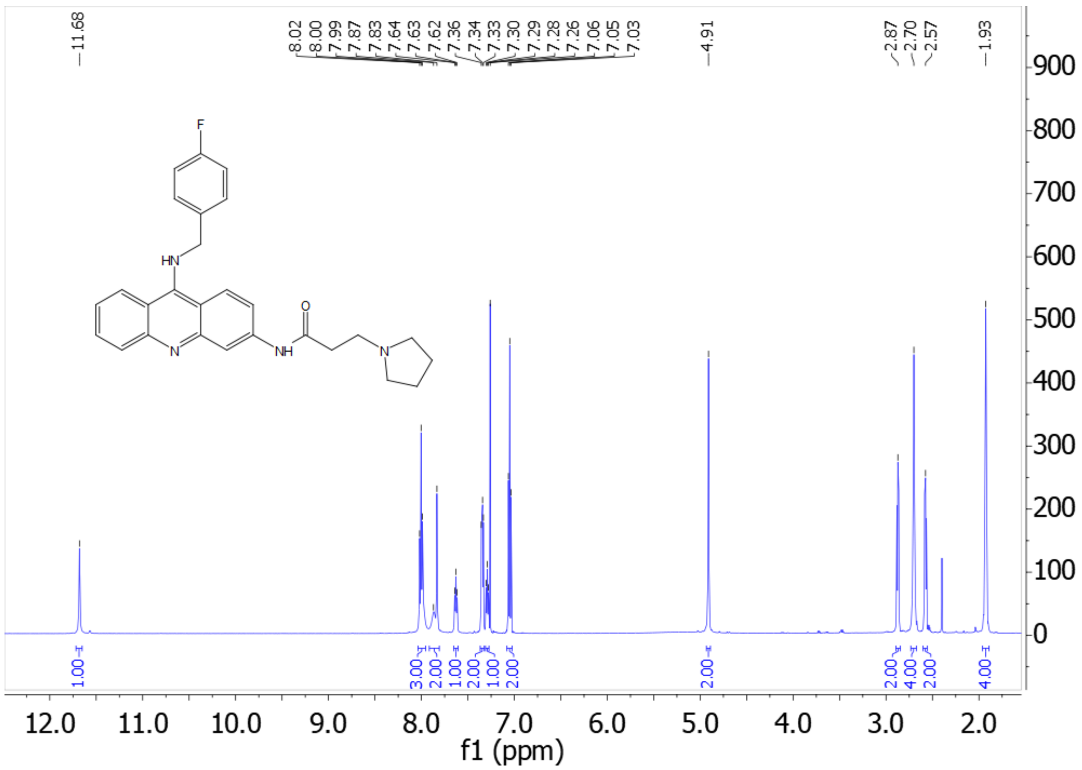

Figure S68. ^{1}H NMR spectra (Chloroform-*d*, 600 MHz) of compound **17g**.

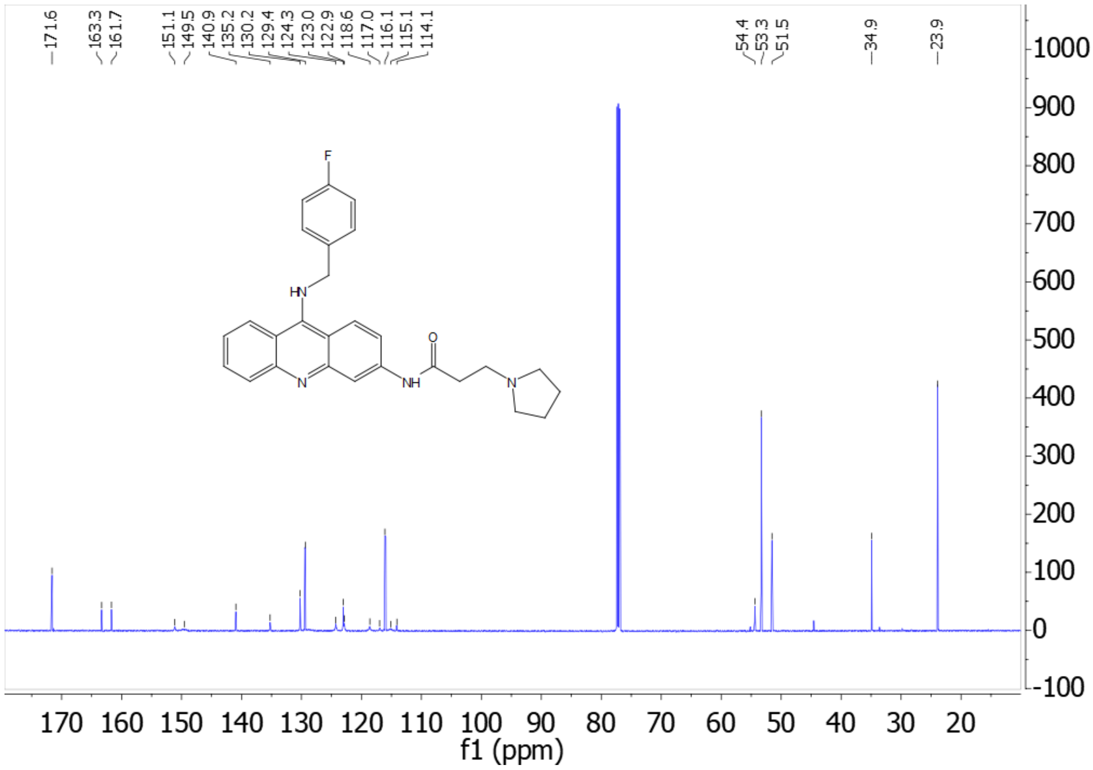

Figure S69. ^{13}C NMR spectra (Chloroform-*d*, 150 MHz) of compound **17g**.

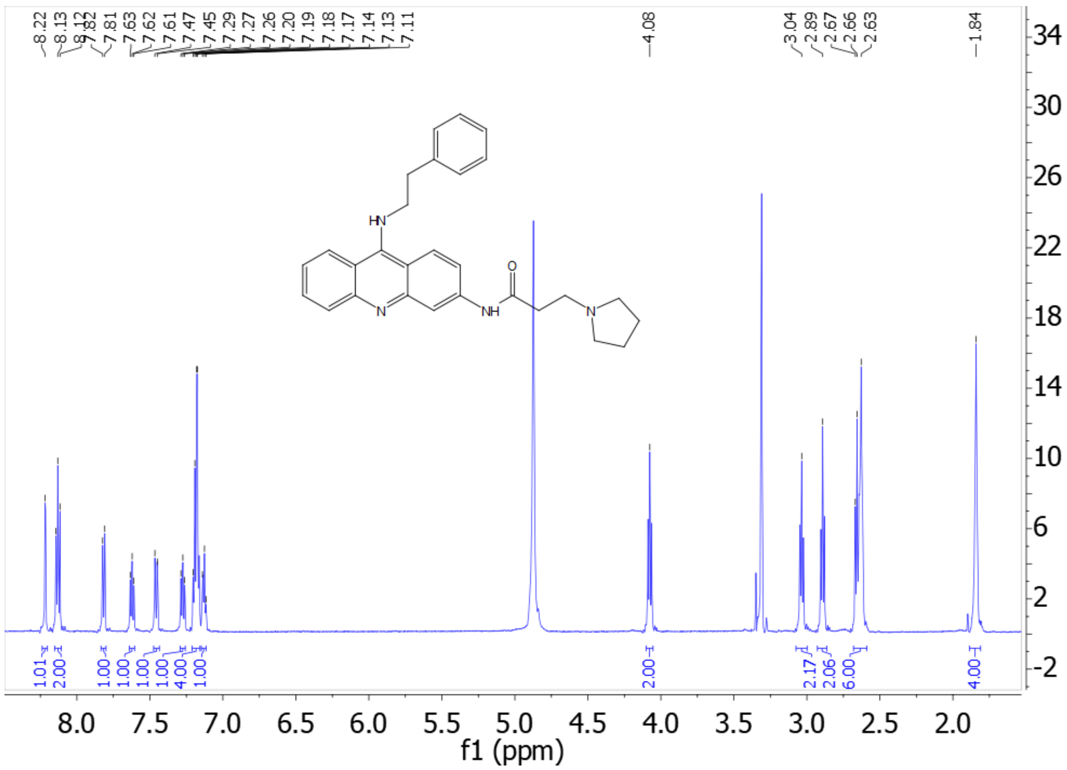

Figure S70. ^{1}H NMR spectra (Methanol-d_4, 600 MHz) of compound **17h**.

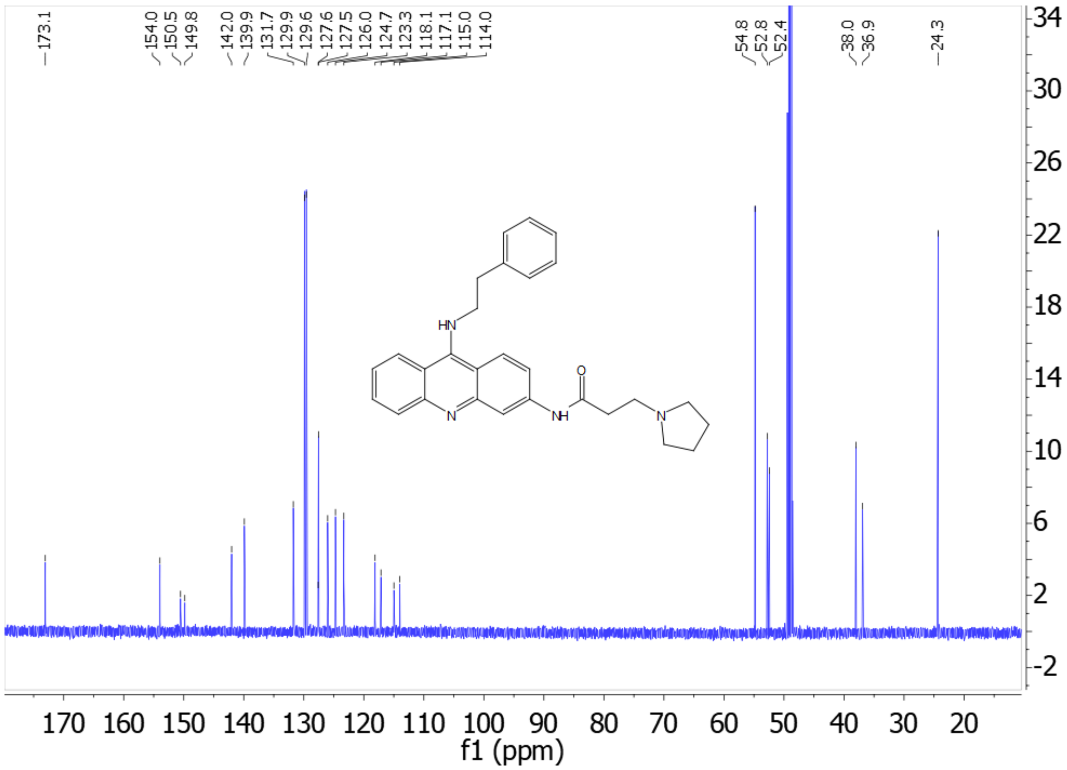

Figure S71. ^{13}C NMR spectra (Methanol-d_4, 150 MHz) of compound **17h**.

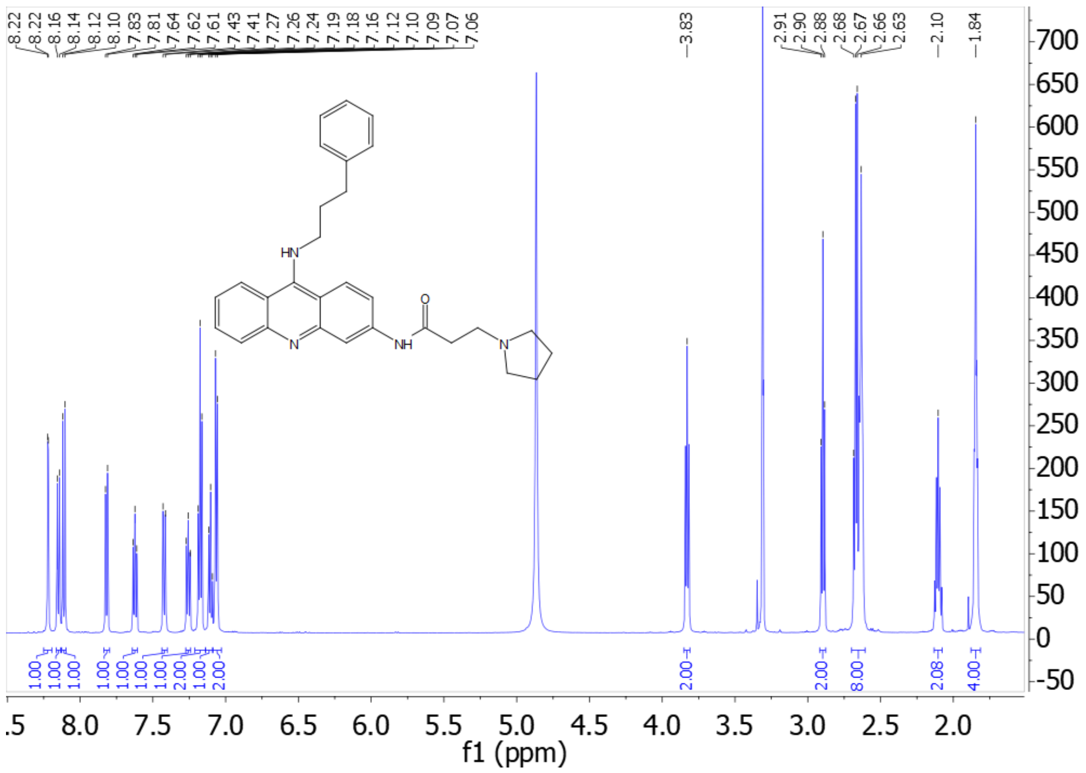

Figure S72. ^{1}H NMR spectra (Methanol-d_4, 600 MHz) of compound **17i**.

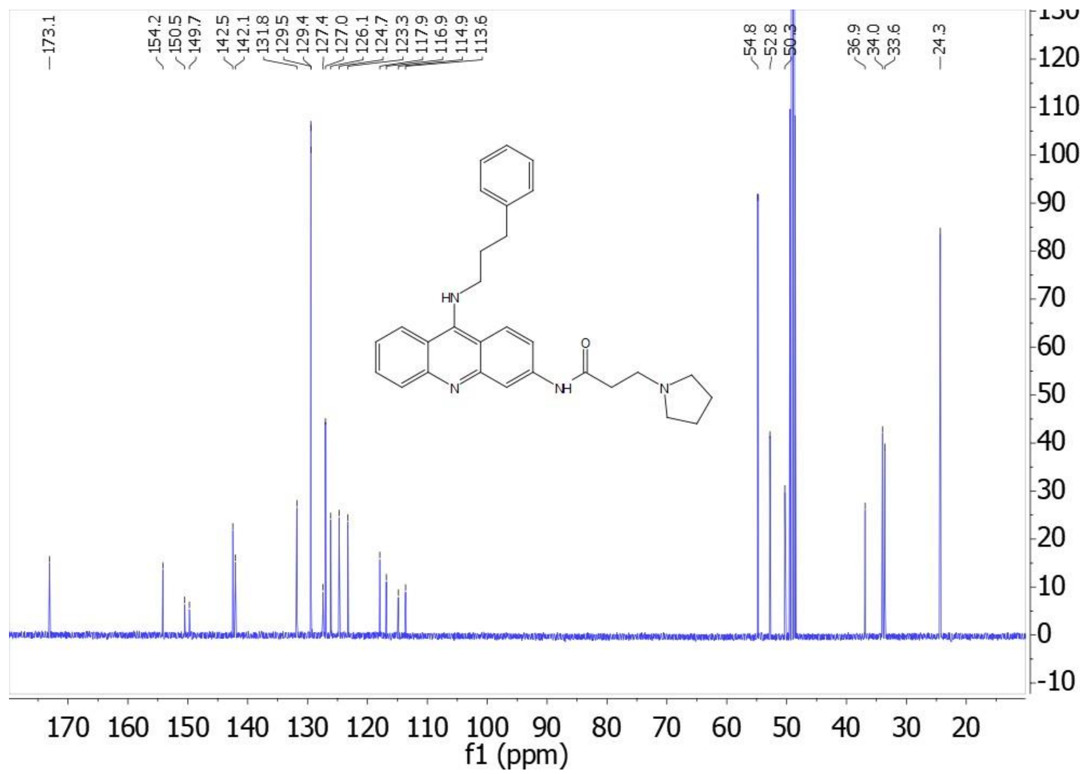

Figure S73. ^{13}C NMR spectra (Methanol-d_4, 150 MHz) of compound **17i**.

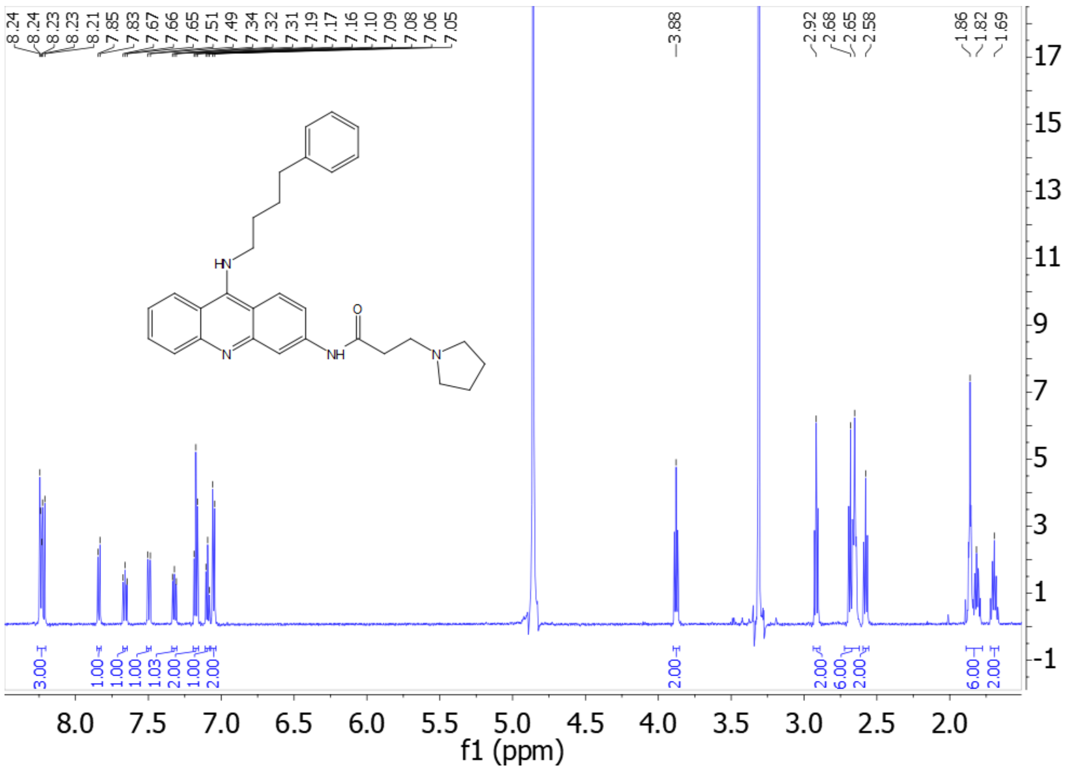

Figure S74. ^{1}H NMR spectra (Methanol-d_4, 600 MHz) of compound **17j**.

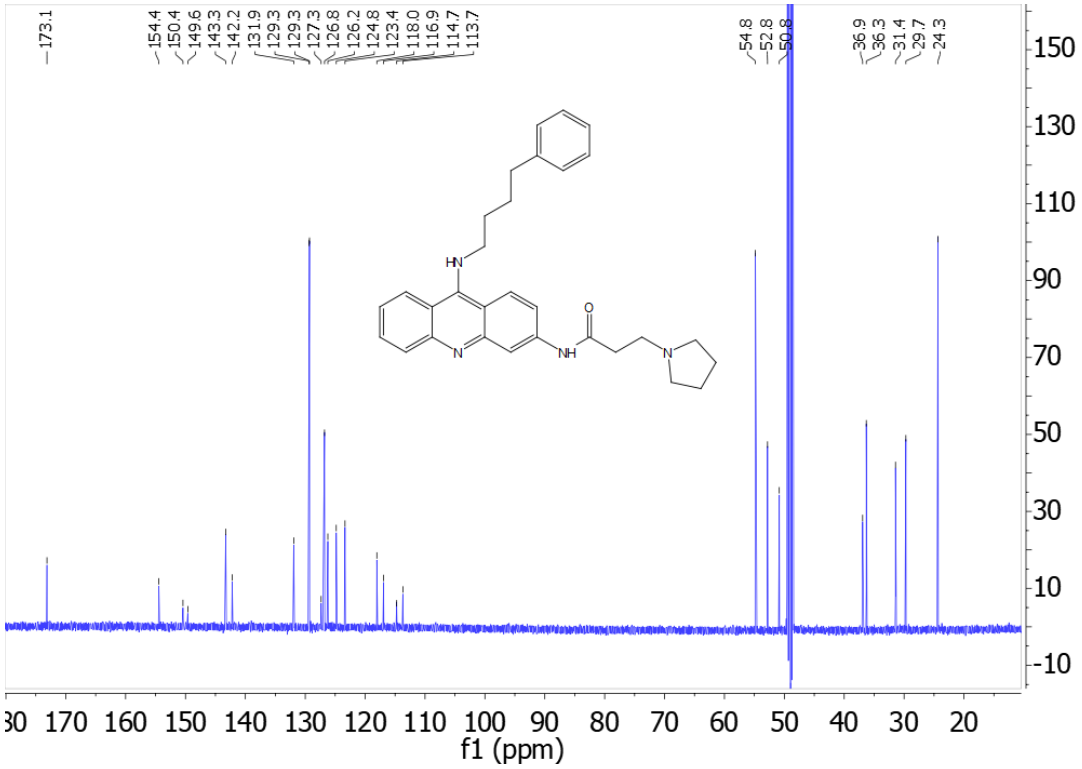

Figure S75. ^{13}C NMR spectra (Methanol-d_4, 150 MHz) of compound **17j**.

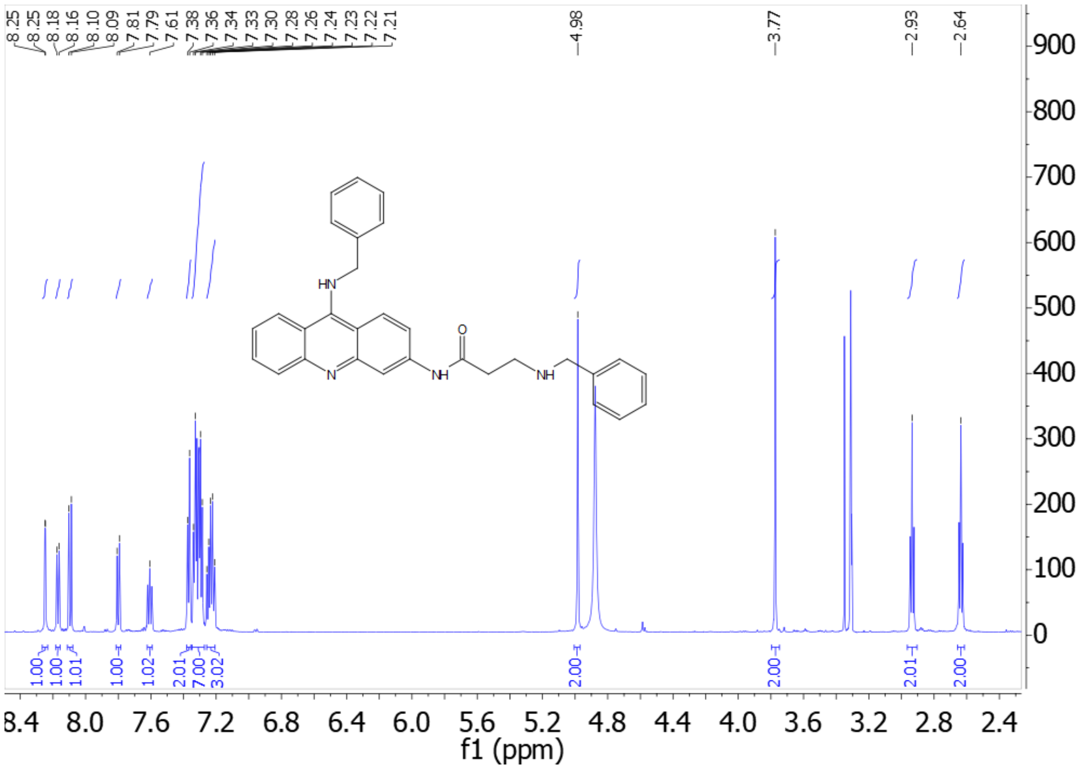

Figure S76. ^{1}H NMR spectra (Methanol-d_4, 600 MHz) of side product **17k**.

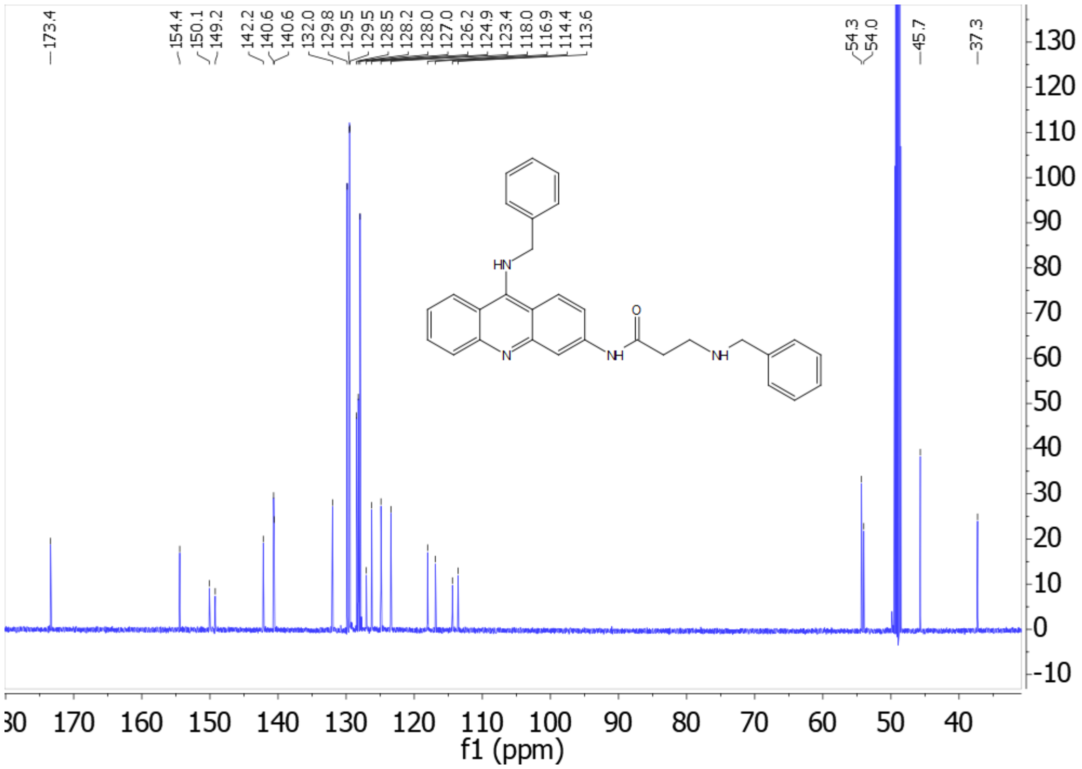

Figure S77. ^{13}C NMR spectra (Methanol-d_4, 150 MHz) of side product **17k**.

SUPPLEMENTARY REFERENCES

1. J. Ungvarsky, J. Plsikova, L. Janovec, J. Koval, J. Mikes, L. Mikesová, D. Harvanova, P. Fedorocko, P. Kristian, J. Kasparkova, V. Brabec, M. Vojtickova, D. Sabolova, Z. Stramova, J. Rosocha, J. Imrich, M. Kozurkova, *Bioorg. Chem*. **2014**, *57*, 13.
2. ACD/ChemSketch package 2020.2.0 www.acdlabs.com (freeware).
3. MOPAC2016, Version: 19.179L, James J. P. Stewart, Stewart Computational Chemistry, web-site: HTTP://OpenMOPAC.net.
4. A.-R. Allouche, *J. Comput. Chem*. **2011**, 32 174.
5. I. Sushko, S. Novotarskyi, R. Körner A.K. Pandey, M. Rupp, W. Teetz, S. Brandmaier, A. Abdelaziz, V. V. Prokopenko, V. Y. Tanchuk, R. Todeschini, A. Varnek, G. Marcou, P. Ertl, V. Potemkin, M. Grishina, J. Gasteiger, C. Schwab, I. I. Baskin, V. A. Palyulin, E. V. Radchenko, W. J. Welsh, V. Kholodovych, D. Chekmarev, A. Cherkasov, J. Aires-de-Sousa, Q. Y. Zhang, A. Bender, F. Nigsch, L. Patiny, A. Williams, V. Tkachenko, I. V. Tetko, *J. Comput. Aided Mol. Des.* **2011**, *25*, 533.
6. I. Sushko, E. Salmina, V. A. Potemkin, G. Poda, I. V. Tetko, *J. Chem. Inf. Model.* **2012**, *52*, 2310.
7. I. Oprisiu, S. Novotarskyi, I. V. Tetko, *J. Cheminform*. **2013**, *5*, e4.
8. I. V. Tetko, S. Novotarskyi, I. Sushko, V. Ivanov, A. E. Petrenko, R. Dieden, F. Lebon, B. Mathieu, *J. Chem. Inf. Model.* **2013**, *53*, 1990.
9. A. E. Hargrove, Z. Zhong, J. L. Sessier, E. V. Anslyn, *New J. Chem.* **2010**, *34*, 348.
10. H. Huang, P. Zhang, B. Yu, C. Jin, L. Ji, H. Chao, *Dalton Trans*. **2015**, *44*, 17335.
11. E. F. Pettersen, T. D. Goddard, C. C. Huang, G. S. Couch, D. M. Greenblatt, E. C. Meng, T. E. Ferrin, *J. Comput. Chem.* **2004**, *25*, 1605.
12. G. S. Couch, D. K. Hendrix, T. E. Ferrin, *Nucleic Acids Res.* **2006**, *34*, e29.
13. B. L. Staker, M. D. Feese, M. Cushman, Y. Pommier, D. Zembower, L. Stewart, A. B. Burgin, *J. Med. Chem.* **2005**, *48*, 2336.
14. Y. R. Wang, S. F. Chen, C. C. Wu, Y. W. Liao, T. S. Lin, K. T. Liu, Y. S. Chen, T. K. Li, T. C. Chien, N. L. Chan, *Nucleic Acids Res.* **2017**, *45*, 10861.
15. C. C. Wu, Y. C. Li, Y. R. Wang, T. K. Li, N. L. Chan, *Nucleic Acids Res.* **2013**, 41, 10630.
16. F. Michel, M. F. Sanner, *J. Mol. Graph. Model.* **1999**, *17*, 57.
17. J. A. Maier, C. Martinez, K. Kasavajhala, L. Wickstrom, K. E. Hauser, C. Simmerling, *J. Chem. Theor. Comput.* **2015**, *11*, 3696.
18. G. M. Morris, D. S. Goodsell, R. S. Halliday, R. Huey, W. E. Hart, R. K. Belew, A. J. Olson, *J. Comput. Chem.* **1998**, *19*, 1639.
19. R. Huey, G. M. Morris, A. J. Olson, D. S. Goodsell, *J. Comput. Chem.* **2007**, *28*, 1145.
